# Spatial and Temporal Trends in HIV/AIDS Burden Among Worldwide Regions From 1990 to 2019: A Secondary Analysis of the Global Burden of Disease Study 2019

**DOI:** 10.3389/fmed.2022.808318

**Published:** 2022-05-12

**Authors:** Huan He, Zejin Ou, Danfeng Yu, Yongzhi Li, Yuanhao Liang, Wenqiao He, Yuhan Gao, Fei Wu, Qing Chen

**Affiliations:** ^1^Guangdong Provincial Key Laboratory of Tropical Disease Research, Department of Epidemiology, School of Public Health, Southern Medical University, Guangzhou, China; ^2^Key Laboratory of Occupational Environment and Health, Guangzhou Twelfth People's Hospital, Guangzhou, China; ^3^Department of MICU, Guangdong Women and Children Hospital, Guangzhou, China

**Keywords:** acquired immune deficiency syndrome (AIDS), age-standardized rate (ASR), estimated annual percentage change (EAPC), epidemiological trend, global burden of disease (GBD), human immunodeficiency virus (HIV)

## Abstract

**Purpose:**

HIV/AIDS is a critical public health concern worldwide. This article investigated the spatial and temporal trends in HIV/AIDS burden from 1990 to 2019.

**Methods:**

Data were extracted from the Global Burden of Disease (GBD) Study 2019. The estimated annual percentage change (EAPC) and the age-standardized rate (ASR) were used to quantify the change in trends at the global, regional, and national levels.

**Results:**

In terms of temporal trends, during the period 1990–2004, increasing trends in prevalence (EAPC = 7.47, 95% confidence interval [CI] 5.84, 9.12), death (EAPC = 10.85, 95% CI 8.90–12.84), and disability-adjusted life years (DALYs) (EAPC = 10.40, 95% CI 8.47–12.36) of HIV/AIDS were observed. During the period 2005–2019, the global trends in HIV/AIDS incidence, death, and DALYs of HIV/AIDS decreased, with the EAPCs of −2.68 (95% CI−2.82–−2.53), −6.73 (95% CI −6.98–−6.47), and −6.75 (95% CI −6.95–−6.54), respectively. However, the disease prevalence showed a slight increasing trend (EAPC = 0.71, 95% CI 0.54–0.87). In terms of spatial trends, over the past 15 years, trends in HIV/AIDS incidence of HIV/AIDS appeared upward in High-middle and High sociodemographic index (SDI) areas (EAPC = 6.51, 95% CI 5.50–7.53; EAPC = 2.31, 95% CI 2.02–2.60, respectively).

**Conclusion:**

Decreasing trends in HIV/AIDS incidence, death, and DALYs have been observed worldwide over the past 15 years, especially in death and DALYs rates. However, the global population living with HIV/AIDS is still increasing. It is worth noting that an unfavorable trend emerged in High-middle and High SDI areas. Prevention and control of HIV/AIDS still need to be strengthened to counteract these concerning trends.

## Introduction

HIV/AIDS is one of the most important infectious diseases worldwide. Great achievements have been made in the control and the prevention of spread of HIV/AIDS, and these achievements are mainly due to the widespread use of antiretroviral agents in the treatment and prevention of HIV/AIDS (1, 2). For example, highly active combination antiretroviral therapy (ART) regimens have dramatically improved clinical outcomes and changed the spectrum of HIV-associated morbidity and mortality (3). Furthermore, the United Nations launched the Millennium Development Goals (MDGs) initiative and invested more than US$500 billion in the prevention and control of HIV/AIDS worldwide during the period 2000–2015 (4). Other prevention interventions, including early preventive interventions, official funding, enhancement in HIV/AIDS testing, and education, have also been advocated for to reduce the risk of HIV/AIDS transmission (5–9). The Joint United Nations Programme on HIV/AIDS (UNAIDS) launched the target of ending the AIDS epidemic by 2030, which proposed a 90% reduction in annual new HIV/AIDS infections and deaths in 2030 compared with 2010 (10). Notwithstanding, HIV/AIDS remains an important health problem globally (11). This was demonstrated by a 13% increase in new HIV/AIDS infections in Eastern Europe and Central Asia between 2006 and 2012 (9). Undoubtedly, sub-Saharan Africa still bears the greatest burden of HIV/AIDS. Botswana has one of the highest HIV prevalence rates in the world, with an adult HIV/AIDS prevalence rate of approximately 24% in 2005 (12). In 2013, although sub-Saharan Africa was home to only 12% of the global population, it accounted for 71% of the global burden of HIV infection (9). In 2017, there were 1.94 million new cases of HIV/AIDS globally and 36.8 million people living with HIV/AIDS, 40.5% of whom were not receiving ART (13).

The temporal variation patterns in HIV/AIDS incidence can help us better understand the epidemic factors affecting the disease and suggest more targeted measures of preventing and controlling the epidemic. The global burden of disease (GBD) study assessed the HIV/AIDS burden in 204 countries and territories across the world and provided a unique opportunity to understand the landscape of HIV/AIDS. In a recent study, Romona et al. described the global landscape of HIV/AIDS mortality using the data derived from the GBD Study 2019 (14). However, the author compared the burden across different countries using only the total number of cases and rates per 100,000 population, and only proposed the trend of change without providing more detailed information. Meanwhile, the analysis was limited to the national level, which could not reflect the trend changes of multiple geographic dimensions. Moreover, another study (13) focused only on new HIV infections and HIV/AIDS deaths from 1980 to 2017, which could not provide the total value of the change in HIV/AIDS disease burden.

Simply calculating the average value of the age-standardized rate (ASR) cannot provide a true and objective measure of the disease trends during this period (15). The estimated annual percentage change (EAPC) is the most used index to estimate the average annual rate of change in mortality, morbidity, and prevalence (16). The essential advantages of EAPC are the following. First, the reliability and accuracy of EAPC is higher, as all observations are considered. The calculation is more detailed, and the information utilization rate of the original data is higher. Moreover, the EAPC can more accurately explain the changing tendencies of a phenomenon with better stability and more generalizable conclusions. The EAPC undergoes statistical validation to verify whether the results are due to a random error or an actual change in trend. In general, the EAPC can more objectively and scientifically reflect changing trends and has a wider range of applications (17).

In the present study, a secondary analysis of the ASR was conducted to obtain the EAPC and further assessed the HIV/AIDS disease burden, including incidence, prevalence, death, and disability-adjusted life years (DALYs), and used the latest data to examine trends in HIV/AIDS burden from 1990 to 2019 in terms of temporal and spatial trends. Our results can serve as an important extension and complement to the previous study (14), as well as contribute to the development of country-specific HIV/AIDS prevention strategies.

## Methods

### Data Sources

The Global Health Data Exchange (GHDx) is a database created and supported by the Institute for Health Metrics and Evaluation (IHME), providing an interface for downloading GBD results data. Data on HIV/AIDS burden were derived from the GHDx (http://ghdx.healthdata.org/gbd-results-tool/). According to the instructions of the GBD 2019 online tool, the number and rate of incidence, prevalence, death, and DALYs were extracted between 1990 and 2010 based on age, sex, SDI areas, geographic regions, and country, without any inclusion/exclusion criteria. Our search terms included “HIV” or “AIDS,” and the subtypes “HIV-1”and “HIV-2.” Based on the socio-demographic index (SDI), the world was categorized into low, low-middle, middle, high-middle, and high regions. The available data reflected multiple geographic dimensions, including 21 geographic regions (e.g., East Asia, Oceania, and Caribbean) and 204 countries/territories (e.g., China, Brazil, and South Africa). Data on the Human Development Index (HDI) were downloaded from the United Nations Development Program (http://hdr.undp.org/en/data). This article does not contain any new studies involving human or animal subjects performed by any of the authors.

### Statistical Analysis

The statistically valid EAPC is an indicator of the steady rate of temporal change, with a positive or negative sign indicating the direction of change and the absolute value reflecting the average annual rate of change. Furthermore, the EAPC can predict disease expansion in the future, which provides scientifically valid information to policy makers and healthcare workers (17, 18). The EAPC and ASR were used to quantify the changing trends in disease burden. When considering potential discrepancies in age structure of multiple populations over time, the ASR is a necessary and representative index. The ASR was calculated according to the following formula:


$$\begin{array}{l} ASR=\frac{\sum_{i=1}^{A}a_{i}w_{i}}{\sum_{i=1}^{A}w_{i}}\times 100,000 \end{array}$$

where *a*_*i*_ is the age-specific rate in the *i*^th^ age group, *w* is the number of people in the corresponding *i*^th^ age group among the selected reference standard population, and *A* is the number of age groups. The ASR trends do not only represent the change in disease patterns of a population, but also serve as clues to changes in risk factors. The EAPC is a widely accepted method for describing the magnitude of the trends in ASR (15, 18, 19). A regression line is fitted to the natural logarithm of the ASR. The EAPC and its 95% CI were calculated through a linear regression model using the following formula:


$$\begin{array}{l} y=\alpha +\beta t+\varepsilon \end{array}$$

$$\begin{array}{l} \;EPAC=\left(\frac{{\hat{\text{ASR}}}_{\text{t}+1}-{\hat{\text{ASR}}}_{\text{t}}}{{\hat{\text{ASR}}}_{\text{t}}}\right)\cdot 100\\ =\left(\frac{{\hat{\text{ASR}}}_{\text{t}+1}}{{\hat{\text{ASR}}}_{\text{t}}}-1\right)\cdot 100=\left(\frac{e^{\hat{y}+\hat{\beta }}\left(t+1\right)}{e^{\hat{y}+\hat{\beta }}\cdot t}-1\right)\cdot 100\\ \;=\left(exp\left(\beta \right)-1\right)\times 100 \end{array}$$

where y = ln (ASR) and t = calendar year. Both the EAPC value and its 95% CI > 0 indicated an increasing trend, and both the EAPC value and its 95% CI < 0 indicated a decreasing trend. Other values indicated that the trend was stable over time. To detect factors influencing EAPCs, the associations between EAPCs and ASRs (1990, 2005), and HDI (2004, 2019) were assessed at the national level. Data analysis was conducted using R program (version 3.6.2) and IBM SPSS Statistics 22.0. Choropleth maps were drawn using the R program.

## Results

### Temporal Change Trends in HIV/AIDS

**Figure 1:**
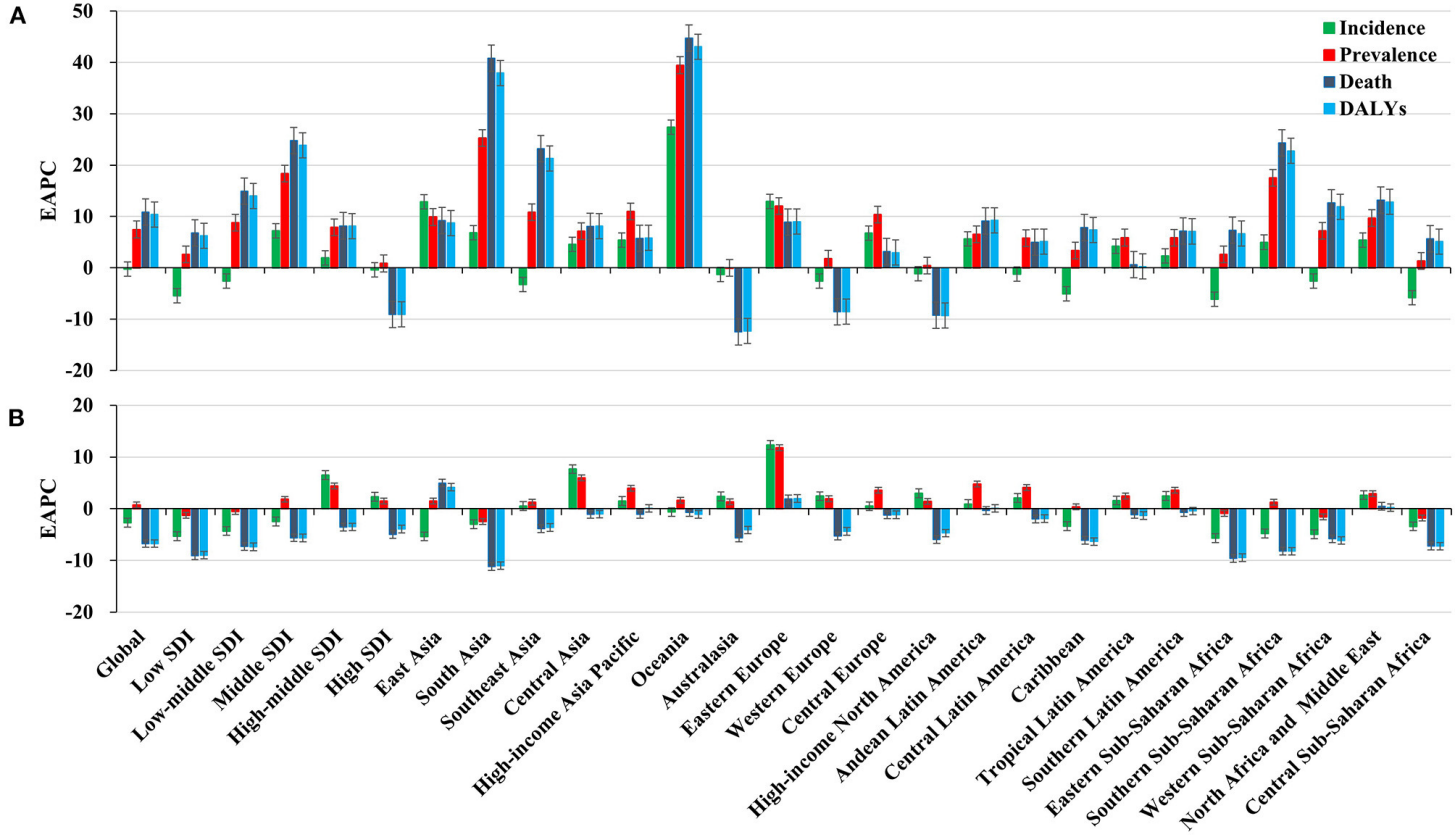
Trends in HIV/AIDS disease burden, including incidence, prevalence, death, and DALYs worldwide across SDI areas and geographic regions. **(A)** Distribution of the EAPCs of HIV/AIDS from 1990 to 2004; **(B)** Distribution of the EAPCs of HIV/AIDS from 2005 to 2019. EAPC, estimated annual percentage change.

The incidence, mortality, prevalence, and DALYs of HIV/AIDS varied significantly in different regions of the world between 1990 and 2019. Generally, the overall incidence was relatively stable during 1990–2004. However, the mortality, prevalence, and DALYs of HIV/AIDS showed a significant increase between 1990 and 2004. Strong decreasing trends in incidence, death, and DALYs were observed worldwide during the period 2005 and 2019 (Figure 1).

During the period 1990–2004, the global incident number of HIV/AIDS cases increased by 28.62% (Table 1 and Figure 1A). Compared with men, women presented higher incident numbers in 2004 (1,434.95 × 10^3^) (Table 1). The highest incident number was observed in the age group of 25–29 years (487.92 × 10^3^) and in children aged under 5 years (408.96 × 10^3^) in 2004, with the largest increase in proportion occurring in children aged under 5 years (124.05%) (Supplementary Table 1, Supplementary Figure 1A). The prevalence of HIV/AIDS cases increased by 258.90%, and the ASR showed a significant upward trend globally, with an EAPC of 7.47 (95% CI 5.84–9.12) (Figure 1A and Supplementary Table 2). Compared with men, women had a greater prevalence of HIV/AIDS and a more pronounced increasing trend, in which the EAPC was 8.06 (95% CI 6.23–9.91) (Supplementary Table 2). The prevalence rate of HIV/AIDS cases increased across all age groups, particularly in the 10–14 years age group (15,030.00%) (Supplementary Table 1, Supplementary Figure 2A). In this period, the ASR of HIV/AIDS death showed a rising trend worldwide (EAPC = 10.85, 95% CI 8.90–12.84) (Figure 1A and Supplementary Table 3). Compared with men, more deaths were reported among women, which indicated a pronounced increase in trend, with an EAPC of 11.89 (95% CI 9.76–14.06) (Supplementary Table 3). During the period 1990–2004, the proportion of deaths due to HIV/AIDS increased for all age groups, particularly for the 10–14 years age group (3,480.81%) (Supplementary Table 1, Supplementary Figure 3A). Pronounced increasing trends were observed in DALYs, with an EAPC of 10.40 (95% CI 8.47–12.36). During this period, increasing trends in DALYs were observed in both men and women (Figure 1A and Supplementary Table 4). The DALYs of HIV/AIDS increased in all age groups, particularly in those aged 10–14 years (3,587.85%) (Supplementary Table 1, Supplementary Figure 4A).

**Table 1:** Percentage changes in the absolute number and the EAPCs of HIV/AIDS incidence from 1990 to 2019 globally, stratified by sexes, SDI areas, and geographic regions.

**Characteristics**	**2004**		**1990–2004**		**2019**		**2005–2019**	
	**Number × 10^3^ (95% UI)**	**ASR/100,000** **(95% UI)**	**Change in number (%)**	**EAPC** **(95% CI)**	**Number × 10^**3**^ (95% UI)**	**ASR/100, 000** **(95% UI)**	**Change in number (%)**	**EAPC** **(95% CI)**
**Overall**	2,646.64 (2,312.99–3,124.83)	39.50 (34.48–46.65)	28.62	−0.26 (−1.68–1.18)	1,989.28 (1,760.91–2,259.35)	25.24 (22.39–28.57)	−22.77	−2.68 (−2.82–−2.53)
**Sex**								
Male	1,211.69 (1,068.64–1,424.91)	36.17 (31.83–42.51)	31.73	−0.10 (−1.46–1.28)	990.70 (877.73–1,110.29)	24.82 (22.02–27.84)	−16.16	−2.17 (−2.32–−2.02)
Female	1,434.95 (1,227.63–1,713.94)	42.98 (36.76–51.31)	26.11	−0.39 (−1.85–1.09)	998.58 (861.84–1,165.36)	25.74 (22.29–30.00)	−28.37	−3.14 (−3.30–−2.98)
**SDI**								
Low	811.11 (678.94–990.31)	109.51 (89.94–136.65)	−22.88	−5.43 (−5.97–−4.89)	507.62 (387.05–670.69)	47.96 (36.36–63.13)	−35.66	−5.29 (−5.61–−4.98)
Low-middle	687.62 (579.15–834.73)	46.64 (38.95–57.35)	11.26	−2.56 (−4.27–−0.81)	431.48 (357.16–524.54)	23.68 (19.52–28.90)	−34.45	−4.31 (−4.38–−4.24)
Middle	848.49 (748.78–989.75)	38.88 (34.29–45.21)	260.70	7.21 (4.03–10.49)	650.91 (572.94–746.74)	25.84 (22.80–29.35)	−21.60	−2.46 (−2.60–−2.32)
High-middle	106.53 (95.59–118.48)	7.72 (6.92–8.64)	51.09	1.92 (−0.12–4.01)	231.74 (197.05–278.73)	15.13 (13.00–18.30)	116.70	6.51 (5.50–7.53)
High	67.86 (46.16–89.24)	7.21 (4.76–9.60)	−15.06	−0.41 (−1.84–1.04)	94.03 (50.41–137.75)	9.32 (5.10–13.43)	41.57	2.31 (2.02–2.60)
**Regions**								
East Asia	65.94 (42.89–98.11)	4.36 (2.82–6.55)	548.63	12.87 (11.69–14.06)	34.37 (16.15–55.58)	2.30 (1.18–3.80)	−48.56	−5.35 (−6.31–−4.37)
South Asia	141.76 (85.92–217.59)	9.38 (5.49–14.70)	395.59	6.86 (−0.43–14.69)	87.91 (50.08–165.04)	4.66 (2.63–8.55)	−29.94	−2.99 (−3.54–−2.43)
Southeast Asia	97.42 (76.57–118.71)	16.26 (12.46–20.19)	8.30	−3.26 (−4.44–−2.07)	126.78 (107.46–165.92)	18.11 (15.18–23.93)	29.35	0.51 (0.03–0.99)
Central Asia	2.84 (2.25–3.39)	3.47 (2.82–4.07)	104.82	4.59 (3.36–5.84)	7.39 (5.09–9.86)	7.16 (4.95–9.50)	166.10	7.67 (6.40–8.95)
High–income Asia Pacific	4.10 (2.53–5.88)	1.94 (1.20–2.74)	124.60	5.38 (3.10–7.71)	4.57 (2.52–6.79)	2.15 (1.29–3.11)	18.38	1.50 (0.92–2.09)
Oceania	6.52 (0.22–17.07)	72.14 (2.47–190.28)	3,841.19	27.38 (18.38–37.07)	7.59 (0.22–25.00)	58.21 (1.64–191.7)	21.72	−0.63 (−0.89–−0.37)
Australasia	1.01 (0.68–1.41)	4.16 (2.70–6.00)	−15.25	−1.33 (−2.58–−0.07)	1.64 (0.99–2.39)	5.75 (3.38–8.47)	66.09	2.38 (1.99–2.77)
Eastern Europe	40.03 (33.83–50.13)	18.51 (15.57–23.25)	247.47	12.94 (9.97–15.99)	155.63 (125.72–195.4)	71.58 (58.95–89.74)	278.91	12.31 (10.38–14.28)
Western Europe	18.99 (15.3–23.17)	4.58 (3.60–5.71)	−32.70	−2.55 (−3.51–−1.57)	25.06 (18.67–31.99)	6.18 (4.49–7.94)	31.18	2.44 (2.08–2.81)
Central Europe	2.44 (2.10–2.91)	2.07 (1.79–2.48)	172.79	6.79 (5.26–8.33)	1.99 (1.57–2.73)	1.91 (1.5–2.64)	−9.91	0.49 (0.02–0.97)
High–income North America	46.33 (29.27–63.26)	14.29 (8.69–19.75)	−25.09	−1.16 (−2.95–0.66)	70.47 (30.78–109.12)	19.78 (8.85–30.13)	56.23	3.00 (2.58–3.42)
Andean Latin America	7.27 (5.93–9.54)	14.20 (11.41–18.77)	203.70	5.61 (4.34–6.90)	11.32 (8.22–17.20)	17.09 (12.57–25.92)	52.55	0.89 (0.04–1.76)
Central Latin America	25.39 (23.63–27.78)	11.80 (11.20–12.63)	8.67	−1.19 (−2.31–−0.06)	39.72 (32.94–49.76)	15.08 (12.61–18.74)	52.61	2.07 (1.81–2.33)
Caribbean	25.20 (20.3–31.5)	59.07 (47.52–73.81)	−37.67	−5.04 (−5.63–−4.45)	17.54 (12.00–25.17)	36.14 (24.76–51.8)	−29.07	−3.38 (−3.78–−2.98)
Tropical Latin America	46.28 (37.16–54.71)	23.22 (18.36–27.96)	70.94	4.23 (2.61–5.88)	66.08 (51.39–80.5)	27.34 (21.47–33.42)	39.43	1.60 (1.04–2.15)
Southern Latin America	9.66 (5.94–14.63)	16.72 (10.22–25.54)	51.71	2.33 (1.9–2.77)	16.57 (9.00–26.30)	24.75 (13.09–39.63)	67.06	2.45 (2.09–2.82)
Eastern Sub–Saharan Africa	839.42 (715.68–1,007.85)	318.60 (265–393.1)	−21.76	−6.10 (−6.79–−5.40)	506.66 (384.62–660.63)	132.72 (100.76–175.17)	−37.96	−5.66 (−5.97–−5.35)
Southern Sub–Saharan Africa	778.50 (681.34–917.97)	1,134.81 (983.44–1,355.85)	236.71	4.99 (1.45–8.66)	441.52 (351.73–549.58)	528.20 (420.92–658.5)	−41.66	−4.77 (−5.13–−4.41)
Western Sub–Saharan Africa	380. (307.21–487.81)	139.75 (111.58–182.59)	35.77	−2.61 (−4.31–−0.88)	260.76 (214.03–324.62)	65.30 (53.31–81.71)	−29.48	−4.94 (−4.98–−4.89)
North Africa and Middle East	13.13 (7.55–24.74)	2.77 (1.58–5.08)	230.32	5.41 (4.03–6.8)	24.14 (9.82–63.61)	3.74 (1.53–9.63)	82.12	2.65 (2.35–2.95)
Central Sub–Saharan Africa	93.99 (72.07–121.28)	120.23 (91.21–157.53)	−29.85	−5.82 (−6.06–−5.59)	81.57 (55.01–116.92)	70.12 (46.73–100.97)	−11.51	−3.41 (−3.50–−3.32)

*ASR, age-standardized rate; CI, confidence interval; EAPC, estimated annual percentage change; HIV/AIDS, Human immunodeficiency virus infection/acquired immune deficiency syndrome; SDI, socio-demographic index; UI, uncertainty interval*.

The trends in HIV/AIDS disease burden changed from 2015 to 2019. The incident number of HIV/AIDS cases declined from 22.77% during the period 2005–2019 to 1,989.28 × 10^3^ [95% uncertainty interval (UI): 1,760.91 × 10^3^, 2,259.35 × 10^3^] in 2019. The ASR of incidence decreased by an annual average of 2.68% (EAPC = −2.68, 95% CI −2.82–−2.53) (Table 1 and Figure 1B). A decreasing trend in the ASR of HIV/AIDS incidence was more obvious in women than in men, with an EAPC of −3.14 (95% CI −3.30–−2.98) (Table 1). Among different age groups, the highest increase in the number of HIV/AIDS cases was observed in the age group>60 years (19.83%), and the highest decrease was observed in the group aged under 5 years (−67.8%) (Supplementary Table 5, Supplementary Figure 1A). Globally, the prevalence of HIV/AIDS cases increased by 30.31% from 2005 to 2019, and it was 36,848.15 × 10^3^ (95% UI: 35,149.00 × 10^3^, 38,856.67 × 10^3^) in 2019. The increased trend in HIV/AIDS prevalence was demonstrated with an EAPC of 0.71 (95% CI 0.54–0.87) (Figure 1B and Supplementary Table 2). The ASR of HIV/AIDS prevalence increased for both sexes, especially in women (EAPC = 0.76, 95% CI 0.58–0.94) (Supplementary Table 2), and the percentage prevalence of HIV/AIDS cases increased for most age groups, especially for individuals aged >80 years (263.55%), whereas the largest decrease was observed for children aged <5 years (−55.79%) (Supplementary Table 5, Supplementary Figure 2A). During this period, the number of deaths due to HIV/AIDS decreased by 52.89%, corresponding to 863.84 × 10^3^ deaths (95% UI 786.07 × 10^3^, 996.04 × 10^3^) worldwide in 2019. Globally, the ASR of deaths showed an obvious downward trend from 2005 to 2019, with an EAPC of −6.73 (95% CI −6.98–−6.47) (Figure 1B and Supplementary Table 3). Decreasing HIV/AIDS trends were observed in both sexes (Supplementary Table 3). Across different age groups, the mortality rates due to HIV/AIDS decreased in all age groups, except for those aged >80 years (0.01%). The highest decrease in percentage change occurred in children aged <5 years (−76.53%) (Supplementary Table 5, Supplementary Figure 3A). Globally, the number of DALYs due to HIV/AIDS was 47,632.18 × 10^3^ (95% UI: 42,630.99 × 10^3^, 55,650.04 × 10^3^) in 2019, with 54.03% decrease since 2005. The ASR of DALYs showed a decreasing trend worldwide from 2005 to 2019 (EAPC = −6.75, 95% CI −6.95–−6.54) (Figure 1B and Supplementary Table 4). Compared with men, women showed a higher decreasing trend, in which the EAPC was −7.44 (95% CI −7.65–−7.22) (Supplementary Table 4). The proportion of the number of DALYs due to HIV/AIDS decreased across all age groups, except for individuals aged > 80 years (19.37%) (Supplementary Table 5, Supplementary Figure 4A).

### Spatial Change Trends in HIV/AIDS

**Figure 2:**
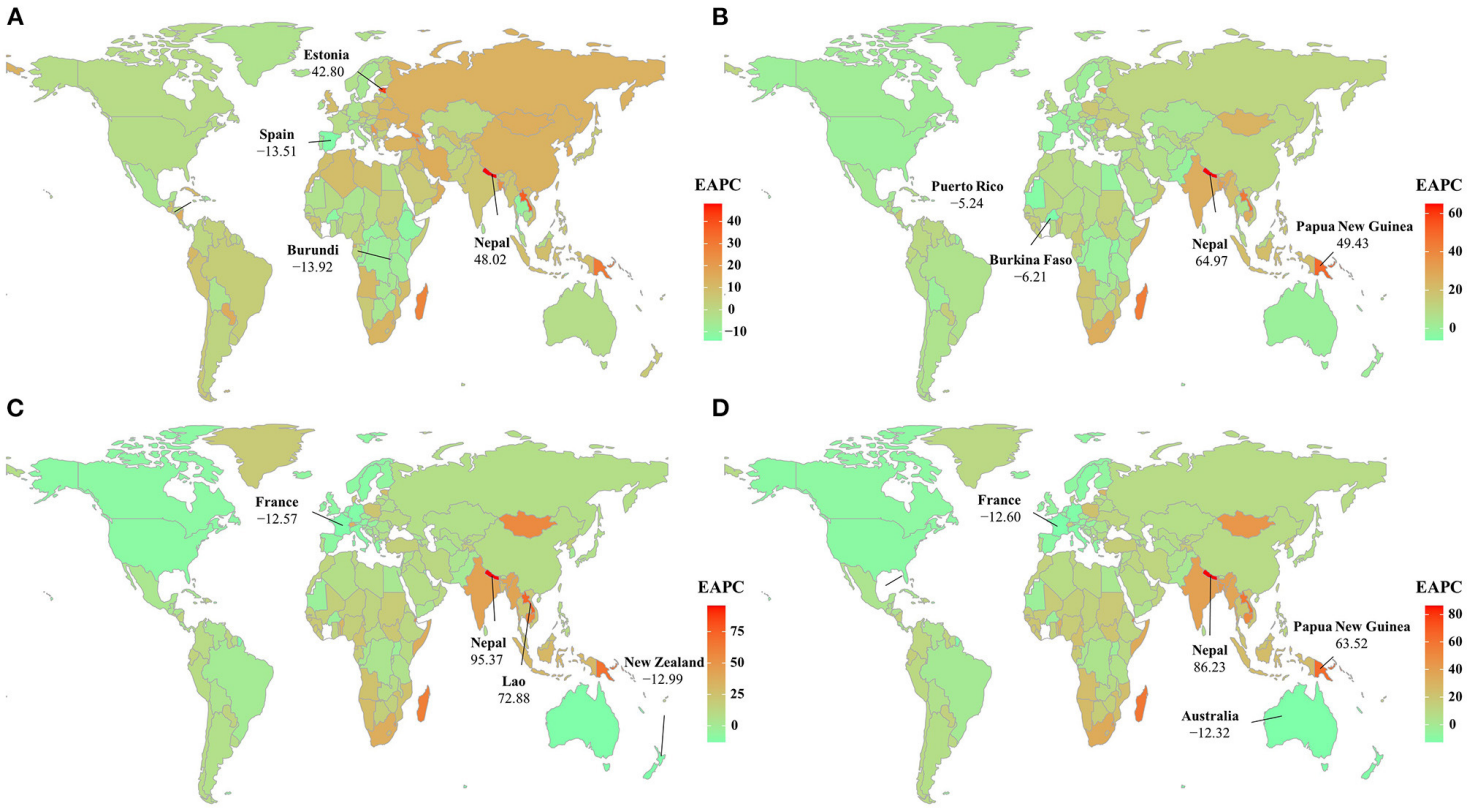
The distribution of the EAPCs of HIV/AIDS burden at the national level during the period 1990–2004, including the incidence **(A)**, prevalence **(B)**, deaths **(C)**, and DALYs **(D)**. Countries/territories with extreme values are annotated. ASR, age-standardized rate; EAPC, estimated annual percentage change; DALYs, disability-adjusted life years.

During 1990–2004, the ASR of HIV/AIDS incidence declined rapidly in low SDI areas (EAPC = −5.43, 95% CI −5.97–−4.89), but an increasing incidence was observed in middle SDI areas (EAPC = 7.21, 95% CI 4.03–10.49). At the regional level, an increasing trend in HIV/AIDS was observed in 11 regions, particularly in Oceania and Eastern Europe, with EAPCs of 27.38 (95% CI 18.38–37.07) and 12.94 (95% CI 9.97–15.99), respectively (Table 1 and Supplementary Figures 1B,C). At the national level, increasing trends of HIV/AIDS were observed in 104 countries/territories, with the fastest rise in Nepal, Estonia, and the Lao People's Democratic Republic, in which the respective EAPCs were 48.02 (95% CI 37.33–59.55), 42.80 (95% CI 39.60–46.08), and 36.94 (95% CI 32.01–42.04). Conversely, decreasing trends were observed in 61 countries/territories and the most pronounced changes occurred in Burundi and Spain, with the respective EAPCs of −13.92 (95% CI −14.61–−13.21) and −13.51 (95% CI −16.10–−10.84) (Figure 2A and Supplementary Table 6, Supplementary Figures 5A, 6A). Increasing trends in prevalence occurred in most SDI areas and geographic regions, particularly in Oceania and South Asia, in which the respective EAPCs were 39.48 (95% CI 34.22–44.95) and 25.27 (95% CI 19.18–31.68) (Supplementary Table 2, Supplementary Figures 2B,C). Regarding the national levels, from 1990 to 2004, rising trends were observed in 179 countries/territories, with the largest increase in Nepal (EAPC = 64.97, 95% CI 52.41–78.57), followed by Papua New Guinea and the Lao People's Democratic Republic. However, these trends decreased in only 13 countries/territories, and particularly in Burkina Faso (EAPC = −6.21, 95% CI −6.93–−5.50) (Figure 2B and Supplementary Table 7, Supplementary Figures 5B, 6B). Increasing trends in ASR of deaths occurred in most SDI areas and regions, except for high SDI areas (EAPC = −9.09, 95% CI −11.88–−6.20). The most pronounced increases were observed in Oceania and South Asia, in which the respective EAPCs were 44.76 (95% CI 40.81–48.82) and 40.82 (95% CI 34.31–47.64) (Supplementary Table 3, Supplementary Figures 3B,C). At the national level, decreasing trends were documented in 28 countries/territories from 1990 to 2004, and the largest declines were observed in New Zealand (EAPC = −12.99, 95% CI −14.98–−10.96), followed by France and Australia. Conversely, increasing trends were observed in 157 countries, particularly in Nepal (EAPC = 95.37, 95% CI 76.31–116.49), followed by the Lao People's Democratic Republic and Papua New Guinea (Figure 2C and Supplementary Table 8, Supplementary Figures 5C, 6C). Increasing trends of DALYs were observed in most SDI areas and regions, particularly in Oceania and South Asia, in which the EAPCs were 43.06 (95% CI 39.14–47.09) and 37.95 (95% CI 32.07–44.10), respectively. However, decreasing trends were observed in high SDI areas and in other regions, with the largest ones observed in Australasia (EAPC = −12.30, 95% CI −14.98–−9.53), followed by high-income North America and Western Europe (Supplementary Tables 4, 9, Supplementary Figures 4B,C).

**Figure 3:**
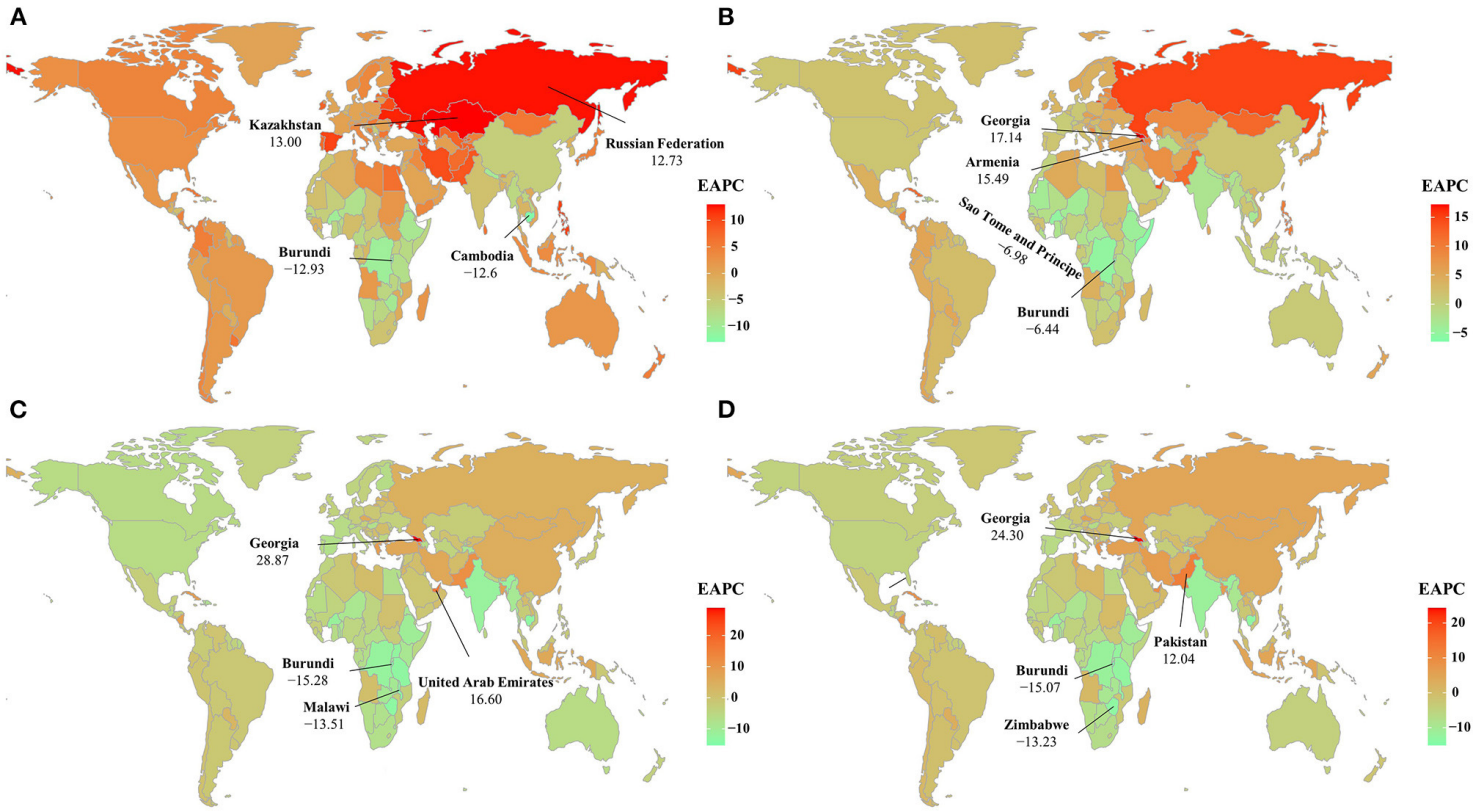
The distribution of the EAPCs of HIV/AIDS burden at the national level during the period 2005–2019, including incidence **(A)**, prevalence **(B)**, death **(C)**, and DALYs **(D)**. Countries/territories with extreme values are annotated. ASR, age-standardized rate; EAPC, estimated annual percentage change; DALYs, disability-adjusted life years.

During the period 2005–2019, decreasing trends in ASR of HIV/AIDS incidence were observed in low, low-middle, and middle SDI areas, particularly in low areas (EAPC = −5.29, 95% CI −5.61–−4.98). On the contrary, increasing trends were seen in high-middle and high SDI areas. At the regional level, the trends declined in eight geographic regions, and the largest declines were observed in Eastern Sub-Saharan Africa and East Asia, with EAPCs of −5.66 (95% CI −5.97–−5.35) and −5.35 (95% CI −6.31–−4.37). However, increasing trends occurred in 13 geographic regions, particularly in Eastern Europe (EAPC = 12.31, 95% CI 10.38–14.28) (Table 1 and Supplementary Figures 1B,C). Among the 204 countries/territories, the ASR presented downward trends in 72 countries, the largest being in Burundi (EAPC = −12.93, 95% CI −13.23–−12.63), followed by Cambodia and the Democratic Republic of the Congo. Conversely, the ASR in incidence showed increasing trends in 100 countries, particularly in Kazakhstan (EAPC = 13.00, 95% CI 11.52–14.50), followed by the Russian Federation and Ukraine (Figure 3A, and Supplementary Table 6, Supplementary Figures 7A, 8A). Trends in prevalence declined in low and low-middle SDI areas, but increased in other areas, particularly the high-middle SDI regions (EAPC = 4.42, 95% CI 3.78–5.06). An upward trend in prevalence occurred in most regions, especially in Eastern Europe and Central Asia, in which the EAPCs were 11.81 (95% CI 11.00–12.62) and 5.96 (95% CI 5.13–6.81), respectively (Supplementary Table 2 and Supplementary Figures 2B,C). Among the 204 countries/territories, decreasing trends in prevalence were observed in 44 countries from 2005 to 2019, particularly in Sao Tome and Principe (EAPC = −6.98, 95% CI −8.23–−5.71), followed by Burundi and Somalia. While rising trends occurred in 157 countries, the largest ones occurred in Georgia, Armenia, and the Russian Federation, with EAPCs of 17.14 (95% CI 14.11–20.25), 15.49 (95% CI 14.33–16.67), and 14.88 (95% CI 14.29–15.47), respectively (Figure 3B and Supplementary Table 7, Supplementary Figures 7B, 8B). The ASR of death showed a downward trend across all SDI areas and most regions, and the largest decreasing trends were observed in South Asia (EAPC = −11.20, 95% CI −12.03–−10.36), followed by Eastern Sub-Saharan Africa and Southern Sub-Saharan Africa. Conversely, increasing trends were observed in East Asia, Eastern Europe, and North Africa and the Middle East (Supplementary Table 3, Supplementary Figures 3B,C). At the national level, decreasing trends were observed in 128 countries/territories, particularly in Burundi, Malawi, and Zimbabwe, with respective EAPCs of −15.28 (95% CI −16.08–−14.47), −13.51 (95% CI −14.15–−12.87), and −13.34 (95% CI −14.31–−12.35). Conversely, increasing trends occurred in 45 countries/territories, and the most pronounced were in Georgia and the United Arab Emirates, in which the respective EAPCs were 28.87 (95% CI 18.33–40.35) and 16.60 (95% CI 14.66–18.57) (Figure 3C and Supplementary Table 8, Supplementary Figures 7C, 8C). Downward trends in DALYs were observed across all SDI areas and in most regions, and the largest decreasing trends were observed in South Asia and Eastern Sub-Saharan Africa, in which the EAPCs were −10.99 (95% CI −11.75–−10.22) and −9.45 (95% CI −9.69–−9.20), respectively (Supplementary Table 4, Supplementary Figures 4B,C). At the national level, decreasing trends were observed in 128 countries/territories, particularly in Burundi (EAPC = −15.07, 95% CI −15.79–−14.33). Conversely, increasing trends occurred in 46 countries/territories, and the largest ones were observed in Georgia (EAPC = 24.30, 95% CI 15.87–33.34), followed by Pakistan and Micronesia (Figure 3D and Supplementary Table 9, Supplementary Figures 7D, 8D).

### Factors Influencing the EAPC

During the period 1990–2004, the ASRs of HIV/AIDS incidence and prevalence of HIV/AIDS increased most rapidly among countries with the lowest baseline rates (ρ = −0.28, *p* < 0.001; ρ = −0.17, *p* = 0.018, respectively). Meanwhile, countries with lower HDI experienced a more rapid increase in ASR of HIV/AIDS prevalence, mortality, and DALYs (ρ = −0.25, *p* < 0.001; ρ = −0.49, *p* < 0.001; ρ = −0.51, *p* < 0.001, respectively).

**Figure 4:**
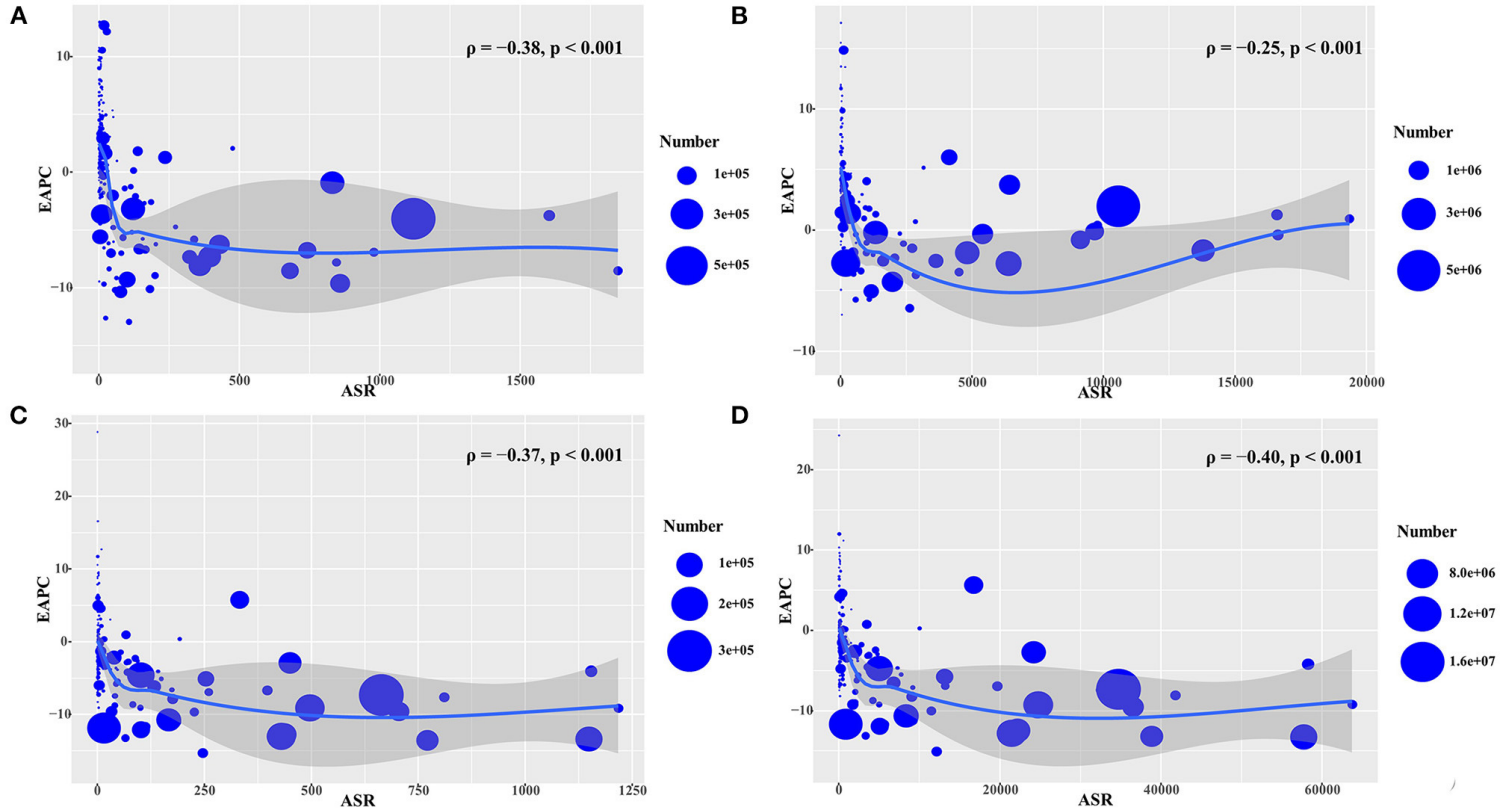
The association between EAPCs and ASR in 2005 at the national level during the period 2005–2019. The EAPCs of HIV/AIDS including the incidence **(A)**, prevalence **(B)**, death **(C)**, and DALYs **(D)** had a negative association with the corresponding ASR in 2005. The associations were calculated using Pearson correlation analysis. The circles represent countries that had available HDI data, and the size of circles changes according to the number of HIV/AIDS cases in the corresponding countries in 2005. ASR, age-standardized rate; EAPC, estimated annual percentage change; DALYs, disability-adjusted life years.

**Figure 5:**
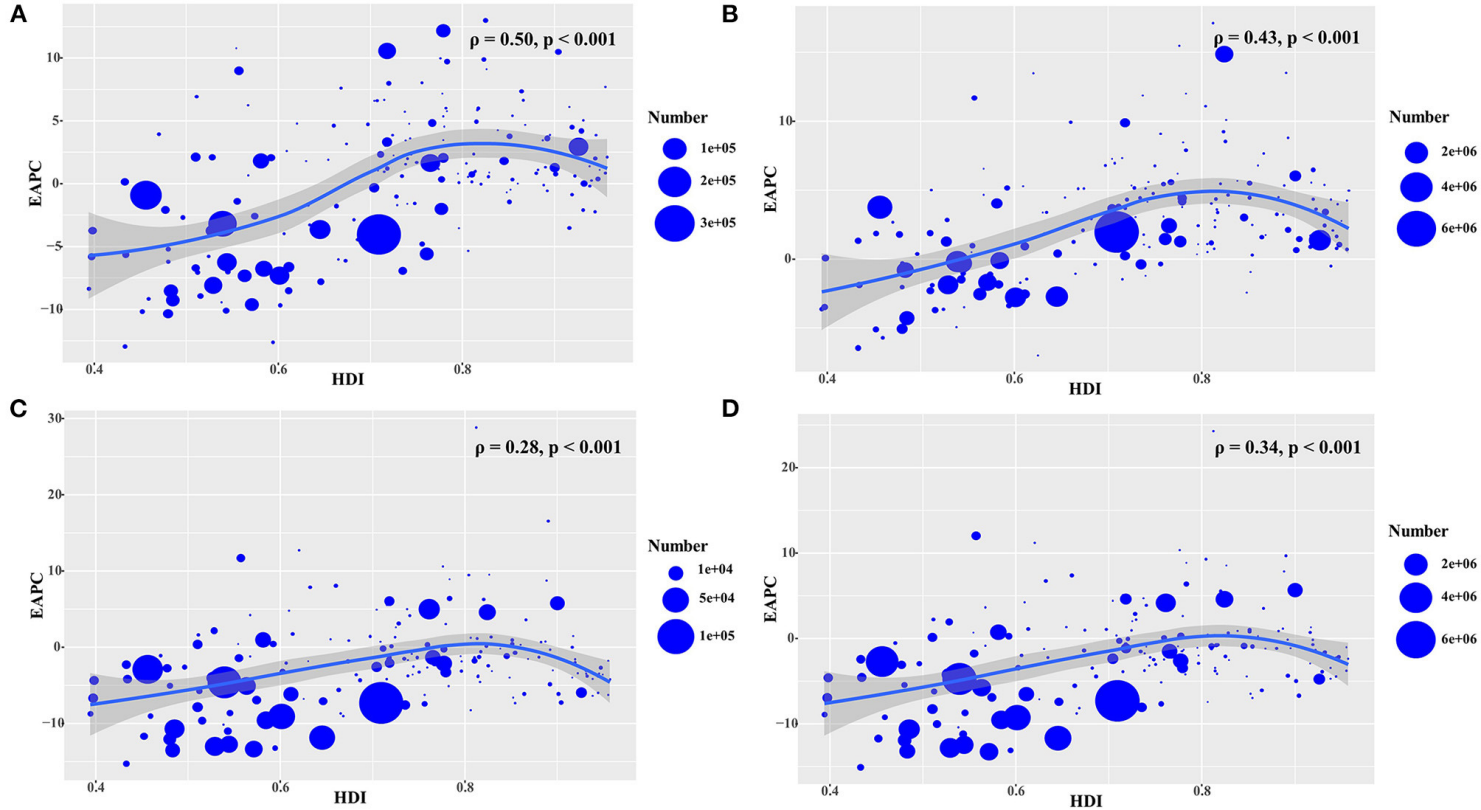
The association between EAPCs and HDI in 2019 at the national level during the period 2005–2019. The EAPCs of HIV/AIDS including the incidence **(A)**, prevalence **(B)**, death **(C)**, and DALYs **(D)** showed a positive association with the corresponding HDI in 2019. The associations were calculated using Pearson correlation analysis. The circles represent countries with available HDI data, and the size of the circles change according to the number of HIV/AIDS cases in the corresponding countries in 2019. EAPC, estimated annual percentage change; HDI, human development index.

During 2005–2019, we observed higher EAPCs in incidence, prevalence, deaths, and DALYs among the countries with lower ASRs (ρ = – 0.38, *p* < 0.001; ρ = −0.25, *p* < 0.001; ρ = −0.37, *p* < 0.001; and ρ = −0.40, *p* < 0.001, respectively) (Figures 4A–D). However, a more rapid increase in the ASR of prevalence, deaths, and DALYs of HIV/AIDS increased faster in countries with higher HDI (ρ = 0.50, *p* < 0.001; ρ = 0.43, *p* < 0.001; ρ = 0.28, *p* < 0.001; and ρ = 0.34, *p* < 0.001, respectively) (Figures 5A–D).

## Discussion

In the present study, the EAPC was first used as an index to describe the long-term temporal and spatial trends of HIV/AIDS from 1990 to 2019. The changes in the trends of HIV/AIDS disease burden were not only clarified, but the average annual rates of change in different indexes were also obtained. The key finding from this study is in the 15-year period, the incidence, mortality, and DALYs showed decreasing trends, except for the prevalence. This indicates that measures to control HIV/AIDS should focus on prevention to decrease the incidence and thus the disease burden of HIV/AIDS. The spatial trends indicated that, although the burden of HIV/AIDS continues to be concentrated in sub-Saharan Africa, it is encouraging to note that the trends have declined significantly over the past 15 years, while the incidence has shown a clear upward trend in high-income countries.

The temporal trends in the ASR of HIV/AIDS burden were found to present a parabolic curve during the period 1990–2019, and peaked around 2004, and with the lowest values in 1990 and 2019, respectively. Therefore, the year 2004 was selected as the time cutoff point to describe trends in two periods, specifically 1990–2004, and 2005–2019. Trends in ASR of HIV/AIDS burden, including prevalence, deaths, and DALYs, significantly increased during the period 1990–2004, but declined during 2005–2019, which objectively reflected the temporal trends of HIV/AIDS.

In 2004, the numbers of deaths and the incidence of HIV/AIDS were 1.84 million and 2.65 million, respectively. From 1990 to 2004, the numbers of deaths showed a stable upward trend, with an average annual rate of 10.85%. The incidence decreased at an average rate of 0.26% per year, but this trend was not stable. However, the combined effects of these trends resulted in a steady increase in the total number of people living with HIV, and its prevalence steadily increased at an average rate of approximately 7.47% per year. In the early 1990's, the HIV/AIDS epidemic was closely associated with poverty, poor healthcare infrastructure, lack of control policies, and the high prevalence of injection drug use, and sexual transmission (20–23). Decreasing trends in HIV/AIDS cases have been observed worldwide over the past 15 years (2005–2019). During this period, the incidence and the number of deaths decreased, resulting in stable downward trends, with an average annual rate of 2.68 and 6.73%, respectively, which might be due to the more effective prevention and control strategies as well as treatment interventions established in recent years, such as poverty reduction, improvement in health infrastructure, and intensive international cooperation (24). In 2000, HIV/AIDS was identified as one of the top health priorities and attracted considerable international funding by organizations (25). Furthermore, the WHO launched the Millennium Development Goals, which aimed to promote the availability of antiretroviral treatment in developing countries (26). International assistance in terms of financial and physical resources has continuously been given to support poor countries, with the main purpose of improving health and eliminating poverty (4). For example, by 2014, approximately 40% of all eligible HIV-infected individuals had received ART globally (27). In 2015, US$19 billion was invested in HIV/AIDS prevention in countries with low-income levels. The Chinese government had launched the program of free antiviral treatment since 2003 (28). With the introduction of the highly active treatment combination of ART, the mortality of HIV-infected patients dramatically declined.

In terms of spatial trends, during 1990–2004, increasing trends were mostly observed in the low, low-middle, and middle SDI areas, where there was a higher incidence of poverty, drug use, lower-level education, and weaker health systems (29). Among different geographic regions, Oceania, East Asia, and South Asia showed the largest increase in the number of deaths due to HIV/AIDS, which was probably due to the low availability of ART coverage (only 25.9%), far lower than that available globally (40.6%) and in low SDI countries (37.9%) (30). Pronounced increasing trends in HIV/AIDS cases were commonly observed in low-resource settings, where there were many obstacles to the control of HIV/AIDS transmission, including shortage of capital investment and poor healthcare infrastructure and education (31, 32), particularly in Nepal and the Lao People's Democratic Republic. In Nepal, high-risk sexual behaviors were common, including chance of sexual encounters, infrequent use of condoms, and substantially low knowledge of HIV disease (33). Meanwhile, a high HIV prevalence was reported among injection drug users (IDUs) (over 20%) (34). Among the seven Asia-Pacific countries, the Lao People's Democratic Republic had the lowest rate of inconsistent condom use among people living with HIV, which resulted in a higher risk of disease transmission (35). Furthermore, the situation was exacerbated by tuberculosis co-infection and drug resistance (36). However, decreasing trends in the number of HIV/AIDS cases were generally observed in developed regions and countries, such as New Zealand and Australia, where an early response to HIV/AIDS prevention among high-risk groups, led to a high-level of awareness, and was accompanied by robust healthcare systems (37–39).

During the second period (2005–2019), the areas with the fastest declines in incidence, prevalence, deaths, and DALYs were all in lower SDI level areas, which partly reflected the great progress achieved in the prevention and treatment of HIV/AIDS by government efforts and multilateral organizations toward low and low-middle SDI areas (9, 12, 40, 41). The most pronounced decreasing trends were found in sub-Saharan African countries, including Burundi, Malawi, and Zimbabwe, probably due to the improved health system, secondary education, and the practice of medical circumcision in adolescents and children (42–44). Meanwhile, an increase in medical investments promoted an improvement in the prevention and control of HIV in sub-Sahara African countries (45). In Burundi and Malawi, the “treat all” policies effectively facilitated the initiation of rapid ART from 2004 to 2018 (46). Furthermore, reducing HIV mother-to-child transmission successfully reduced the number of new pediatric HIV infections (47). However, increasing trends in HIV/AIDS cases occurred in Eastern Europe and Central Asia, which were due to the underdeveloped economy, faulty healthcare system, and high-risk behaviors (48, 49). In the former Soviet countries, tuberculosis, drug use, and HIV infection were major public problems (50–52), which probably generated the pronounced increasing trends observed in Georgia, Armenia, and Kazakhstan. For example, a high prevalence of HIV was reported among IDU (>30%) in Kazakhstan (53). Furthermore, 35.8% of people living with HIV did not report good health-related quality of life, and the adverse factors included depression, coinfection with tuberculosis, and sexually transmitted diseases (54).

The ASR of HIV/AIDS in 2005 reflects the disease reservoir at baseline. We found that the HIV/AIDS was more likely to increase in countries with a low ASR in 2005. This result might be explained by the following: (1) the changes in ASR are more pronounced in countries with low ASR baselines; (2) countries with low ASR are less likely than other public health problems to consider HIV/AIDS infection a priority in disease prevention plans; and (3) in countries with high ASR, there is more support from WHO for AIDS prevention and treatment.

Despite the considerable progress achieved in the prevention and control of HIV/AIDS, there is still neither a cure nor an effective and safe vaccine available for it (55), and the emergence of anti-HIV drug resistance has brought substantial challenges (56, 57). In addition, the above problems indicate that greater preventive measures should be taken to expand coverage in order to reduce the incidence of HIV/AIDS, thereby accelerating the rate of decline of the incidence and prevalence.

Several limitations should be considered in this study: (1) estimates of disease burden in the GBD studies were based on the quality and quantity of data, as well as on the potential for misdiagnosis and/or miscoding of diseases in different countries, including unreported cases, poor test technology, and incomplete reporting, which might have affected the accuracy and robustness of the results; (2) the advancement in diagnostic techniques of HIV/AIDS also varied between countries and over time, which may have generated potential biases; (3) due to the limitations of the ASR formula, although age was an important factor, only percentage changes in the number of events across age groups were used in this study to estimate trends; and (4) the EAPC may not apply to the entire time period of interest if the trend is not constant, because the ratio is linear on a log scale, meaning that the rate of change is constant. Thus, we selected the year 2004, which was the transition point of the trend in HIV/AIDS disease burden, as the time cutoff point to describe trends in the two periods.

## Conclusion

We first used EAPC of different indexes to analyze the disease burden of HIV/AIDS in terms of temporal and spatial trends, to more accurately reflect the changes. Meanwhile, we got the average annual rates of change of different measuring indexes in HIV/AIDS disease burden. Trends in HIV/AIDS burden dramatically increased during the period 1990–2004, but decreased from 2005 to 2019, particularly in sub-Saharan Africa, where while the burden of the global HIV epidemic continues to be concentrated, it is encouraging to note that morbidity, mortality and DALYs have declined significantly over the past 15 years. However, the prevalence of HIV/AIDS remained upward over the past decade worldwide. A concerning upward trend of incidence in High-middle and High SDI areas appeared. The results indicated that considerable progress had made in addressing the HIV-related burden worldwide, particularly in the high-risk settings. More than other interventions, prevention, and control strategies to incidence are needed to reduce the burden of HIV/AIDS, thereby achieving the goal set for 2030 by UNAIDS.

## Author's Note

All named authors meet the International Committee of Medical Journal Editors (ICMJE) criteria for authorship for this article, take responsibility for the integrity of the work as a whole, and have given their approval for this version to be published.

## Data Availability Statement

Publicly available datasets were analyzed in this study. This data can be found here: http://ghdx.healthdata.org/gbd-results-tool/.

## Author Contributions

HH and ZO: project administration and drafting. DY and YLia: data analysis and validation. WH and YG: data analysis and visualization. YLi and FW: data collection and collation. QC: supervision and drafting and editing. All authors contributed to the article and approved the submitted version.

## Conflict of Interest

The authors declare that the research was conducted in the absence of any commercial or financial relationships that could be construed as a potential conflict of interest.

## Publisher's Note

All claims expressed in this article are solely those of the authors and do not necessarily represent those of their affiliated organizations, or those of the publisher, the editors and the reviewers. Any product that may be evaluated in this article, or claim that may be made by its manufacturer, is not guaranteed or endorsed by the publisher.

## References

[1] Gunthard HF Saag MS Benson CA del Rio C Eron JJ Gallant JE. Antiretroviral Drugs for Treatment and Prevention of HIV Infection in Adults: 2016 Recommendations of the International Antiviral Society-USA Panel. JAMA. (2016) 316:191–10. 10.1001/jama.2016.8900 27404187 PMC5012643

[2] Gunthard HF Aberg JA Eron JJ Hoy JF Telenti A Benson CA. Antiretroviral treatment of adult HIV infection: 2014 recommendations of the International Antiviral Society-USA Panel. JAMA. (2014) 312:410–25. 10.1001/jama.2014.8722 25038359

[3] Vellozzi C Brooks JT Bush TJ Conley LJ Henry K Carpenter CC. The study to understand the natural history of HIV and AIDS in the era of effective therapy (SUN Study). Am J Epidemiol. (2009) 169:642–52. 10.1093/aje/kwn361 19074775

[4] Global Burden of Disease Health Financing Collaborator N. Spending on health and HIV/AIDS: domestic health spending and development assistance in 188 countries, 1995-2015. Lancet. (2018) 391:1799–829. 10.1016/S0140-6736(18)30698-6 29678342 PMC5946845

[5] Granich R Williams B Montaner J Zuniga JM. 90-90-90 and ending AIDS: necessary and feasible. Lancet. (2017) 390:341–3. 10.1016/S0140-6736(17)31872-X 28745591

[6] Anne Crock E. HIV and AIDS: an overview of the current issues, treatment and prevention. Nurs Stand. (2017) 32:51–63. 10.7748/ns.2017.e11045 29210536

[7] Strathdee SA Hallett TB Bobrova N Rhodes T Booth R Abdool R. HIV and risk environment for injecting drug users: the past, present, and future. Lancet. (2010) 376:268–84. 10.1016/S0140-6736(10)60743-X 20650523 PMC6464374

[8] Krishnaratne S Hensen B Cordes J Enstone J Hargreaves JR. Interventions to strengthen the HIV prevention cascade: a systematic review of reviews. Lancet HIV. (2016) 3:e307–17. 10.1016/S2352-3018(16)30038-8 27365205

[9] Kharsany AB Karim QA. HIV infection and AIDS in Sub-Saharan Africa: current status, challenges and opportunities. Open AIDS J. (2016) 10:34–48. 10.2174/1874613601610010034 27347270 PMC4893541

[10] Stover J Bollinger L Izazola JA Loures L DeLay P Ghys PD. What is required to end the AIDS epidemic as a public health threat by 2030? The cost and impact of the fast-track approach. PloS ONE. (2019) 14:e0213970. 10.1371/journal.pone.0213970. 30870508 PMC6417641

[11] Saag MS Benson CA Gandhi RT Hoy JF Landovitz RJ Mugavero MJ. Antiretroviral Drugs for Treatment and Prevention of HIV Infection in Adults: 2018 Recommendations of the International Antiviral Society-USA Panel. JAMA. (2018) 320:379–96. 10.1001/jama.2018.8431 30043070 PMC6415748

[12] Stover J Fidzani B Molomo BC Moeti T Musuka G. Estimated HIV trends and program effects in Botswana. PLoS ONE. (2008) 3:e3729. 10.1371/journal.pone.0003729 19008957 PMC2579326

[13] Collaborators GH. Global, regional, and national incidence, prevalence, and mortality of HIV, 1980-2017, and forecasts to 2030, for 195 countries and territories: a systematic analysis for the Global Burden of Diseases, Injuries, and Risk Factors Study 2017. Lancet HIV. (2019) 6:e831–e59. 10.1016/S2352-3018(19)30196-1 31439534 PMC6934077

[14] Govender RD Hashim MJ Khan MA Mustafa H Khan G. Global epidemiology of HIV/AIDS: a resurgence in North America and Europe. J Epidemiol Glob Health. (2021) 11:296–301. 10.2991/jegh.k.210621.001 34270183 PMC8435868

[15] Fay MP Tiwari RC Feuer EJ Zou Z. Estimating average annual percent change for disease rates without assuming constant change. Biometrics. (2006) 62:847–54. 10.1111/j.1541-0420.2006.00528.x 16984328

[16] Clegg LX Hankey BF Tiwari R Feuer EJ Edwards BK. Estimating average annual per cent change in trend analysis. Stat Med. (2009) 28:3670–82. 10.1002/sim.3733 19856324 PMC2843083

[17] Zhang R Li F. Comparison of the application of annual estimated percentage change and average growth rate in public health. Chin J Health Stat. (2015) 32:328–9+32.

[18] Hankey BF Ries LA Kosary CL Feuer EJ Merrill RM Clegg LX. Partitioning linear trends in age-adjusted rates. Cancer Causes Control. (2000) 11:31–5. 10.1023/A:1008953201688 10680727

[19] Liu Z Jiang Y Yuan H Fang Q Cai N Suo C. The trends in incidence of primary liver cancer caused by specific etiologies: results from the global burden of disease study 2016 and implications for liver cancer prevention. J Hepatol. (2019) 70:674–83. 10.1016/j.jhep.2018.12.001 30543829

[20] DeHovitz J Uuskula A El-Bassel N. The HIV epidemic in Eastern Europe and Central Asia. Curr HIV/AIDS Rep. (2014) 11:168–76. 10.1007/s11904-014-0202-3 24652411

[21] Platt L Jolley E Rhodes T Hope V Latypov A Reynolds L. Factors mediating HIV risk among female sex workers in Europe: a systematic review and ecological analysis. Bmj Open. (2013) 3:e002836. 10.1136/bmjopen-2013-002836 23883879 PMC3731729

[22] Gouws E Cuchi P. I ICEH. Focusing the HIV response through estimating the major modes of HIV transmission: a multi-country analysis. Sex Transm Infect. (2012) 88:I76–85. 10.1136/sextrans-2012-050719 23172348 PMC3512398

[23] Vickerman P Platt L Jolley E Rhodes T Kazatchkine MD Latypov A. Controlling HIV among people who inject drugs in Eastern Europe and Central Asia: insights from modeling. Int J Drug Policy. (2014) 25:1163–73. 10.1016/j.drugpo.2014.09.013 25449056

[24] Schneider MT Birger M Haakenstad A Singh L Hamavid H Chapin A. Tracking development assistance for HIV/AIDS: the international response to a global epidemic. AIDS. (2016) 30:1475–9. 10.1097/QAD.0000000000001081 26950317 PMC4867985

[25] Ravishankar N Gubbins P Cooley RJ Leach-Kemon K Michaud CM Jamison DT. Financing of global health: tracking development assistance for health from 1990 to 2007. Lancet. (2009) 373:2113–24. 10.1016/S0140-6736(09)60881-3 19541038

[26] Prendergast AJ Essajee S Penazzato M. HIV and the Millennium Development Goals. Arch Dis Child. (2015) 100:S48–52. 10.1136/archdischild-2013-305548 25613968

[27] Wandeler G Johnson LF Egger M. Trends in life expectancy of HIV-positive adults on antiretroviral therapy across the globe: comparisons with general population. Curr Opin HIV AIDS. (2016) 11:492–500. 10.1097/COH.0000000000000298 27254748 PMC5055447

[28] Chen H Liu P Ruan Y. Progress of China′s National Free AIDS Antiretroviral Treatment Program and “Treatment as Prevention. China Trop Med. (2019) 19:1194–6. 10.13604/j.cnki.46-1064/r.2019.12.23

[29] Dutta A Wirtz AL Baral S Beyrer C Cleghorn FR. Key harm reduction interventions and their impact on the reduction of risky behavior and HIV incidence among people who inject drugs in low-income and middle-income countries. Curr Opin Hiv Aids. (2012) 7:362–8. 10.1097/COH.0b013e328354a0b5 22647588

[30] Wang HD Wolock TM Carter A Nguyen G Kyu HH Gakidou E. Estimates of global, regional, and national incidence, prevalence, and mortality of HIV, 1980-2015: the Global Burden of Disease Study 2015. Lancet HIV. (2016) 3:E361–E87. 10.1016/S2352-3018(16)30087-X 27470028 PMC5056319

[31] Poudel AN Newlands D Simkhada P. The economic burden of HIV/AIDS on individuals and households in Nepal: a quantitative study. BMC Health Serv Res. (2017) 17:76. 10.1186/s12913-017-1976-y 28118830 PMC5259845

[32] Duke T. HIV in Papua New Guinea: the need for practical action, and a focus on human resources and health systems for women and children. J Paediatr Child Health. (2008) 44:611–2. 10.1111/j.1440-1754.2008.01396.x 19012640

[33] Poudel KC Jimba M Okumura J Joshi AB Wakai S. Migrants' risky sexual behaviours in India and at home in far western Nepal. Trop Med Int Health. (2004) 9:897–903. 10.1111/j.1365-3156.2004.01276.x 15303995

[34] Aceijas C Stimson GV Hickman M Rhodes T. HIV UNRG. Global overview of injecting drug use and HIV infection among injecting drug users. AIDS. (2004) 18:2295–303. 10.1097/00002030-200411190-00010 15577542

[35] Deuba K Kohlbrenner V Koirala S Ekstrom AM. group C-S. Condom use behaviour among people living with HIV: a seven-country community-based participatory research in the Asia-Pacific region. Sex Transm Infect. (2018) 94:200–5. 10.1136/sextrans-2017-053263 29118203 PMC5969330

[36] Zhang J Kern-Allely S Yu T Price RK. HIV and tuberculosis co-infection in East Asia and the Pacific from 1990 to 2017: results from the Global Burden of Disease Study 2017. J Thorac Dis. (2019) 11:3822–35. 10.21037/jtd.2019.09.23 31656655 PMC6790465

[37] Hughes AJ Saxton PJ. Thirty years of condom-based HIV prevention by gay men in New Zealand. N Z Med J. (2015) 128:19–30. 26913905

[38] Worth H Denholm N Bannister J. HIV/AIDS and the African Refugee Education Program in New Zealand. AIDS Educ Prev. (2003) 15:346–56. 10.1521/aeap.15.5.346.23819 14516019

[39] Sendziuk P. Harm reduction and HIV-prevention among injecting drug users in Australia: an international comparison. Can Bull Med Hist. (2007) 24:113–29. 10.3138/cbmh.24.1.113 17644934

[40] Westercamp N Bailey RC. Acceptability of male circumcision for prevention of HIV/AIDS in sub-Saharan Africa: a review. AIDS Behav. (2007) 11:341–55. 10.1007/s10461-006-9169-4 17053855 PMC1847541

[41] Balasundaram A Sarkar S Hamide A Lakshminarayanan S. Socioepidemiologic profile and treatment-seeking behaviour of HIV/AIDS patients in a tertiary-care hospital in south India. J Health Popul Nutr. (2014) 32:587–94. 25895191 PMC4438688

[42] Halperin DT Mugurungi O Hallett TB Muchini B Campbell B Magure T. A surprising prevention success: why did the HIV epidemic decline in Zimbabwe? PLoS Med. (2011) 8:e1000414. 10.1371/journal.pmed.1000414 21346807 PMC3035617

[43] Barankanira E Molinari N Niyongabo T Laurent C. Spatial analysis of HIV infection and associated individual characteristics in Burundi: indications for effective prevention. BMC Public Health. (2016) 16:118. 10.1186/s12889-016-2760-3 26847711 PMC4743168

[44] Jaganath D Mulenga C Hoffman RM Hamilton J Boneh G. This is My Story: participatory performance for HIV and AIDS education at the University of Malawi. Health Educ Res. (2014) 29:554–65. 10.1093/her/cyt074 24047713 PMC4155417

[45] Bein M Coker-Farrell EY. The association between medical spending and health status: a study of selected African countries. Malawi Med J. (2020) 32:37–44. 10.4314/mmj.v32i1.8 32733658 PMC7366161

[46] Tymejczyk O Brazier E Yiannoutsos CT Vinikoor M van Lettow M Nalugoda F. Changes in rapid HIV treatment initiation after national ”treat all“ policy adoption in 6 sub-Saharan African countries: Regression discontinuity analysis. PLoS Med. (2019) 16:e1002822. 10.1371/journal.pmed.1002822 31181056 PMC6557472

[47] Omonaiye O Kusljic S Nicholson P Manias E. Factors Associated With Success in Reducing HIV Mother-to-child Transmission in Sub-Saharan Africa: Interviews With Key Stakeholders. Clin Therap. (2019) 41:2102–10 e1. 10.1016/j.clinthera.2019.08.012 31522825

[48] Chkhartishvili N Sharvadze L Chokoshvili O Bolokadze N Rukhadze N Kempker RR. Mortality and causes of death among HIV-infected individuals in the country of Georgia: 1989-2012. AIDS Res Hum Retroviruses. (2014) 30:560–6. 10.1089/aid.2013.0219 24472093 PMC4046195

[49] Altice FL Azbel L Stone J Brooks-Pollock E Smyrnov P Dvoriak S. The perfect storm: incarceration and the high-risk environment perpetuating transmission of HIV, hepatitis C virus, and tuberculosis in Eastern Europe and Central Asia. Lancet. (2016) 388:1228–48. 10.1016/S0140-6736(16)30856-X 27427455 PMC5087988

[50] Schluger NW El-Bassel N Hermosilla S Terlikbayeva A Darisheva M Aifah A. Tuberculosis, drug use and HIV infection in Central Asia: an urgent need for attention. Drug Alcohol Depend. (2013) 132 Suppl 1:S32–6. 10.1016/j.drugalcdep.2013.07.012 23928052

[51] Shaw SA Terlikbayeva A Famouri L Hunt T Gilbert L Rozental Y. HIV testing and access to HIV medical care among people who inject drugs and their intimate partners in Kazakhstan. J Subst Use. (2017) 22:53–9. 10.3109/14659891.2016.1143046 30220879 PMC6138444

[52] Kalichman SC Cherry C Amaral C White D Kalichman MO Pope H. Health and treatment implications of food insufficiency among people living with HIV/AIDS, Atlanta, Georgia. J Urban Health. (2010) 87:631–41. 10.1007/s11524-010-9446-4 20419478 PMC2900577

[53] Davlidova S Haley-Johnson Z Nyhan K Farooq A Vermund SH Ali S. Prevalence of HIV, HCV and HBV in Central Asia and the Caucasus: a systematic review. Int J Infect Dis. (2021) 104:510–25. 10.1016/j.ijid.2020.12.068 33385583 PMC11094609

[54] Zhakipbayeva BT Nugmanova ZS Tracy M Birkhead GS Akhmetova GM DeHovitz J. Factors influencing the quality of life in persons living with human immunodeficiency virus infection in Almaty, Kazakhstan. Int J STD AIDS. (2019) 30:1318–28. 10.1177/0956462419876484 31726932 PMC7433689

[55] Alchin DR. HIV vaccine development: an exploratory review of the trials and tribulations. Immunol Res. (2014) 60:35–7. 10.1007/s12026-014-8551-y 24847767

[56] Liu Z Wang Y Yedidi RS Dewdney TG Reiter SJ Brunzelle JS. Conserved hydrogen bonds and water molecules in MDR HIV-1 protease substrate complexes. Biochem Biophys Res Commun. (2013) 430:1022–7. 10.1016/j.bbrc.2012.12.045 23261453 PMC4520401

[57] Mansky LM. HIV mutagenesis and the evolution of antiretroviral drug resistance. Drug Resist Updat. (2002) 5:219–23. 10.1016/S1368-7646(02)00118-8 12531178

