# Supplementary material for: Spatial and Temporal Trends in HIV/AIDS Burden Among Worldwide Regions From 1990 to 2019: A Secondary Analysis of the Global Burden of Disease Study 2019
Source: Front Med (Lausanne). 2022 May 12;9:808318. doi: 10.3389/fmed.2022.808318 (PMC9133478; doi:10.3389/fmed.2022.808318)
Supplement: Supplementary file 1 [file Data_Sheet_1.docx]

**Supplementary figure 1**. The distribution of the incident number of HIV/AIDS cases according to age groups, SDI areas, and geographic regions from 1990 to 2019. (A) The number of incident HIV/AIDS cases stratified by age groups; (B) according to SDI areas; and (C) across geographical regions.

**
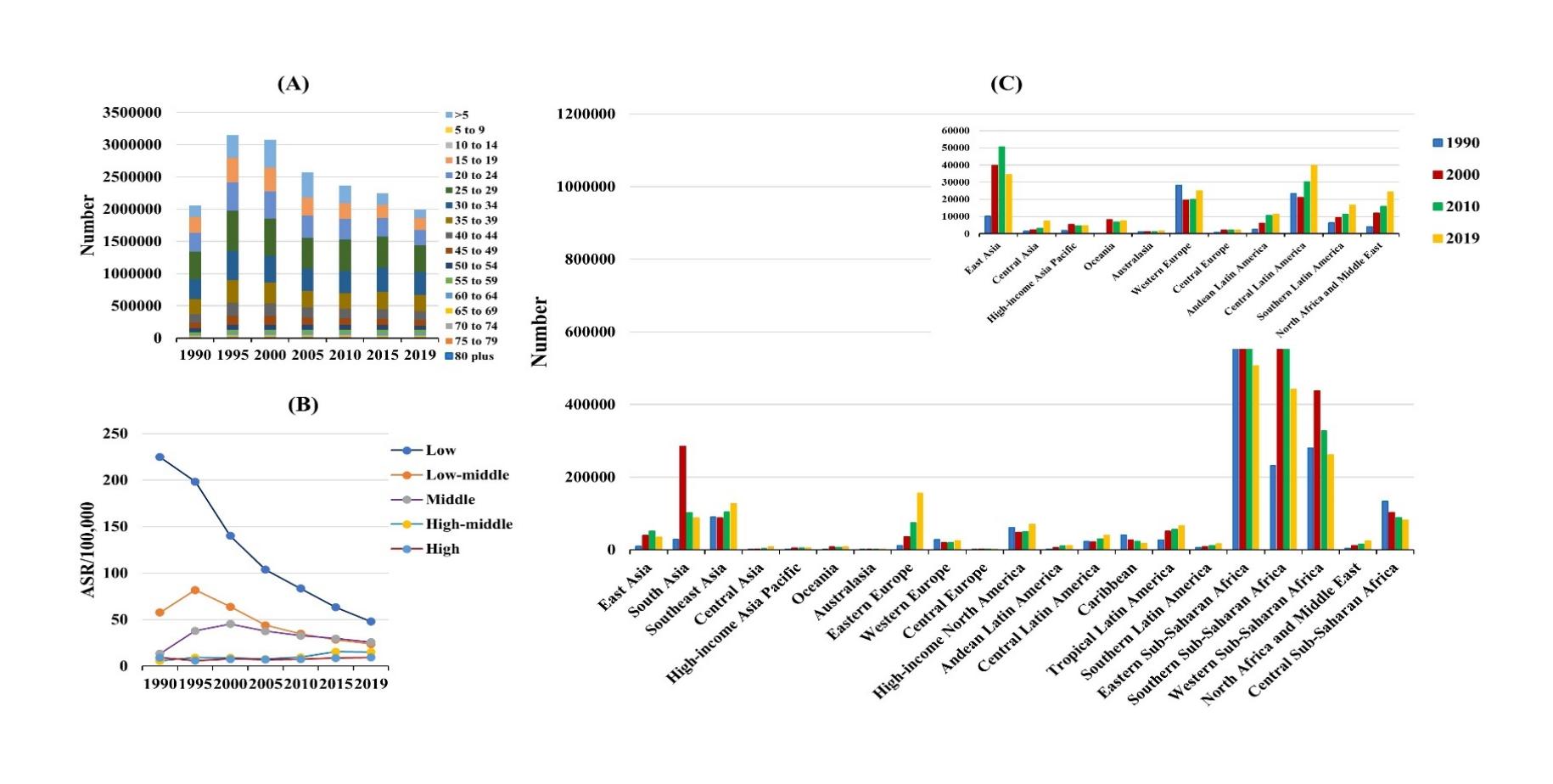
**

**Supplementary figure 2.** The distribution of the HIV/AIDS prevalence according to age groups, SDI areas, and geographic regions from 1990 to 2019. (A) The prevalence of HIV/AIDS cases stratified by age groups; (B) according to SDI areas; and (C) across geographical regions.

**
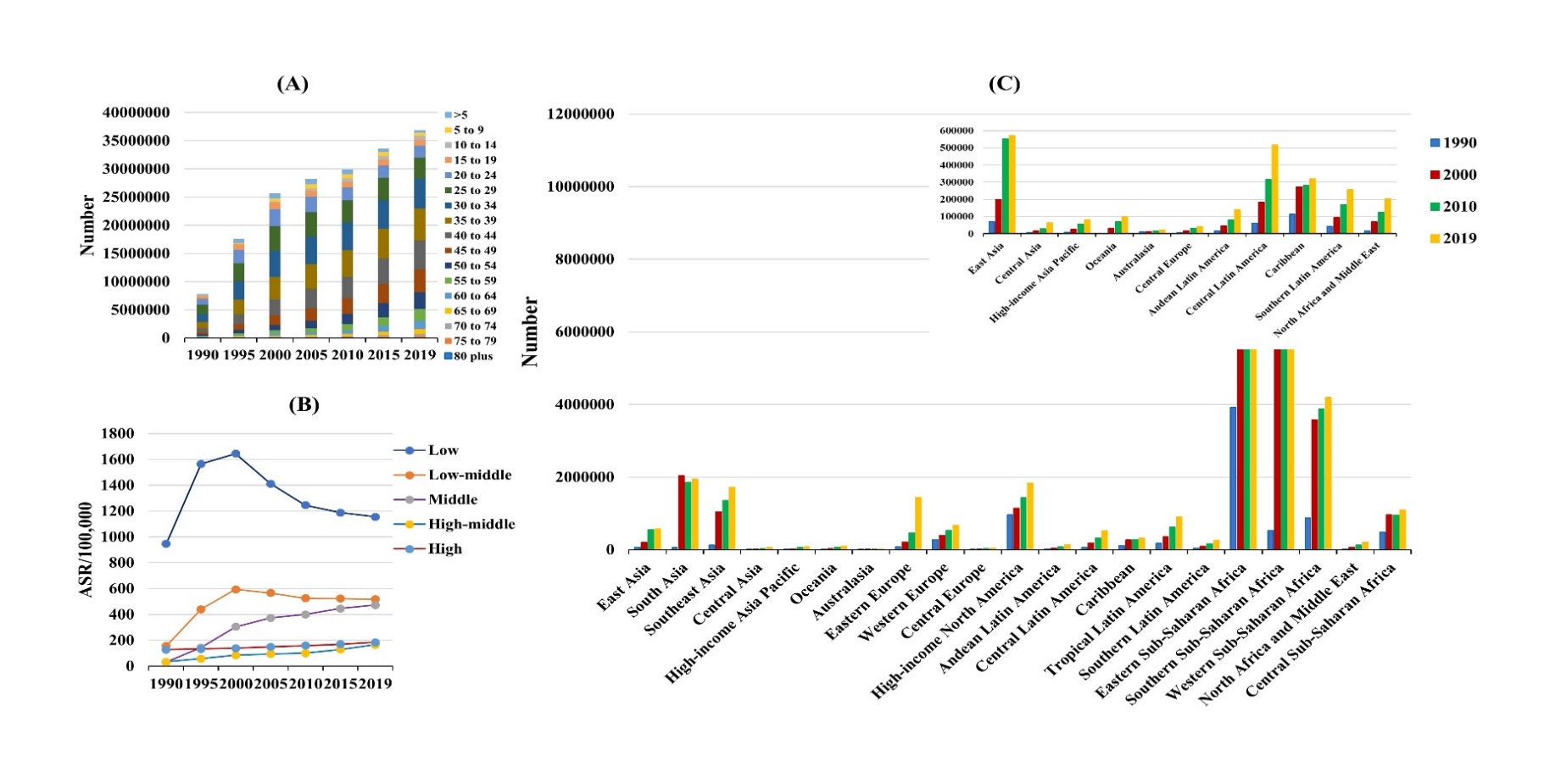
**

**Supplementary figure 3**. The distribution of the number of deaths due to HIV/AIDS according to age groups, SDI areas, and geographic regions from 1990 to 2019. (A) The number of deaths due to HIV/AIDS stratified by age groups; (B) according to SDI areas; and (C) across geographical regions.

**
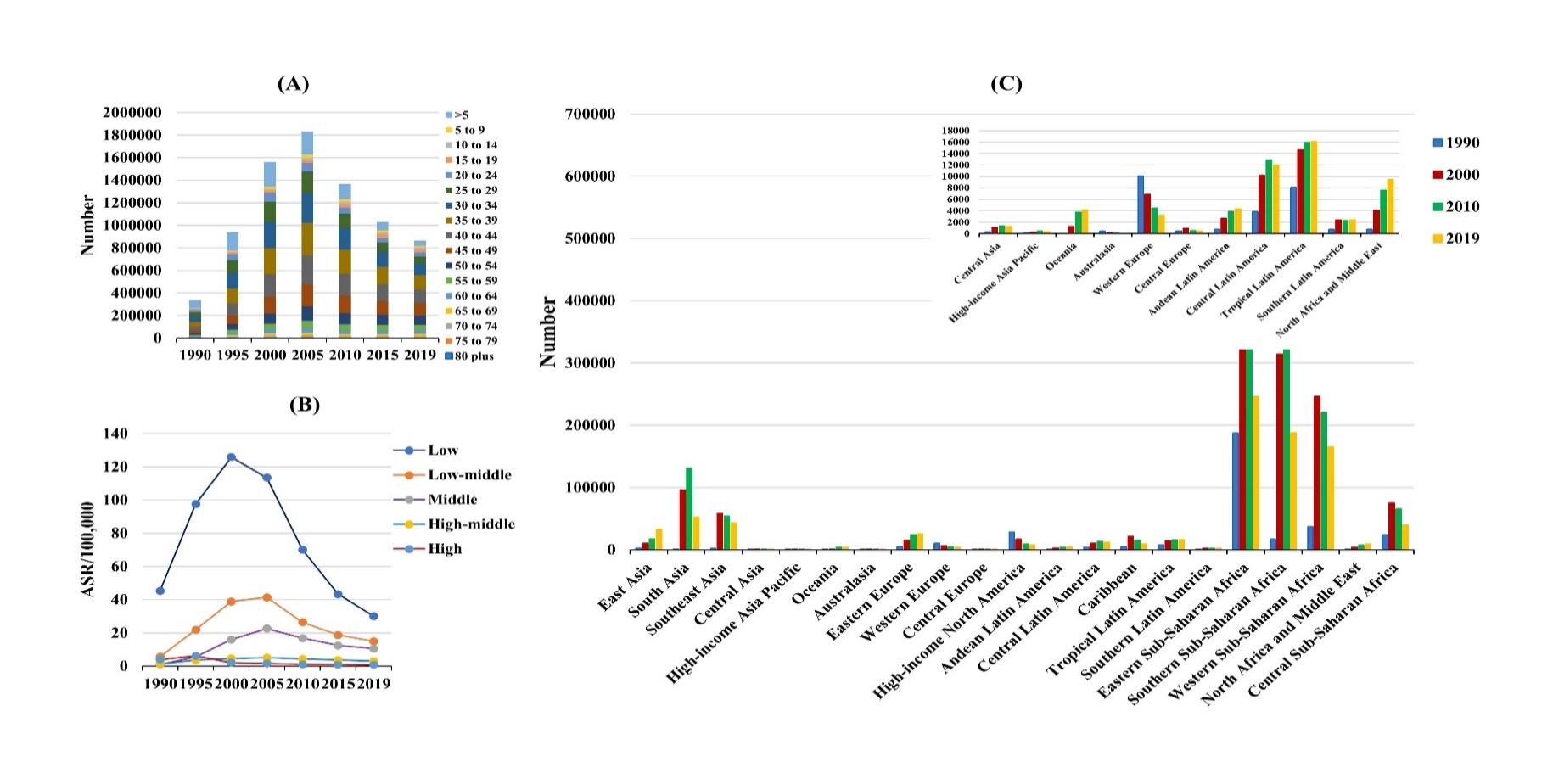
**

**Supplementary figure 4**. The distribution of the DALYs caused by HIV/AIDS according to age groups, SDI areas, and geographic regions from 1990 to 2019. (A) The distribution of the DALYs caused by HIV/AIDS stratified by age groups; (B) according to SDI areas; and (C) across geographical regions.

**
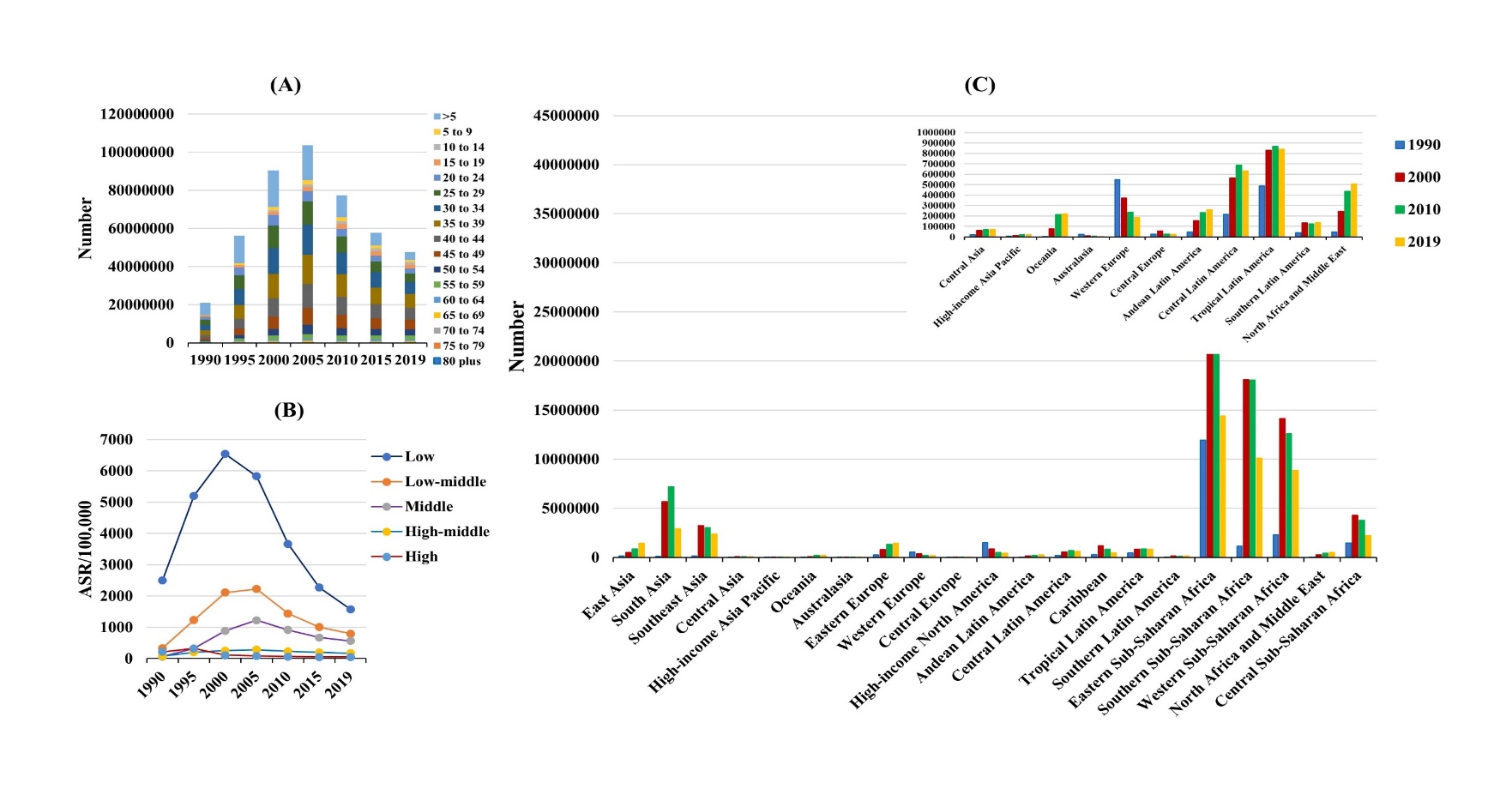
**

**Supplementary figure 5.** The distribution of the ASR of HIV/AIDS at the national level in 2004, including incidence (A), prevalence (B), deaths (C), and DALYs (D). Countries/territories with an extreme value were annotated. ASR, age-standardized rate; EAPC, estimated annual percentage change.

**
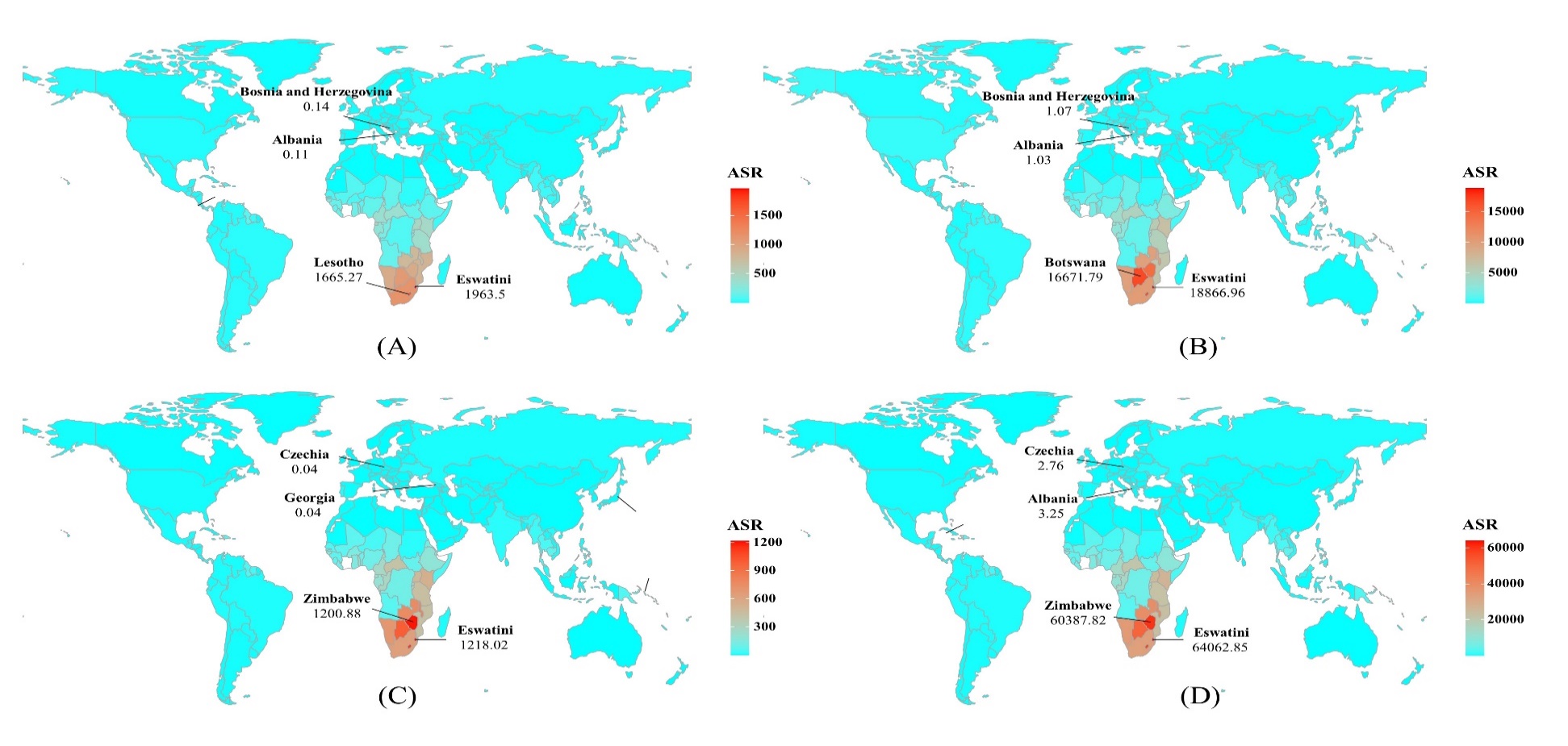
**

**Supplementary figure 6**. The distribution of percentage changes in the number of HIV/AIDS cases at the national level during the period 1990 – 2004, including incidence (A), prevalence (B), deaths (C), and DALYs (D). Countries/territories with an extreme value were annotated.

**
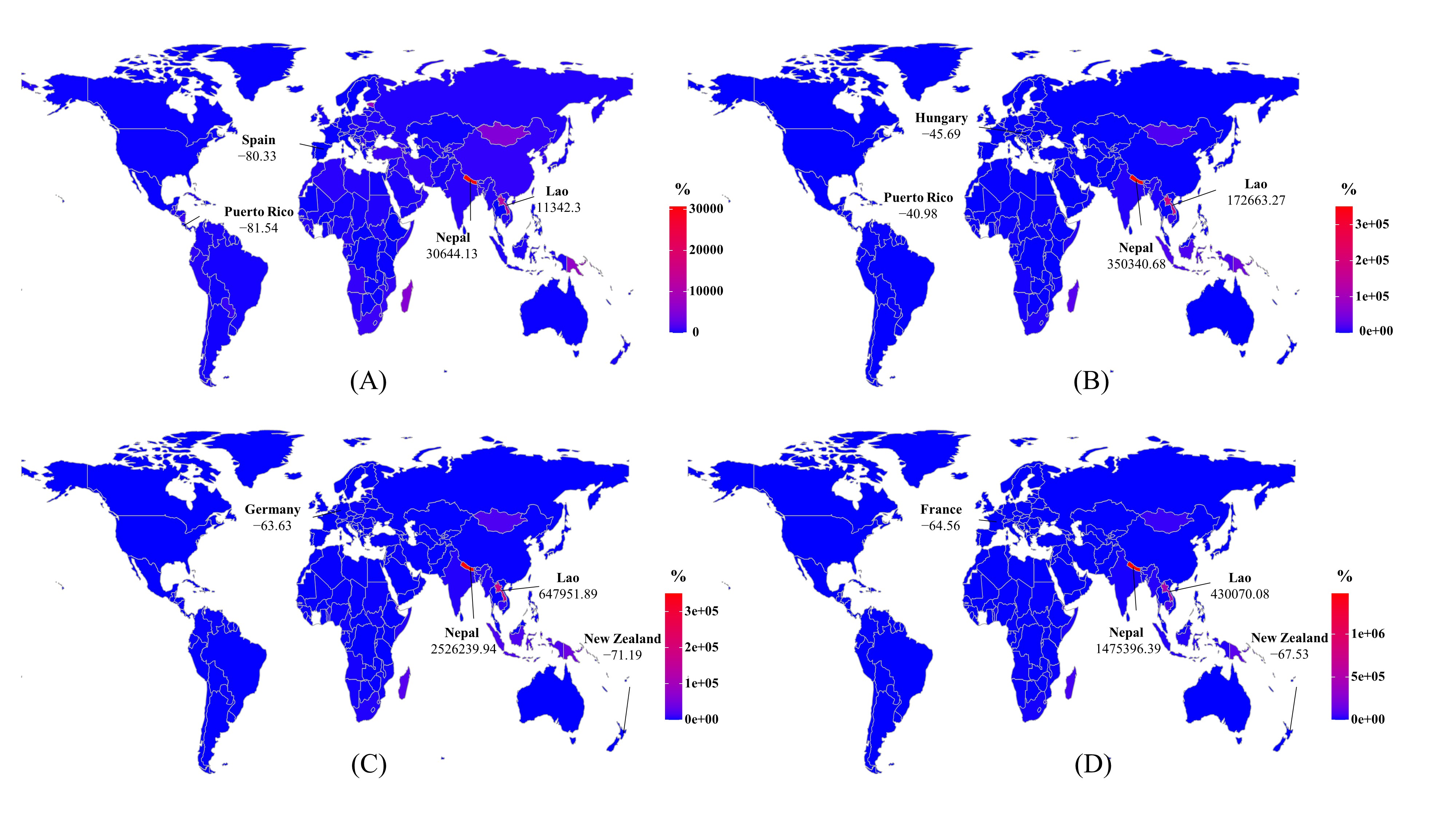
**

**Supplementary figure 7.** The distribution of the ASR of HIV/AIDS at the national level in 2019, including incidence (A), prevalence (B), deaths (C), and DALYs (D). Countries/territories with an extreme value were annotated. ASR, age-standardized rate; EAPC, estimated annual percentage change.

**
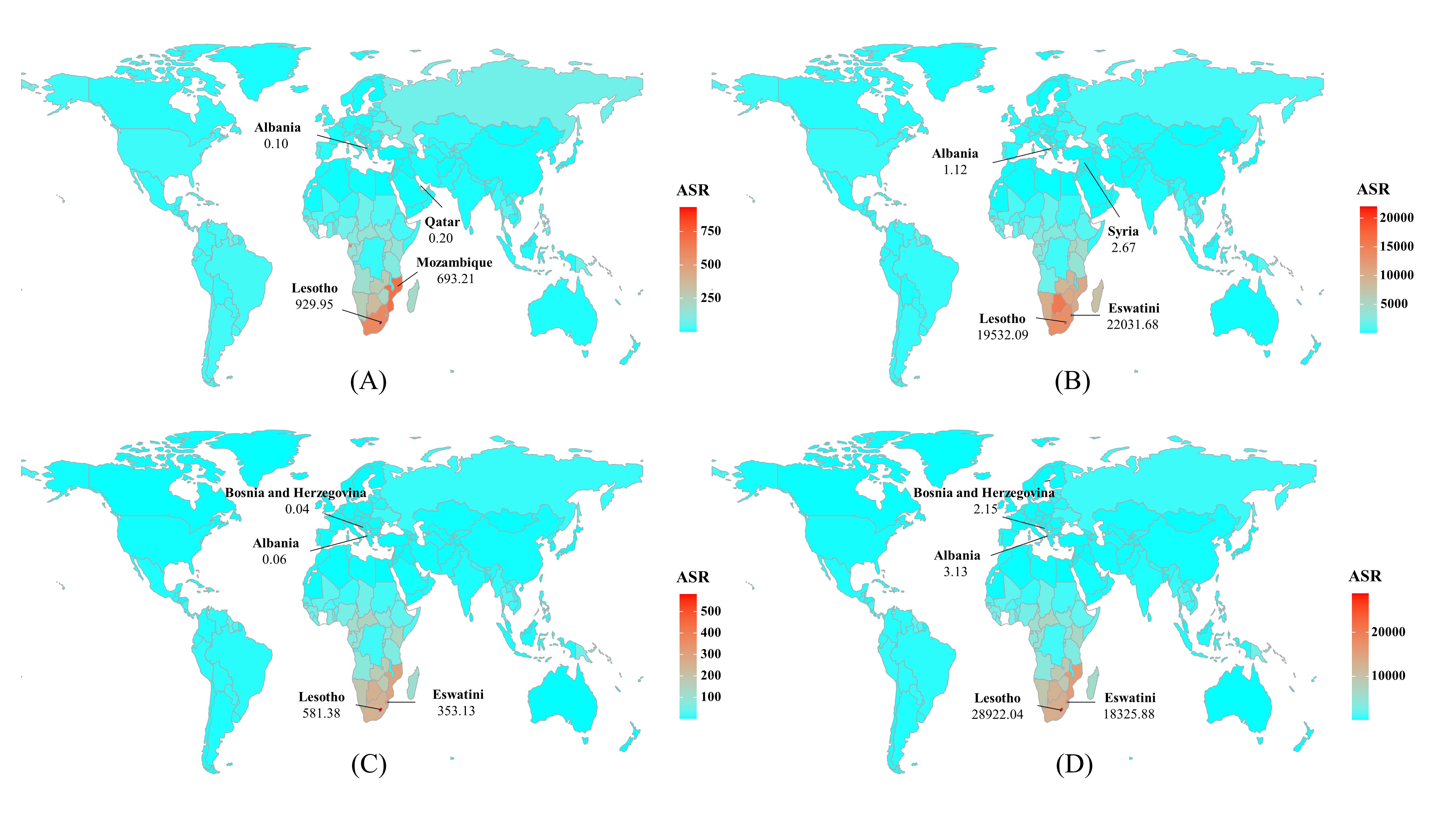
**

**Supplementary figure 8**. The distribution of percentage changes in the number of HIV/AIDS at a national level during the period 2005 – 2019, including incidence (A), prevalence (B), deaths (C), and DALYs (D). Countries/territories with an extreme value were annotated.

**
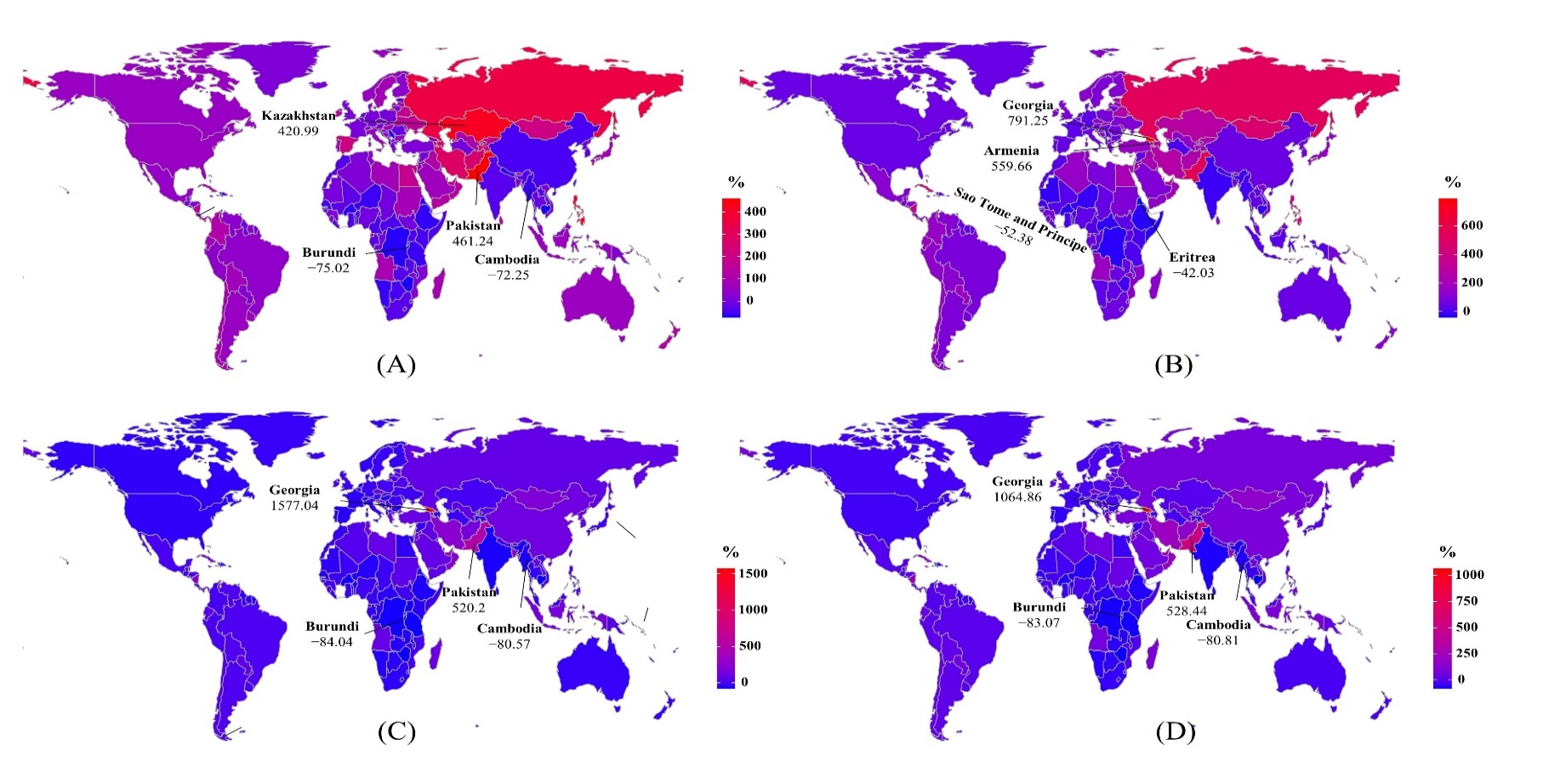
**

**Supplementary table 1**. Number of HIV/AIDS cases in 2004 and the percentage changes in the number of cases from 1990 to 2004 in different age groups globally.

| **Age Groups** | **Incidence** | | **Prevalence** | | **Death** | | **DALYs** | |
| --- | --- | --- | --- | --- | --- | --- | --- | --- |
|  | Number  ×10^3^(95% UI) | Change in number (%) | Number  ×10^3^(95% UI) | Change in number (%) | Number  ×10^3^(95% UI) | Change in number (%) | Number  ×10^3^(95% UI) | Change in number (%) |
| **﹤5** | 408.96(376.44-438.84) | 124.05 | 1023.09(924.97-1116.93) | 252.07 | 217.04(195.88-239.97) | 195.64 | 18986.68(17145.52-21024.02) | 195.36 |
| **5 to 9** | 0 | 0 | 704.49(609.36-797.35) | 1391.56 | 27.34(23.56-31.28) | 1097.48 | 2321.65(1998.4-2657.07) | 1107.24 |
| **10 to 14** | 0 | 0 | 378.51(319.6-436.84) | 15030 | 15.35(12.87-17.74) | 3480.81 | 1219.61(1026.97-1406.79) | 3587.85 |
| **15 to 19** | 296.26(269.99-333.26) | 22 | 1044.69(967.36-1135.67) | 97.25 | 26.29(13.93-43.74) | 230.2 | 2011.4(1134.27-3245.4) | 215.72 |
| **20 to 24** | 352.78(317.83-399.43) | 21.31 | 2854.88(2696.85-3043.77) | 164.83 | 79.54(47.41-127.84) | 259.16 | 5642.62(3515.67-8852.82) | 251.55 |
| **25 to 29** | 487.92(411.5-597.03) | 13.73 | 4422.68(4170.7-4682.4) | 179.5 | 194.98(115.06-287.77) | 375.87 | 12561.4(7723.61-18249.71) | 363.41 |
| **30 to 34** | 352.34(290.8-441.15) | 14.47 | 4889.64(4625.23-5165.62) | 238.08 | 269.34(178.31-368.26) | 467.14 | 15896.16(10948.19-21477.03) | 455.7 |
| **35 to 39** | 265.53(214.11-340.34) | 14.3 | 4380.59(4180.05-4590.1) | 298.43 | 288.06(185.96-395.03) | 567.44 | 15556.19(10578.27-20941.23) | 556.43 |
| **40 to 44** | 164.31(128.93-213.7) | 24.41 | 3350.77(3077.08-3678.86) | 397.38 | 254.83(187.1-304.75) | 691.95 | 12459.95(9612.66-14660.56) | 682.09 |
| **45 to 49** | 113.75(90.21-146.63) | 26.01 | 2155.51(1904.83-2429.09) | 422.66 | 195.47(148.64-247.57) | 719.13 | 8602.71(6751.53-10702.03) | 712.08 |
| **50 to 54** | 78.35(64.41-96.05) | 32.58 | 1245.63(1055.08-1462.24) | 370.77 | 122.96(110.16-133.97) | 628.54 | 4805.9(4440.79-5175.56) | 621.17 |
| **55 to 59** | 49.27(40.24-59.54) | 34.34 | 697.24(586.25-835.37) | 321.19 | 65.87(61.03-70.94) | 522.71 | 2267.4(2122.52-2419.22) | 516.37 |
| **60 to 64** | 31.56(26.17-38.18) | 36.39 | 416.4(351.28-499.65) | 301.61 | 38.93(36.13-42.14) | 481.99 | 1160.03(1081.03-1246.84) | 475.54 |
| **65 to 69** | 20.95(18.27-25.06) | 35.96 | 254.4(214.33-305.11) | 307 | 23.94(22.15-25.98) | 445.44 | 605.65(564.9-652.78) | 442.07 |
| **70 to 74** | 17.77(15.66-19.9) | 59.74 | 162.04(136.98-193.55) | 294.77 | 14.43(13.01-16.07) | 483.51 | 304.4(282.51-331.6) | 472.48 |
| **75 to 79** | 6.9(5.62-7.88) | 49.99 | 90.12(76.5-106.47) | 241.06 | 7.93(7.16-8.83) | 537.4 | 135.28(125.32-146.82) | 496.02 |
| **＞80** | 0 | 0 | 26.75(20.29-36.08) | 846.38 | 2.17(1.99-2.39) | 819.27 | 27.75(23.97-32.58) | 835.88 |

DALYs, disability-adjusted life years.

**Supplementary table 2**. Percentage changes in the absolute number and the EAPCs of HIV/AIDS prevalence from 1990 to 2019 globally, stratified by sex, SDI area, and geographic region.

| **Characteristics** | **2004** | | **1990-2004** | | **2019** | | **2005-2019** | |
| --- | --- | --- | --- | --- | --- | --- | --- | --- |
|  | Number  ×10^3^ (95% UI) | ASR/100,000  (95% UI) | Change in  number (%) | EAPC  (95%CI) | Number  ×10^3^ (95% UI) | ASR/100,000  (95% UI) | Change in  number (%) | EAPC  (95%CI) |
| **Overall** | 28097.43  (27040.94-29169.74) | 422.50  (406.10-438.89) | 258.90 | 7.47  (5.84-9.12) | 36848.15  (35149-38856.67) | 454.32  (433.76-478.59) | 30.31 | 0.71  (0.54-0.87) |
| **Sex** |  |  |  |  |  |  |  |  |
| Male | 12762.67  (11955.7-13615.11) | 383.86  (358.58-410.5) | 229.97 | 6.80  (5.38-8.25) | 16798.12  (15593.07-18379.22) | 412.33  (383-450.7) | 30.60 | 0.66  (0.52-0.81) |
| Female | 15334.76  (14742.23-15972.35) | 461.78  (444.11-481.06) | 287.14 | 8.06  (6.23-9.91) | 20050.03  (19266.71-20898.45) | 497.88  (478.37-518.72) | 30.07 | 0.76  (0.58-0.94) |
| **SDI** |  |  |  |  |  |  |  |  |
| Low | 9162.13  (8675.11-9722.74) | 1466.99  (1399.74-1546.6) | 122.82 | 2.60  (1.09-4.13) | 10216.98  (9686.28-10786.45) | 1154.52  (1098.27-1218.7) | 12.78 | −1.26  (−1.48-−1.05) |
| Low-middle | 7924.13  (7634.52-8223.74) | 579.27  (556.74-602.11) | 390.36 | 8.82  (6.22-11.48) | 8962.25  (8523.02-9497.67) | 517.17  (491.86-548.27) | 13.68 | −0.49  (−0.65-−0.32) |
| Middle | 8052.90  (7682.33-8433.7) | 365.63  (347.3-384.36) | 1399.42 | 18.36  (15.01-21.8) | 12501.85  (11868.37-13326.06) | 472.33  (447.99-503.74) | 50.47 | 1.83  (1.75-1.9) |
| High-middle | 1355.76  (1143.75-1638.93) | 92.68  (78.17-112.01) | 232.62 | 7.87  (6.75-9.00) | 2767.16  (2417.46-3248.22) | 165.78  (143.15-196.55) | 100.44 | 4.42  (3.78-5.06) |
| High | 1581.29  (892.44-2354.42) | 147.65  (82.64-218.67) | 37.60 | 0.85  (0.66-1.04) | 2373.03  (1348.02-3395.22) | 183.72  (105.1-265.36) | 46.14 | 1.46  (1.36-1.56) |
| **Regions** |  |  |  |  |  |  |  |  |
| East Asia | 342.36  (200.86-563.55) | 21.88  (12.79-36.01) | 402.91 | 9.89  (9.31-10.48) | 570.19  (294.16-1082.6) | 31.73  (16.54-59.78) | 47.80 | 1.50  (0.59-2.41) |
| South Asia | 2255.75  (2051.37-2487.11) | 160.03  (145.29-176.15) | 3618.35 | 25.27  (19.18-31.68) | 1937.50  (1691.09-2394.81) | 107.18  (93.46-131.09) | −12.04 | −2.48  (−3.08-−1.87) |
| Southeast Asia | 1167.52  (1026.52-1376.01) | 194.71  (170.45-229.73) | 839.56 | 10.84  (6.71-15.14) | 1715.25  (1476.88-2125.09) | 237.53  (205.49-293.09) | 43.62 | 1.25  (1.17-1.33) |
| Central Asia | 21.18  (14.44-29.8) | 26.70  (18.32-37.41) | 242.82 | 7.12  (6.40-7.85) | 63.24  (52.53-80.39) | 62.87  (52.31-78.72) | 182.11 | 5.96  (5.13-6.81) |
| High-income Asia Pacific | 38.05  (20.52-56.11) | 16.90  (9.21-25.05) | 412.13 | 10.96  (10.6-11.33) | 80.68  (47.95-115.35) | 30.87  (18.83-44.05) | 97.03 | 3.97  (3.72-4.21) |
| Oceania | 52.08  (3.7-137.15) | 618.21  (43.66-1650.82) | 10719.23 | 39.48  (34.22-44.95) | 97.26  (4.23-280.66) | 804.07  (35.13-2301.26) | 76.14 | 1.66  (1.61-1.72) |
| Australasia | 12.73  (8.69-16.69) | 45.75  (30.72-60.53) | 26.14 | −0.04  (−0.41-0.34) | 19.87  (13.2-27) | 55.71  (35.99-77.6) | 51.04 | 1.31  (1.21-1.40) |
| Eastern Europe | 305.86  (194.57-471.85) | 135.12  (85.64-209.68) | 298.05 | 12.05  (11.08-13.04) | 1436.47  (1175.12-1763.65) | 613.42  (493.02-765.06) | 343.93 | 11.81  (11-12.62) |
| Western Europe | 443.28  (323.3-571.23) | 93.54  (67.82-121.64) | 56.68 | 1.78  (1.54-2.02) | 677.42  (494.63-861.81) | 125.09  (88.95-161.16) | 48.39 | 1.97  (1.90-2.04) |
| Central Europe | 22.18  (16.92-28.73) | 17.56  (13.3-22.81) | 282.26 | 10.35  (9.94-10.77) | 39.96  (32.2-54.55) | 31.22  (24.77-42.95) | 68.44 | 3.57  (3.38-3.76) |
| High-income North America | 1252.62  (661.08-1922.2) | 332.29  (173.89-510.54) | 30.64 | 0.44  (0.26-0.62) | 1835.63  (982.21-2704.94) | 408.70  (219.92-605.67) | 43.23 | 1.40  (1.26-1.54) |
| Andean Latin America | 54.84  (39.85-73.93) | 111.97  (82.31-150.28) | 227.11 | 6.55  (6.08-7.03) | 139.11  (105.85-191.14) | 215.10  (166.36-294.83) | 140.39 | 4.76  (4.46-5.07) |
| Central Latin America | 222.61  (161.75-296.75) | 108.01  (79.85-142.12) | 270.66 | 5.79  (4.47-7.12) | 516.35  (420.65-629.39) | 198.67  (163.1-240.53) | 118.90 | 4.13  (4.10-4.15) |
| Caribbean | 269.70  (239.93-301.13) | 631.37  (562.04-707.13) | 136.93 | 3.39  (1.58-5.23) | 318.29  (275.17-367.45) | 647.06  (558.26-748.28) | 18.66 | 0.39  (0.31-0.47) |
| Tropical Latin America | 479.33  (393.50-542.96) | 244.74  (203.16-276) | 180.65 | 5.89  (5.60-6.19) | 907.19  (779.71-1064.49) | 362.77  (310.53-425.98) | 78.91 | 2.47  (2.38-2.55) |
| Southern Latin America | 119.39  (73.23-171.51) | 206.04  (126.07-296.13) | 177.83 | 5.84  (5.28-6.4) | 256.07  (150.56-382.25) | 357.22  (206.14-538.55) | 102.01 | 3.57  (3.45-3.70) |
| Eastern Sub- Saharan Africa | 8924.62  (8582.82-9343.45) | 4143.18  (3948.16-4350.36) | 128.07 | 2.63  (0.99-4.30) | 10452.88  (9962.64-10976.27) | 3422.91  (3268.72-3583.83) | 18.39 | −0.88  (−1.07-−0.69) |
| Southern Sub- Saharan Africa | 7255.72  (6921.97-7610.1) | 11334.01  (10803.04-11874.29) | 1261.10 | 17.52  (13.93-21.23) | 10292.82  (9799.86-10790.81) | 13291.18  (12661.43-13924.75) | 39.22 | 1.27  (1.17-1.38) |
| Western Sub- Saharan Africa | 3803.03  (3424.84-4227.81) | 1623.69  (1479.72-1777.09) | 333.33 | 7.19  (4.92-9.51) | 4192.28  (3945.74-4464.33) | 1248.18  (1166.93-1339.64) | 10.16 | −1.59  (−1.67-−1.51) |
| North Africa  and Middle East | 92.02  (50.58-172.08) | 20.74  (11.31-37.71) | 461.49 | 9.70  (8.54-10.88) | 203.88  (106.28-407.53) | 31.90  (16.81-62.46) | 110.35 | 2.88  (2.61-3.16) |
| Central Sub- Saharan Africa | 962.5  3(828.93-1102.92) | 1444.43  (1243.81-1655.92) | 99.46 | 1.33  (0.02-2.66) | 1095.82  (943.02-1261.69) | 1084.38  (933.09-1247.23) | 14.55 | −1.79  (−2.10-−1.47) |

EAPC: estimated annual percentage change; ASR, age-standardized rate; CI, confidence interval; UI: uncertainty interval; SDI: socio-demographic index.

**Supplementary table 3**. Percentage changes in the absolute number and the EAPCs of deaths caused by HIV/AIDS from 1990 to 2019 globally, stratified by sex, SDI area, and geographic region.

| Characteristics | **2004** | | **1990−2004** | | **2019** | | **2005−2019** | |
| --- | --- | --- | --- | --- | --- | --- | --- | --- |
|  | Number  ×10^3^ (95% UI) | ASR/100,000  (95% UI) | Change in  number (%) | EAPC  (95%CI) | Number  ×10^3^ (95% UI) | ASR/100,000  (95% UI) | Change in  number (%) | EAPC  (95%CI) |
| **Overall** | 1844.49  (1445.81-2283.2) | 28.21  (22.28-34.73) | 448.32 | 10.85  (8.9-12.84) | 863.84  (786.07-996.04) | 10.72  (9.7-12.39) | −52.89 | −6.73  (−6.98-−6.47) |
| **Sex** |  |  |  |  |  |  |  |  |
| Male | 828.73  (662.71-1018.78) | 25.24  (20.3-30.76) | 384.29 | 9.71  (7.92-11.53) | 435.39  (398.81-499.87) | 10.74  (9.81-12.4) | −47.16 | −5.78  (−6.07-−5.49) |
| Female | 1015.76  (785.87-1270.82) | 31.21  (24.32-38.78) | 514.63 | 11.89  (9.76-14.06) | 428.44  (382.91-501.68) | 10.72  (9.51-12.61) | −57.56 | −7.56  (−7.82-−7.3) |
| **SDI** |  |  |  |  |  |  |  |  |
| Low | 716.00  (553.83-889.63) | 119.88  (95.06-146.58) | 249.67 | 6.82  (4.85-8.83) | 267.80  (233.31-324.05) | 30.11  (27.06-35.09) | −61.5 | −9.10  (−9.26-−8.94) |
| Low-middle | 561.03  (435.17-695.11) | 42.77  (33.58-52.43) | 825.65 | 14.91  (11.76-18.16) | 255.79  (228.67-295.04) | 14.93  (13.52-17.01) | −53.83 | −7.28  (−7.74-−6.82) |
| Middle | 475.45  (370.09-600.98) | 22.41  (17.8-27.98) | 2667.49 | 24.82  (21.35-28.39) | 278.03  (252.27-325.77) | 10.53  (9.53-12.40) | −43.18 | −5.61  (−6.13-−5.1) |
| High-middle | 71.45  (62.72-81.23) | 4.90  (4.31-5.57) | 307.53 | 8.19  (6.22-10.19) | 50.80  (49.11-53.14) | 3.04  (2.93-3.20) | −32.09 | −3.57  (−3.83-−3.3) |
| High | 19.02  (18.84-19.39) | 1.75  (1.73-1.8) | −47.13 | −9.09  (−11.88-−6.2) | 10.63  (10.12-11.75) | 0.84  (0.80-0.96) | −42.15 | −4.99  (−5.86-−4.12) |
| **Regions** |  |  |  |  |  |  |  |  |
| East Asia | 13.84  (12.76-15.93) | 0.9  (0.83-1.04) | 405.74 | 9.19  (8-10.4) | 32.68  (26.11-39.65) | 1.74  (1.4-2.09) | 109.88 | 5.00  (3.76-6.26) |
| South Asia | 160.59  (124.06-201.88) | 11.97  (9.33-14.9) | 11123.82 | 40.82  (34.31-47.64) | 52.07  (42.18-82.54) | 2.89  (2.35-4.64) | −68.89 | −11.20  (−12.03-−10.36) |
| Southeast Asia | 70.2  (63.01-77.9) | 12.26  (11.2-13.5) | 3026.58 | 23.24  (17.09-29.72) | 42.95  (35.86-54.42) | 6.00  (4.96-7.70) | −37.58 | −3.83  (−4.44-−3.22) |
| Central Asia | 1.08  (1.05-1.11) | 1.4  (1.36-1.43) | 230.57 | 8.11  (5.72-10.56) | 1.28  (1.23-1.33) | 1.28  (1.23-1.33) | 7.88 | −1.12  (−1.63-−0.61) |
| High-income Asia Pacific | 0.31  (0.31-0.32) | 0.14  (0.14-0.14) | 141.89 | 5.74  (4.18-7.32) | 0.34  (0.33-0.35) | 0.13  (0.13-0.14) | 1.17 | −1.07  (−1.81-−0.33) |
| Oceania | 2.93  (1.04-6.37) | 36.11  (13.47-78.59) | 13765.02 | 44.76  (40.81-48.82) | 4.18  (1.35-11.31) | 35.61  (12.45-96.39) | 29.94 | −0.73  (−1-−0.45) |
| Australasia | 0.16  (0.16-0.16) | 0.58  (0.56-0.59) | −62.78 | −12.47  (−15.23-−9.62) | 0.08  (0.08-0.08) | 0.23  (0.23-0.24) | −39.45 | −5.65  (−6.9-−4.4) |
| Eastern Europe | 16.59  (16.42-16.77) | 7.03  (6.96-7.1) | 226.52 | 8.89  (7.62-10.17) | 25.99  (25.63-26.35) | 10.94  (10.80-11.09) | 25.99 | 1.91  (1.18-2.64) |
| Western Europe | 6.39  (6.32-6.46) | 1.32  (1.3-1.33) | −36.84 | −8.56  (−12.03-−4.97) | 3.22  (3.17-3.28) | 0.60  (0.59-0.61) | −46.87 | −5.26  (−6.03-−4.49) |
| Central Europe | 0.64  (0.62-0.68) | 0.52  (0.51-0.55) | 35.56 | 3.16  (−0.29-6.73) | 0.45  (0.42-0.50) | 0.35  (0.34-0.39) | −26.45 | −1.2  (−1.96-−0.44) |
| High-income North America | 14.87  (14.78-14.96) | 3.86  (3.83-3.88) | −47.98 | −9.22  (−11.97-−6.38) | 7.31  (7.23-7.39) | 1.64  (1.62-1.66) | −49.04 | −5.95  (−7.08-−4.8) |
| Andean Latin America | 3.62  (2.9-5.13) | 7.73  (6.22-11.11) | 369.15 | 9.12  (8.53-9.71) | 4.34  (2.82-8.27) | 6.77  (4.38-12.97) | 11.4 | −0.31  (−1.25-0.63) |
| Central Latin America | 12.03  (11.84-12.19) | 6.08  (5.99-6.16) | 212.59 | 5.02  (3.69-6.36) | 12.02  (11.46-12.52) | 4.66  (4.43-4.85) | −1.05 | −1.98  (−2.24-−1.71) |
| Caribbean | 21.04  (16.17-26.61) | 50.13  (38.81-62.89) | 314.04 | 7.84  (5.05-10.7) | 9.37  (7.89-11.45) | 19.03  (15.97-23.32) | −53 | −6.07  (−6.46-−5.68) |
| Tropical Latin America | 15.06  (14.91-15.22) | 7.87  (7.79-7.95) | 85.79 | 0.62  (−1.53-2.82) | 16.13  (15.78-16.5) | 6.47  (6.32-6.62) | 6.04 | −1.13  (−1.29-−0.97) |
| Southern Latin America | 2.34  (2.3-2.37) | 4.04  (3.98-4.09) | 228.71 | 7.17  (4.11-10.31) | 2.45  (2.41-2.49) | 3.35  (3.29-3.4) | 10.4 | −0.73  (−0.96-−0.51) |
| Eastern Sub- Saharan Africa | 687.9  (541.65-841.12) | 340  (273.69-407.79) | 266.49 | 7.3  (5.16-9.47) | 246.39  (217.71-293.24) | 80.12  (73.44-90.78) | −62.93 | −9.59  (−9.87-−9.31) |
| Southern Sub- Saharan Africa | 448.12  (333.19-577.88) | 750.56  (563.4-962.93) | 2559.41 | 24.33  (20.44-28.33) | 188.07  (164.26-227.05) | 247.28  (220.41-291.71) | −58.8 | −8.18  (−8.9-−7.45) |
| Western Sub- Saharan Africa | 282.73  (211.79-371.12) | 126.83  (97.43-163.3) | 663.55 | 12.64  (9.85-15.51) | 165.00  (138.98-198.99) | 50.66  (44.7-58.94) | −40.6 | −5.83  (−6.12-−5.53) |
| North Africa  and Middle East | 5.88  (3.4-11.31) | 1.38  (0.8-2.58) | 717.06 | 13.17  (11.68-14.68) | 9.43  (5.51-18.55) | 1.51  (0.88-3.04) | 50.84 | 0.49  (0.16-0.82) |
| Central Sub- Saharan Africa | 78.16  (59.72-98.02) | 124.32  (99.14-151.52) | 225.89 | 5.66  (3.62-7.73) | 40.09  (32.82-50.51) | 40.90  (34.54-49.44) | −47.66 | −7.21  (−7.61-−6.82) |

EAPC: estimated annual percentage change; ASR, age-standardized rate; CI, confidence interval; UI: uncertainty interval; SDI: socio−demographic index.

**Supplementary table 4**. Percentage changes in the number and the EAPCs of DALYs due to HIV/AIDS from 1990 to 2019 globally, stratified by sex, SDI areas, and geographic regions.

| **Characteristics** | **2004** | | **1990−2004** | | **2019** | | **2005−2019** | |
| --- | --- | --- | --- | --- | --- | --- | --- | --- |
|  | Number  ×10^3^ (95% UI) | ASR/100,000  (95% UI) | Change in  number (%) | EAPC  (95%CI) | Number  ×10^3^ (95% UI) | ASR/100,000  (95% UI) | Change in  number (%) | EAPC  (95%CI) |
| **Overall** | 104564.78  (83381.55-128579.68) | 1585.73  (1273.57-1939.58) | 399.93 | 10.4  (8.47-12.36) | 47632.18  (42630.99-55650.04) | 601.49  (536.16-703.92) | −54.03 | −6.75  (−6.95-−6.54) |
| **Sex** |  |  |  |  |  |  |  |  |
| Male | 46358.05  (37767.35-56492.53) | 1394.03  (1143.24-1691.28) | 348.38 | 9.4  (7.62-11.22) | 23345.78  (21108.9-27140.35) | 583.34  (525.67-679.55) | −49.2 | −5.92  (−6.15-−5.7) |
| Female | 58206.72  (45554.47-72576.39) | 1778.49  (1403.35-2202.89) | 450.32 | 11.27  (9.2-13.38) | 24286.40  (21357.67-28616.85) | 620.75  (543.97-733.98) | −57.89 | −7.44  (−7.65-−7.22) |
| **SDI** |  |  |  |  |  |  |  |  |
| Low | 41530  (32769.89-51434.58) | 6167.5  (4905.37-7575.67) | 222.51 | 6.27  (4.37-8.21) | 15551.58  (13229.66-18952.91) | 1578.46  (1387.12-1877.83) | −61.4 | −8.97  (−9.12-−8.82) |
| Low-middle | 31943.34  (25226.71-39462.47) | 2298.93  (1826.97-2823.81) | 698.98 | 13.99  (11-17.06) | 13950.23  (12220.88-16341.38) | 795.43  (703.3-921.08) | −55.7 | −7.36  (−7.75-−6.97) |
| Middle | 26152.95  (20821.43-32700.99) | 1216.11  (980.46-1502.98) | 2263.68 | 23.87  (20.49-27.35) | 14714.40  (13273.53-17332.75) | 568.70  (510.39-672.63) | −45.04 | −5.65  (−6.08-−5.21) |
| High-middle | 3877.89  (3448.77-4384.43) | 273.05  (243.65-308.55) | 288.01 | 8.14  (6.18-10.13) | 2752.78  (2618.34-2938.58) | 172.44  (163.6-184.18) | −31.99 | −3.5  (−3.74-−3.26) |
| High | 974.63  (906.9-1064.02) | 92.69  (86.07-101.24) | −49.11 | −9.09  (−11.76-−6.33) | 620.84  (521.8-747.41) | 51.78  (43.65-62.36) | −34.03 | −3.93  (−4.73-−3.13) |
| **Regions** |  |  |  |  |  |  |  |  |
| East Asia | 701.83  (641.67-815.99) | 46.24  (41.88-54.57) | 342.92 | 8.73  (7.68-9.8) | 1441.75  (1136.94-1811.66) | 82.37  (65.91-102.64) | 84.84 | 4.18  (3.22-5.15) |
| South Asia | 9038.19  (7110.32-11319.89) | 651.14  (515.39-809.42) | 7642.46 | 37.95  (32.07-44.1) | 2927.12  (2331.73-4676.77) | 160.06  (128.2-254.57) | −68.69 | −10.99  (−11.75-−10.22) |
| Southeast Asia | 3836.22  (3468.26-4257.77) | 647.96  (592.8-714.91) | 2315.67 | 21.31  (15.71-27.18) | 2392.33  (2057.9-2957.69) | 336.51  (288.05-419.04) | −36.43 | −3.62  (−4.13-−3.11) |
| Central Asia | 59.5  (57.87-61.54) | 76.04  (73.93-78.68) | 218.34 | 8.13  (5.83-10.49) | 71.15  (67.78-74.76) | 70.86  (67.48-74.47) | 9.78 | −1.03  (−1.48-−0.57) |
| High-income Asia Pacific | 16.21  (14.28-18.92) | 8.05  (7.19-9.23) | 140.75 | 5.84  (4.52-7.18) | 20.15  (15.98-25.93) | 8.82  (7.29-10.97) | 16.72 | 0.05  (−0.54-0.65) |
| Oceania | 166.75  (53.26-372.78) | 1890.9  (650.6-4158.86) | 10791.42 | 43.06  (39.14-47.09) | 221.64  (62.17-607.02) | 1775.74  (535.12-4894.8) | 21.72 | −1.08  (−1.41-−0.74) |
| Australasia | 7.98  (7.47-8.6) | 29.61  (27.78-31.88) | −63.87 | −12.30  (−14.98-−9.53) | 4.89  (4.16-5.91) | 14.80  (12.71-17.84) | −26.69 | −4.1  (−5.16-−3.03) |
| Eastern Europe | 886.07  (861.58-929.53) | 391.52  (381.33-410.14) | 221.93 | 9.00  (7.86-10.15) | 1453.87  (1399.24-1525.79) | 630.46  (606.93-661.93) | 30.84 | 1.99  (1.33-2.65) |
| Western Europe | 333.19  (315.5-358.09) | 71.51  (67.82-76.69) | −39.31 | −8.55  (−11.88-−5.08) | 185.94  (163.52-215.29) | 36.76  (32.62-42.35) | −41.13 | −4.38  (−5.18-−3.57) |
| Central Europe | 35.8  (34.43-37.7) | 31.28  (30.09-32.83) | 26.45 | 2.98  (−0.99-7.12) | 24.40  (22.48-28.33) | 21.11  (19.48-23.99) | −26.53 | −1.16  (−1.87-−0.44) |
| High-income North America | 758.64  (703.51-833.38) | 201.91  (187.25-221.44) | −50.49 | −9.28  (−11.92-−6.56) | 433.18  (362.15-530.23) | 102.00  (85.78-123.58) | −40.7 | −4.7  (−5.73-−3.65) |
| Andean Latin America | 210.6  (171.4-290.4) | 429.64  (350.42-599.9) | 345.9 | 9.28  (8.74-9.82) | 259.66  (163.34-492.51) | 400.96  (251.36-762.66) | 14.66 | 0.06  (−0.75-0.87) |
| Central Latin America | 652.56  (639.35-673.37) | 316.51  (309.99-326.64) | 201.86 | 5.13  (3.76-6.51) | 632.11  (605.35-661.07) | 244.00  (233.5-255.72) | −3.87 | −1.9  (−2.15-−1.66) |
| Caribbean | 1148.2  (890.25-1452.59) | 2711.24  (2114.25-3421.15) | 275.4 | 7.37  (4.7-10.1) | 484.66  (403.46-602.61) | 1002.14  (828.7-1249.77) | −55.41 | −6.34  (−6.77-−5.9) |
| Tropical Latin America | 828.25  (807.92-851.56) | 425.56  (414.98-437.56) | 70.18 | 0.26  (−1.85-2.41) | 840.17  (802.38-885.86) | 342.53  (326.79-361.83) | 1.27 | −1.31  (−1.47-−1.16) |
| Southern Latin America | 128.1  (121.91-137.01) | 221.86  (211.2-237.3) | 221.76 | 7.1  (3.96-10.34) | 138.08  (123.61-162.5) | 192.41  (171.92-226.98) | 13.33 | −0.44  (−0.63-−0.25) |
| Eastern Sub- Saharan Africa | 40153.25  (32393.45-49085.27) | 17265.5  (14013.41-20878.49) | 235.56 | 6.68  (4.61-8.78) | 14398.36  (12394.14-17302.74) | 4141.16  (3732.24-4808.22) | −62.85 | −9.45  (−9.69-−9.2) |
| Southern Sub- Saharan Africa | 24874.34  (18952.56-31806.64) | 39114.99  (29955.71-49535.78) | 2072.38 | 22.79  (19.1-26.59) | 10110.57  (8689.81-12327.66) | 12776.70  (11185.64-15298.06) | −59.88 | −8.2  (−8.83-−7.56) |
| Western Sub- Saharan Africa | 15963.28  (12048.19-20896.17) | 6388.74  (4905.13-8291.41) | 590.77 | 11.9  (9.19-14.68) | 8861.10  (7347.94-10890.25) | 2460.72  (2128.06-2914.02) | −43.55 | −6.13  (−6.34-−5.91) |
| North Africa  and Middle East | 343.05  (195.91-681.36) | 74.84  (43.09-147.63) | 642.58 | 12.86  (11.43-14.29) | 509.13  (292.58-1024.99) | 79.29  (45.26-160.62) | 39.95 | 0.24  (−0.12-0.61) |
| Central Sub- Saharan Africa | 4422.76  (3364.24-5583.93) | 6230.13  (4929.97-7704.55) | 198 | 5.13  (3.18-7.11) | 2221.93  (1777.11-2839.56) | 2050.21  (1695.81-2546.25) | −48.76 | −7.23  (−7.66-−6.79) |

EAPC: estimated annual percentage change; ASR, age-standardized rate; CI, confidence interval; UI: uncertainty interval; SDI: socio-demographic index; DALYs: disability-adjusted life years.

**Supplementary table 5**. Number of HIV/AIDS cases in 2019, and the percentage changes in the number of cases from 2005 to 2019 in different age groups globally.

| **Age Groups** | **Incidence** | | **Prevalence** | | **Death** | | **DALYs** | |
| --- | --- | --- | --- | --- | --- | --- | --- | --- |
|  | Number  ×10^3^(95% UI) | Change in number (%) | Number  ×10^3^(95% UI) | Change in number (%) | Number  ×10^3^(95% UI) | Change in number (%) | Number  ×10^3^(95% UI) | Change in number (%) |
| **﹤5** | 126.95(109.01-147.27) | −67.8 | 442.68(397.28-488.72) | −55.79 | 48.93(38.63-61.26) | −76.53 | 4304.2(3409.64-5375.1) | −76.4 |
| **5 to 9** | 0.31(0.3-0.32) | 0 | 581.32(530.85-628.91) | −19.98 | 12.38(10.05-15.02) | −55.22 | 1081.97(889.33-1299.95) | −53.95 |
| **10 to 14** | 0.18(0.17-0.19) | 0 | 670.2(621-716.77) | 57.05 | 15.11(12.85-17.64) | −9.96 | 1235.12(1060.49-1435.76) | −7.48 |
| **15 to 19** | 183.99(161.22-214.8) | −35.17 | 1048.57(976.21-1127.88) | 2.86 | 27.66(17.3-40.64) | −1.37 | 2098.35(1347.42-3021.43) | −1.7 |
| **20 to 24** | 238.45(210.94-271.29) | −30.54 | 2075.39(1917.06-2254.63) | −24.65 | 34.87(24.87-49.12) | −54.38 | 2558.75(1895.62-3500.75) | −52.84 |
| **25 to 29** | 416.63(363.17-480.19) | −12.96 | 3742.74(3476.67-4162.48) | −13.92 | 64.18(40.58-94.37) | −66.1 | 4370.65(2975.26-6225.37) | −64.19 |
| **30 to 34** | 351.85(306.67-401.86) | 2.5 | 5259.88(4911.78-5718.46) | 7.91 | 102.75(76.95-137.81) | −61.3 | 6396.59(4970.89-8383.98) | −59.21 |
| **35 to 39** | 254.35(199.4-312.29) | −1.1 | 5734.74(5465.31-6086.99) | 29.99 | 125.61(95.19-165.13) | −56.3 | 7132.23(5610.98-9124.27) | −54.08 |
| **40 to 44** | 138.35(109.41-172.6) | −13.13 | 5088.83(4800.07-5438.94) | 48.52 | 123.86(109.68-140.93) | −51.68 | 6348.47(5700.95-7091.15) | −49.39 |
| **45 to 49** | 88.71(68.93-114.37) | −19.74 | 4084.84(3840.26-4335.53) | 82.25 | 109.14(92.64-127.11) | −45.1 | 5014.35(4280.93-5823.12) | −42.73 |
| **50 to 54** | 60.32(46.53-76.17) | −22.27 | 2975.86(2767.93-3194.63) | 125.08 | 81.01(72.22-94) | −35.51 | 3320.86(2951.97-3874.28) | −32.44 |
| **55 to 59** | 51.87(36.05-69.63) | 3.72 | 2120.67(1923.36-2326.59) | 188.91 | 50.79(45.21-59.55) | −23.59 | 1863.16(1649.35-2188.42) | −18.67 |
| **60 to 64** | 38.02(27.4-49.43) | 19.83 | 1389.25(1230.52-1566.73) | 220.11 | 31.17(27.57-36.59) | −19.5 | 1005.5(884.41-1184.43) | −12.99 |
| **65 to 69** | 24.3(18.94-30.72) | 17.61 | 851.35(738.78-969.81) | 220.91 | 19.44(17.29-22.51) | −18.22 | 538.52(472.79-632.28) | −10.63 |
| **70 to 74** | 11.14(9.15-13.63) | −37.77 | 454.48(392.48-517.31) | 169.94 | 9.85(8.65-11.52) | −30.86 | 233.49(201.67-276.9) | −22.5 |
| **75 to 79** | 3.86(3.07-4.86) | −49.05 | 223.22(194.61-254.41) | 134.87 | 4.91(4.30-5.79) | −37.53 | 96.27(82.62-115.48) | −28.48 |
| **＞80** | 0 | 0 | 104.14(88.04-123.53) | 263.55 | 2.20(1.81-2.66) | 0.01 | 33.71(26.73-42.67) | 19.37 |

DALYs, disability-adjusted life years.

**Supplementary table 6**. Age-standardized rate of HIV/AIDS incidence at the national level and for both sexes in 2019, and the percentage changes in the incidence and the EAPCs from 1990 to 2019.

|  | **1990-2004** | | | **2005−2019** | | |
| --- | --- | --- | --- | --- | --- | --- |
| **Characteristics** | ASR/100,000  (95% UI) | Change in  number (%) | EAPC  (95%CI) | ASR/100,000  (95% UI) | Change in  number (%) | EAPC  (95%CI) |
| Afghanistan | 1.12(0.09-4.5) | 165.98 | 1.96(1.36-2.56) | 2.35(0.11-9.86) | 247.75 | 6.94(5.59-8.31) |
| Albania | 0.11(0.1-0.13) | 4.19 | 0.03(−0.89-0.95) | 0.1(0.08-0.15) | −9.65 | 0.82(−0.23-1.87) |
| Algeria | 1.77(0.25-8.16) | 373.73 | 9.52(9.11-9.93) | 1.61(0.01-7.69) | 9.01 | −1.54(−2.82-−0.23) |
| American Samoa | 2.14(0.81-4.46) | 93.2 | 4.29(2.76-5.85) | 2.41(0.97-5.09) | 1.48 | 0.44(−0.16-1.03) |
| Andorra | 4.49(0.26-22) | −25.05 | −2.58(−4.73-−0.39) | 9.65(0.35-52.39) | 15.5 | 4.71(2.8-6.67) |
| Angola | 137.05(92.05-199.32) | 675.44 | 10.42(7.72-13.19) | 175.51(89.09-302.97) | 94.66 | 1.84(1.68-2) |
| Antigua and Barbuda | 12.24(8.1-15.22) | −34.17 | −4.98(−6.04-−3.91) | 28.96(19.9-36.23) | 175.69 | 7.18(5.63-8.76) |
| Argentina | 18.69(11.15-28.82) | 38.73 | 1.56(1.11-2.01) | 25.78(12.85-43.9) | 58.74 | 1.83(1.43-2.23) |
| Armenia | 1.08(0.45-1.9) | 1586.64 | 28.77(24.58-33.09) | 3.79(3.02-5.01) | 235.99 | 9.99(6.57-13.52) |
| Australia | 4.64(2.94-6.72) | −18.61 | −1.65(−2.96-−0.33) | 6.1(3.52-9.18) | 62.15 | 2.04(1.57-2.5) |
| Austria | 7.94(4.84-12.03) | 6.37 | 1.57(−1.34-4.57) | 7.78(4.37-11.58) | 13.01 | 0.63(−0.31-1.57) |
| Azerbaijan | 0.9(0.62-1.34) | 43.45 | 2.52(1.17-3.89) | 2.48(1.69-3.96) | 240.8 | 8.05(6.09-10.05) |
| Bahamas | 64.28(57.04-74.19) | −27.55 | −5.91(−7.99-−3.78) | 66.55(57.2-82.13) | 20.96 | 1.01(0.43-1.59) |
| Bahrain | 1.05(0.88-1.22) | −23.08 | −5.75(−6.21-−5.28) | 0.88(0.74-1.12) | 59.98 | −0.45(−0.84-−0.06) |
| Bangladesh | 0.39(0.02-1.9) | 652.42 | 23.57(19.35–27.94) | 0.39(0.01-2.14) | 10.78 | −1.68(−2.49-−0.87) |
| Barbados | 29.89(22.95-41.06) | −2.25 | −1.32(−1.93-−0.71) | 26.42(21.05-33.93) | 3.98 | 0.98(0.23-1.74) |
| Belarus | 7.32(5.7-9.39) | 176.23 | 11.44(9.16-13.78) | 19.65(14.49-26.92) | 178.2 | 9.89(8.43-11.37) |
| Belgium | 9.89(5.41-15.73) | 37.33 | 0.23(−0.94-1.42) | 8.1(3.89-12.46) | −21.89 | −2.09(−2.87-−1.3) |
| Belize | 49.28(42.93-59.73) | 143.12 | 4.97(3.27-6.69) | 47.44(41.36-58.17) | 83.57 | 3.07(1.98-4.16) |
| Benin | 84.36(63.72-111.32) | 219.07 | −1.4(−5.97-3.38) | 28.25(14.11-50.55) | −47.89 | −6.98(−7.35-−6.6) |
| Bermuda | 12.73(9.54-14.88) | −67.4 | −7.06(−7.28-−6.85) | 25.28(18.87-30.61) | 94.09 | 5.31(4.21-6.41) |
| Bhutan | 6.5(0.35-28.64) | 76.51 | 3.47(2.59-4.36) | 8.94(0.31-47.54) | 58.66 | 1.93(1.53-2.32) |
| Bolivia  (Plurinational State of) | 8.78(1.33-33.81) | 6 | −3.64(−4.27-−3.01) | 12.73(1.32-57.55) | 75.74 | 1.23(−0.05-2.54) |
| Bosnia and Herzegovina | 0.14(0.09-0.17) | 1.06 | −0.49(−2.31-1.36) | 0.23(0.17-0.31) | 59.84 | 4.56(2.64-6.52) |
| Botswana | 1087.08(899.44-1363.87) | 10.73 | −4.62(−6.76-−2.43) | 357.59(169.42-654.29) | −49.64 | −6.91(−7.01-−6.8) |
| Brazil | 23.35(18.53-28.15) | 68.09 | 4.13(2.49-5.8) | 27.6(21.7-33.74) | 39.89 | 1.66(1.11-2.21) |
| Brunei Darussalam | 5.19(3.32-7.61) | 309.64 | 8.67(6.36-11.03) | 8.15(4.68-12.91) | 108.04 | 4.27(3.72-4.83) |
| Bulgaria | 1.51(1.37-1.69) | 16.12 | 1.99(1.55-2.44) | 2.66(2.02-3.4) | 60.76 | 5.89(4.61-7.19) |
| Burkina Faso | 65.18(48.25-88.83) | −71.05 | −11.2(−11.8-−10.59) | 13.16(7.46-22.84) | −63.37 | −10.16(−10.28-−10.04) |
| Burundi | 121.67(91.21-162.57) | −78.63 | −13.92(−14.61-−13.21) | 15.56(8.86-26.36) | −75.02 | −12.93(−13.23-−12.63) |
| Cabo Verde | 54.35(27.41-93.14) | −5.64 | −4.35(−5.07-−3.61) | 21.07(5.83-63.31) | −45.01 | −6(−6.25-−5.76) |
| Cambodia | 26.85(9.83-48.92) | 249.79 | −4.63(−14.65-6.56) | 5.12(0.93-12.18) | −72.25 | −12.6(−13.89-−11.28) |
| Cameroon | 345.53(259.43-453.72) | 258.26 | 2.85(−0.71-6.53) | 112.57(70.27-163.88) | −45.29 | −7.31(−7.67-−6.96) |
| Canada | 7.11(4.7-9.86) | −23.28 | −0.93(−1.89-0.04) | 9.81(4.78-15.38) | 58.85 | 4.21(3.12-5.31) |
| Central African Republic | 356.27(263.02-469.73) | −62.25 | −9.16(−10.87-−7.42) | 147.94(61.8-303.5) | −47.13 | −5.8(−6.06-−5.53) |
| Chad | 129.01(89.63-185.99) | 53.71 | −1.83(−3.18-−0.46) | 70.82(38.04-123.53) | −12.28 | −3.72(−3.94-−3.49) |
| Chile | 11.82(7.43-17.68) | 134.87 | 5.26(4.88-5.63) | 21.31(11.79-34.29) | 96.09 | 3.79(3.52-4.07) |
| China | 4.32(2.77-6.29) | 551.36 | 12.98(11.81-14.17) | 2.21(1.16-3.66) | −50.06 | −5.57(−6.62-−4.51) |
| Colombia | 9.41(7.72-10.8) | 98.11 | 0.77(−2.03-3.65) | 17.45(13.63-22.73) | 113.57 | 4.84(3.89-5.79) |
| Comoros | 0.79(0.03-4.32) | 1174.35 | 20.6(17.85-23.42) | 1.81(0.05-9.48) | 221.71 | 10.79(6.19-15.59) |
| Congo | 191.58(141.79-258.31) | −47.17 | −7.76(−8.75-−6.75) | 127.35(47.66-286.02) | −6.35 | −2.57(−2.65-−2.49) |
| Cook Islands | 6.55(0.51-30.64) | 402.11 | 12.77(12.15-13.39) | 26.81(1.12-109.03) | 235.17 | 9.58(9.35-9.8) |
| Costa Rica | 7.49(6.42-9.21) | 64.05 | 1.49(0.96-2.02) | 8.1(6.47-10.41) | 23.18 | 0.15(−0.35-0.64) |
| Croatia | 0.82(0.64-1.02) | 4.19 | −0.82(−2.18-0.55) | 0.92(0.57-1.28) | −7.74 | −1.43(−2.57-−0.28) |
| Cuba | 6.5(4.1-9.51) | 308.66 | 11.01(8.67-13.4) | 16.36(9.22-25.95) | 116.88 | 5.78(3.99-7.6) |
| Cyprus | 0.62(0.48-0.83) | 70.7 | 1.68(0.6-2.77) | 0.91(0.58-1.45) | 72.46 | 3.48(2.47-4.5) |
| Czechia | 0.66(0.48-0.87) | 290.59 | 9.32(7.68-10.98) | 0.85(0.5-1.32) | 8.71 | 1.29(0.43-2.17) |
| Cote d'Ivoire | 257.13(193.93-349.03) | −48.57 | −10.38(−11.41-−9.33) | 58(35.3-90.7) | −68.84 | −9.42(−9.9-−8.95) |
| Democratic People's Republic of Korea | 4.45(0.1-27.04) | 662.49 | 11.89(9.56-14.27) | 3.55(0.03-22.61) | −13.05 | −1.36(−2.05-−0.67) |
| Democratic Republic of  the Congo | 84.13(58.6-119.29) | −43.59 | −7.59(−7.98-−7.2) | 16.46(8.51-27.98) | −67.57 | −10.33(−10.91-−9.75) |
| Denmark | 3.77(2.35-5.46) | −32.9 | −3.34(−3.96-−2.71) | 4.21(2.33-6.42) | 11.27 | 0.28(−0.54-1.11) |
| Djibouti | 166.3(100.85-253.23) | 2379.98 | 14.78(4.69-25.85) | 69.86(26.04-159.24) | −30.75 | −5.76(−5.97-−5.54) |
| Dominica | 15.17(11.19-17.88) | −35.39 | −2.93(−3.23-−2.63) | 28.18(21.34-34.03) | 91.52 | 4.78(3.49-6.08) |
| Dominican Republic | 53.18(40.67-70.24) | −44.37 | −6.88(−7.88-−5.86) | 25.08(9-55.51) | −39.41 | −4.77(−5.08-−4.46) |
| Ecuador | 13.97(11.75-16.7) | 488.83 | 11.03(9.91-12.17) | 19.12(15.32-24.31) | 71.46 | 1.99(1.7-2.27) |
| Egypt | 0.22(0.19-0.25) | 18.31 | −1.85(−2.66-−1.04) | 0.53(0.37-0.7) | 178.35 | 6.62(6.14-7.1) |
| El Salvador | 32.32(22.3-39.88) | 233.7 | 7.74(5.93-9.58) | 17.17(11.21-23.17) | −39.13 | −6.12(−7.27-−4.95) |
| Equatorial Guinea | 467.71(338.63-624.81) | 1013.28 | 12.6(9.86-15.4) | 625.16(236.28-1369.94) | 144.05 | 2.09(1.82-2.37) |
| Eritrea | 68.8(43.68-101.63) | −44 | −9.97(−11.47-−8.44) | 16.46(7.47-32.09) | −64.07 | −9.14(−9.23-−9.04) |
| Estonia | 15.55(13.81-17.79) | 7044.23 | 42.8(39.6-46.08) | 15.19(11.93-18.99) | −18.17 | −0.56(−1.07-−0.04) |
| Eswatini | 1963.5(1612.3-2458.48) | 1759 | 14.22(6.58-22.39) | 574.03(339.03-924.21) | −63.96 | −8.5(−9.07-−7.93) |
| Ethiopia | 112.16(87.89-148.96) | −51.53 | −10.77(−12.71-−8.79) | 26.38(19.66-34.89) | −61.59 | −9.26(−9.37-−9.14) |
| Fiji | 3.01(2.33-3.77) | 67.33 | 3.26(2.38-4.14) | 4.69(3.51-6.08) | 56.15 | 3.62(3.19-4.07) |
| Finland | 0.84(0.57-1.21) | 22.42 | 1.49(1.08-1.91) | 1.16(0.62-1.82) | 34.34 | 1.4(0.84-1.96) |
| France | 4.19(2.83-5.86) | −29.49 | −0.64(−2.3-1.04) | 4.25(2.43-6.58) | 3.6 | 0.79(0.39-1.19) |
| Gabon | 286.9(209.77-390.23) | 116.49 | 1.33(−0.97-3.68) | 140.6(55.03-292.05) | −31.04 | −4.73(−5.18-−4.28) |
| Gambia | 168.19(101.31-249.99) | 574.84 | 9.42(5.63-13.35) | 111.2(53.15-196.8) | −1.61 | −2.67(−2.74-−2.59) |
| Georgia | 2.89(1.42-5.13) | 728.86 | 25.48(19.97-31.23) | 6.94(4.42-12.07) | 61.91 | 2.38(−1.71-6.63) |
| Germany | 2.32(1.65-3.24) | −36.67 | −4.14(−5.37-−2.9) | 3.02(1.85-4.59) | 4.65 | 0.38(−0.09-0.85) |
| Ghana | 151.97(115.48-199.31) | 48.73 | −1.5(−3.25-0.28) | 56.46(35.34-86.35) | −42.67 | −6.61(−6.74-−6.48) |
| Greece | 1.16(0.88-1.62) | 44.62 | 2.1(1.27-2.93) | 1.84(1.3-2.85) | 6.28 | −0.1(−2.17-2.01) |
| Greenland | 12.37(7.78-18.26) | −22.48 | −0.45(−1.78-0.9) | 21.29(9.72-36.07) | 70.61 | 4.07(3.07-5.08) |
| Grenada | 12.7(8.79-15.34) | 1.34 | −2.81(−3-−2.63) | 18.31(12.65-23.06) | 45.25 | 3.49(1.68-5.34) |
| Guam | 5.82(2.51-11.41) | 18.34 | 1.6(−0.4-3.64) | 7.29(3.18-15.18) | 19.54 | 1.41(1.07-1.76) |
| Guatemala | 17.58(12.45-25.81) | 263.91 | 7.79(5.98-9.63) | 9.97(4.93-20.39) | 12.3 | −1.76(−3.77-0.29) |
| Guinea | 132.11(96.15-175.56) | 293.51 | 6.55(4.21-8.94) | 96.08(46.82-174.68) | 3.65 | −2.07(−2.28-−1.87) |
| Guinea-Bissau | 213.63(123.32-332.44) | 263.47 | 5.25(1.86-8.75) | 86.13(28.84-170.53) | −41.41 | −6.2(−6.68-−5.72) |
| Guyana | 94.8(68.89-119.69) | 96.34 | 4.78(3.04-6.56) | 76.66(52.17-97.91) | −14.03 | −2.86(−3.46-−2.26) |
| Haiti | 174.11(131.87-230.78) | −39.47 | −7(−7.45-−6.55) | 65.07(30.71-115.49) | −46.04 | −6.68(−7.07-−6.29) |
| Honduras | 7.88(5.18-10.62) | 66.51 | −0.5(−1.65-0.67) | 5.47(3.65-7.76) | 13.2 | −3.26(−4.14-−2.38) |
| Hungary | 0.86(0.68-1) | −8.64 | −1.28(−1.89-−0.67) | 1.52(1-1.97) | 84.26 | 5.42(4.29-6.57) |
| Iceland | 1.9(1.42-2.7) | −32.99 | −4.14(−5.5-−2.76) | 2.6(1.66-4.09) | −1.9 | 1.13(−1.78-4.12) |
| India | 11.34(6.71-17.83) | 384.53 | 6.77(−0.68-14.77) | 5.01(3.05-7.88) | −37.88 | −3.62(−4.25-−2.98) |
| Indonesia | 3.3(2.59-4.37) | 200.19 | 8.87(4.58-13.34) | 5.31(4.35-6.96) | 63.23 | 3.34(2.4-4.28) |
| Iran  (Islamic Republic of) | 1.34(1.03-1.84) | 665.71 | 15.02(12.76-17.33) | 3.91(2.72-5.44) | 278.57 | 9.73(8.7-10.78) |
| Iraq | 0.38(0.2-1.02) | 433.7 | 9.67(8.35-11) | 0.57(0.21-1.39) | 115.64 | 3.18(2.83-3.53) |
| Ireland | 1.19(0.68-2) | −16.01 | −4.22(−5.01-−3.42) | 3.01(1.41-4.67) | 142.09 | 7.71(7.07-8.35) |
| Israel | 7.22(4.19-11.06) | 127.97 | 3.62(2.25-5.01) | 7.25(3.89-10.17) | 17.48 | −0.86(−1.21-−0.52) |
| Italy | 4.13(2.74-5.94) | −60.5 | −4.61(−8.57-−0.48) | 6.76(4.26-10.42) | 57.42 | 3.62(3.44-3.8) |
| Jamaica | 28.99(22.7-33.22) | −6.69 | −2.55(−3.12-−1.99) | 34.98(27.31-45.86) | 31.38 | 0.63(−0.03-1.29) |
| Japan | 0.96(0.53-1.42) | 127.44 | 5.01(4.15-5.87) | 1.74(0.89-2.58) | 82.06 | 4.52(3.16-5.9) |
| Jordan | 0.51(0.37-0.63) | 260.18 | 7.97(7.09-8.85) | 0.46(0.31-0.61) | 78.2 | −1.68(−2.81-−0.54) |
| Kazakhstan | 2.7(2.37-3.2) | −1.55 | −0.01(−0.67-0.65) | 11.59(7.73-15.52) | 420.99 | 13(11.52-14.5) |
| Kenya | 425.16(364.82-501.47) | −42.68 | −9.24(−10.03-−8.44) | 139.55(104.77-181.92) | −49.89 | −7.29(−7.51-−7.07) |
| Kiribati | 2.45(1.95-2.92) | 5.89 | 0.36(−1.23-1.97) | 3.18(2.09-5.17) | 60.16 | 1.81(1.15-2.47) |
| Kuwait | 0.37(0.25-0.51) | 1.18 | 0.24(−0.86-1.35) | 0.28(0.23-0.35) | 86.27 | 0.56(−0.06-1.2) |
| Kyrgyzstan | 5.55(3.89-8.02) | 312.25 | 8.07(7.57-8.58) | 14.14(9.31-20.69) | 182.53 | 4.74(3.27-6.24) |
| Lao People's Democratic Republic | 16.84(0.38-98.89) | 11342.3 | 36.94(32.01-42.04) | 8.03(0.13-46.01) | −36.23 | −6.52(−7.55-−5.49) |
| Latvia | 9.2(7.52-11.1) | 30.28 | 1.97(−1.23-5.28) | 22.93(19.02-28.05) | 89.39 | 6.65(5.65-7.66) |
| Lebanon | 2.06(0.14-8.76) | −13.22 | −3.08(−3.59-−2.58) | 2.38(0.1-9.63) | 52.78 | 2.35(1.34-3.36) |
| Lesotho | 1665.27(1402.53-2002.31) | 275.03 | 6.02(2.06-10.14) | 929.95(625.55-1296.7) | −32.71 | −3.73(−3.95-−3.51) |
| Liberia | 124.88(89.07-171.35) | 169.42 | 1.44(−1.42-4.38) | 54.02(26.17-103.26) | −31.73 | −5.19(−5.4-−4.98) |
| Libya | 1.6(0.1-7.89) | 285.07 | 8.49(7.08-9.93) | 2.4(0.09-11.03) | 81.73 | 3.88(3.34-4.43) |
| Lithuania | 4.68(3.77-5.6) | 142.38 | 7.53(7.23-7.84) | 6.12(2.17-8.92) | 3.09 | 1.12(0.62-1.62) |
| Luxembourg | 1.97(1.16-3.37) | −67.85 | −9.15(−10.26-−8.03) | 3.9(1.98-6.08) | 191.06 | 6.12(5.58-6.66) |
| Madagascar | 18.03(9.05-30.31) | 5480.3 | 27.16(19.17-35.69) | 22.87(9.17-44.14) | 90.54 | 2.13(1.99-2.27) |
| Malawi | 721.1(580.82-912.66) | −23.87 | −5.26(−6.14-−4.38) | 197.7(123.93-292.87) | −59.66 | −8.51(−9-−8.01) |
| Malaysia | 14.06(10.82-16.87) | 55.87 | −1.59(−3.99-0.88) | 14.79(8.99-18.57) | 38.73 | 0.76(−0.35-1.89) |
| Maldives | 0.4(0.33-0.46) | 265.23 | 7.25(6.34-8.16) | 0.27(0.21-0.37) | 21.06 | −1.57(−2.48-−0.64) |
| Mali | 90.81(64.16-124.44) | 79.88 | −0.47(−2.92-2.03) | 36.88(17.23-66.54) | −31.17 | −5.63(−5.91-−5.35) |
| Malta | 3.26(2.11-4.5) | 105.8 | 3.99(3.18-4.81) | 6.27(3.53-9.39) | 85.68 | 3.9(3.53-4.26) |
| Marshall Islands | 6.6(0.09-39.67) | 56.02 | 0.03(−1.67-1.76) | 16.96(0.22-116.36) | 154.66 | 6.61(6.06-7.15) |
| Mauritania | 2.85(0.1-16.37) | −29.41 | −3.26(−6.32-−0.1) | 1.91(0.06-10.26) | 0.14 | −3.4(−3.92-−2.88) |
| Mauritius | 10.07(7.62-13.82) | 2564.33 | 30.36(26.77-34.05) | 14.88(11.92-19.28) | 36.88 | 1.62(−1.93-5.29) |
| Mexico | 9.76(9.16-10.49) | −36.63 | −4.19(−7.38-−0.9) | 12.86(10.23-16.2) | 51.13 | 2.07(1.93-2.22) |
| Micronesia  (Federated States of) | 55.23(0.47-396.85) | 859.41 | 12.83(6.25-19.81) | 94.67(1.34-633.99) | 82.03 | 4.8(0.95-8.79) |
| Monaco | 3.38(0.67-10.73) | 2.1 | 1.68(−1.43-4.89) | 4.49(0.78-16.98) | 28.97 | 2.17(1.8-2.54) |
| Mongolia | 0.45(0.16-0.9) | 4978.92 | 14.69(3.32-27.3) | 1.15(0.33-2.36) | 204.6 | 5.75(2.23-9.39) |
| Montenegro | 0.89(0.67-1.07) | 97.16 | 3.73(2.18-5.31) | 0.9(0.68-1.19) | −4.54 | 0.9(0.26-1.55) |
| Morocco | 4.89(0.32-21.06) | 288.87 | 8.44(7.28-9.61) | 2.58(0.09-11.72) | −39.09 | −4.43(−5.34-−3.51) |
| Mozambique | 829.49(682.73-1026.95) | 543.66 | 9.07(6.25-11.98) | 693.21(381.23-1189.47) | 15.85 | −0.9(−1.6-−0.2) |
| Myanmar | 47.15(35.78-63.07) | 259.88 | 6.41(3.5-9.4) | 15.74(11.66-21.37) | −58.28 | −6.98(−7.24-−6.72) |
| Namibia | 911.86(778.85-1109.03) | 313.98 | 4.97(0.47-9.68) | 262.11(180.11-361.08) | −60.29 | −7.78(−8.15-−7.41) |
| Nauru | 6.62(0.51-30.95) | 436.85 | 12.78(11.99-13.58) | 26.63(1.11-111.39) | 252.6 | 9.3(9.08-9.51) |
| Nepal | 20.82(1.15-96.11) | 30644.13 | 48.02(37.33-59.55) | 4.72(0.15-26.34) | −66.3 | −9.66(−10.56-−8.76) |
| Netherlands | 3.66(2.54-4.98) | 21.39 | 2.3(0.42-4.22) | 3.16(1.69-4.68) | −14.7 | −2.21(−4.28-−0.1) |
| New Zealand | 1.74(1.06-2.59) | 113.86 | 5.14(4.76-5.53) | 3.69(1.79-5.59) | 117.62 | 5.05(4.17-5.94) |
| Nicaragua | 9.52(5.87-12.51) | 775.46 | 14.47(12.6-16.38) | 22.27(15.74-28.97) | 151.31 | 4.64(2.76-6.56) |
| Niger | 38.91(27.71-53.05) | 30.69 | −4.22(−6.52-−1.86) | 10.42(4.23-19.92) | −58.04 | −8.34(−8.62-−8.05) |
| Nigeria | 127.39(97.33-171.4) | 94.4 | −0.33(−2.51-1.89) | 78.16(61.18-102.06) | −8.5 | −3.17(−3.35-−2.99) |
| Niue | 6.4(0.49-29.7) | 277.35 | 13.09(12.18-14) | 25.9(1.09-105.92) | 290.7 | 9.38(9-9.76) |
| North Macedonia | 0.33(0.27-0.4) | 331.06 | 10.17(9.18-11.17) | 0.37(0.28-0.5) | 6.11 | 1.17(0.72-1.61) |
| Northern Mariana Islands | 2.74(1-6.1) | 132.97 | 5.64(4.58-6.71) | 3.05(1.19-6.19) | −30.37 | 0.47(−0.48-1.43) |
| Norway | 4.35(2.75-6.4) | −32.79 | −2.83(−4.67-−0.95) | 5.29(3.05-7.87) | 53.93 | 2.13(1.37-2.9) |
| Oman | 4.61(2.69-7.01) | 463.21 | 14.24(10.95-17.62) | 4.42(2.84-6.74) | 144.75 | 0.95(−1.15-3.08) |
| Pakistan | 1.24(0.07-5.85) | 117.04 | 4.54(1.11-8.09) | 5.4(0.69-26.69) | 461.24 | 9.01(6.75-11.32) |
| Palau | 6.32(0.49-30.42) | 555.03 | 12.55(12.05-13.05) | 24.78(1.07-102.27) | 202.28 | 9.13(8.81-9.44) |
| Palestine | 0.39(0.35-0.48) | 357.66 | 6.49(5.84-7.14) | 0.42(0.29-0.63) | 51.77 | 1.08(0.64-1.52) |
| Panama | 25.07(23.09-27.33) | 4.96 | −3.72(−5.69-−1.71) | 43.9(34.49-59.21) | 128.4 | 4.96(4.36-5.55) |
| Papua New Guinea | 98.95(1.83-266.58) | 7024.35 | 29.98(19.39-41.5) | 71.08(0.31-245.84) | 19.84 | −1.38(−1.61-−1.15) |
| Paraguay | 18.55(12.49-24.25) | 665.23 | 14.85(13.77-15.93) | 18.5(12.79-23.72) | 20.2 | −1.01(−1.86-−0.16) |
| Peru | 16.12(12-19.33) | 248.52 | 6.86(4.8-8.97) | 17.67(12.71-27.49) | 38.97 | 0.36(−0.74-1.47) |
| Philippines | 10.88(8.42-14.56) | -2.7 | 5.79(1.55-10.2) | 45.13(25.45-72.06) | 405.75 | 10.57(9.78-11.38) |
| Poland | 1.87(1.22-3) | 151.62 | 4.43(2.12-6.79) | 1.92(1.02-3.58) | −1.19 | 0.85(0.37-1.33) |
| Portugal | 9.63(7.6-12.42) | −45.12 | −7.46(−10.03-−4.82) | 19.97(13.89-28.57) | 66.41 | 7.37(5.95-8.82) |
| Puerto Rico | 9.38(5.9-12.94) | −81.54 | −10.99(−12.78-−9.16) | 20.73(13.52-25.9) | 116.02 | 7.91(6.97-8.86) |
| Qatar | 0.26(0.18-0.35) | −61.73 | −8.51(−10.22-−6.76) | 0.2(0.16-0.24) | 260.55 | −0.67(−1.15-−0.18) |
| Republic of Korea | 3.95(2.12-6.63) | 523.92 | 16.63(11.89-21.57) | 2.08(0.77-3.65) | −33.92 | −3.5(−3.99-−3.01) |
| Republic of Moldova | 10.42(7.8-13.92) | 187.25 | 8.6(3.99-13.41) | 15.48(11.46-21.77) | 24.7 | 1.71(1.05-2.38) |
| Romania | 4.79(4-5.6) | 293.51 | 9.69(7.19-12.25) | 4.4(3.63-5.49) | −25.51 | 0.57(−0.47-1.62) |
| Russian Federation | 15.88(12.06-21.85) | 247.04 | 13.21(9.26-17.31) | 78.99(64.56-99.72) | 353.15 | 12.73(10.24-15.28) |
| Rwanda | 201.23(161.99-256.43) | −24.58 | −5.87(−8.22-−3.47) | 41.75(23.74-68.04) | −66.36 | −10.08(−10.41-−9.76) |
| Saint Kitts and Nevis | 47.6(13.86-113.49) | 97.14 | 3(2.45-3.55) | 97.79(22.89-241.88) | 150.81 | 5.37(4.98-5.77) |
| Saint Lucia | 10.35(6.69-12.97) | −10.12 | −2.67(−3.19-−2.15) | 13.46(10.65-17.55) | 54.51 | 2.71(1.34-4.1) |
| Saint Vincent and the Grenadines | 21.6(17.46-24.64) | −50.66 | −6.69(−7.87-−5.49) | 33.82(27.9-41.54) | 67.71 | 3.45(2.24-4.68) |
| Samoa | 6.8(0.1-41.92) | 42.46 | 0.79(−0.78-2.39) | 17.86(0.24-123.19) | 192.95 | 6.68(6.13-7.22) |
| San Marino | 3.2(0.62-10.02) | −4.35 | 0.04(−3.01-3.19) | 4.29(0.75-16.02) | 40.77 | 2.07(1.59-2.56) |
| Sao Tome and Principe | 1.37(0.52-2.65) | −10.86 | −2.52(−4.89-−0.09) | 0.81(0.23-1.62) | −9.32 | −1.93(−3.87-0.04) |
| Saudi Arabia | 2.68(1.51-7.03) | 168.14 | 5.39(3.87-6.94) | 2.46(0.89-6.52) | 71.54 | 0.34(−0.08-0.76) |
| Senegal | 51.15(37.7-70.31) | 79.78 | −0.73(−3.09-1.7) | 16.94(9.39-29.01) | −53.62 | −7.04(−7.44-−6.63) |
| Serbia | 3.58(2.34-4.9) | 549.43 | 19.77(13.53-26.37) | 1.15(0.79-1.79) | −62.13 | −5.32(−6.21-−4.42) |
| Seychelles | 8.43(7.54-9.55) | −21.57 | −3.32(−5.53-−1.05) | 5.65(4.89-6.84) | −24.33 | −3.51(−4.08-−2.94) |
| Sierra Leone | 115.07(88.68-150.78) | 308.97 | 6.25(3.78-8.78) | 93.04(47.5-172.04) | 21.73 | −1.23(−1.64-−0.83) |
| Singapore | 9.35(5.68-15.67) | −44.24 | −10.27(−12.78-−7.68) | 8.15(4.8-13.84) | 24.13 | −0.17(−0.73-0.39) |
| Slovakia | 0.24(0.17-0.3) | 34.21 | 2.74(1.66-3.84) | 0.37(0.27-0.54) | 14.32 | 0.02(−0.92-0.96) |
| Slovenia | 0.33(0.19-0.51) | −11.96 | −2.91(−5.96-0.23) | 0.65(0.41-1.14) | 68.71 | 2.97(0.8-5.19) |
| Solomon Islands | 6.74(0.1-40.77) | 78.8 | 0.34(−1.08-1.77) | 16.38(0.23-116.66) | 176.35 | 6.26(5.75-6.76) |
| Somalia | 46.39(27.05-74.35) | 489.74 | 4.34(−1.93-11.02) | 15.95(7.9-28.89) | −42.3 | −6.8(−6.89-−6.7) |
| South Africa | 1157.98(1001.54-1377.41) | 838.96 | 12.38(6.7-18.36) | 599.28(467.77-760.41) | −35.24 | −4.01(−4.39-−3.61) |
| South Sudan | 127.42(54.45-240.6) | 191.75 | 3.82(1.15-6.57) | 121.41(19.54-364.51) | −3.61 | 0.16(−0.05-0.38) |
| Spain | 2.98(2.36-4.27) | −80.33 | −13.51(−16.1-−10.84) | 10.51(6.93-15.1) | 207.48 | 10.5(8.99-12.02) |
| Sri Lanka | 0.45(0.34-0.72) | −13.9 | −4.05(−5.04-−3.05) | 1(0.65-1.42) | 123.03 | 6.04(5.16-6.92) |
| Sudan | 28.3(11.91-54.97) | 212.7 | 4.28(2.37-6.21) | 36.46(5.5-143.6) | 82.17 | 2.13(1.94-2.32) |
| Suriname | 37.51(31.81-43.21) | 35.06 | 1.41(−0.18-3.03) | 40.99(34.18-51.49) | 25.19 | 1.58(1.1-2.06) |
| Sweden | 1.29(0.7-2) | −64.14 | −5.16(−8.97-−1.19) | 2.53(1.33-3.71) | 82.55 | 3.87(2.93-4.82) |
| Switzerland | 5.84(3.67-7.91) | −55.56 | −8.44(−10.99-−5.83) | 6.35(2.91-9.58) | 40.52 | 0.86(0.28-1.44) |
| Syrian Arab Republic | 0.17(0.09-0.22) | 87.35 | 0.77(−0.36-1.91) | 0.21(0.14-0.31) | 2.52 | 0.68(−2.39-3.83) |
| Taiwan  (Province of China) | 7(4.46-10.28) | 422.82 | 10.02(8.19-11.88) | 6.37(3.09-10.45) | −18.48 | −1.97(−3.45-−0.46) |
| Tajikistan | 2.3(1.72-2.94) | 86.59 | 1.57(−1.11-4.32) | 5.61(2.72-8.16) | 238.56 | 7.62(7.02-8.21) |
| Thailand | 47.11(23.55-66.53) | −54.79 | −9.65(−12.89-−6.28) | 40.34(31.4-55.07) | −18.08 | −1.99(−2.64-−1.34) |
| Timor-Leste | 40.73(0.75-238.68) | −51.42 | −8.32(−11.7-−4.82) | 25.01(0.35-165.11) | −10.25 | −3.97(−4.3-−3.64) |
| Togo | 218.71(151.62-308.64) | 118.43 | −0.93(−5.02-3.34) | 54.95(31.14-93.72) | −62.16 | −8.92(−9.16-−8.67) |
| Tokelau | 6.72(0.51-31.2) | 322.45 | 12.87(12.13-13.61) | 26.85(1.13-110.98) | 305.44 | 9.25(9-9.51) |
| Tonga | 3.19(1.24-6.74) | 70.65 | 3.8(2.54-5.07) | 4.69(1.68-10.49) | 32.89 | 2.28(1.82-2.75) |
| Trinidad and Tobago | 32.69(27.72-39.6) | 2.03 | −2.17(−4.14-−0.15) | 32.65(27.5-39.24) | −8.04 | 0.11(−0.62-0.84) |
| Tunisia | 1.9(0.23-8.73) | 452.99 | 11.44(11.02-11.86) | 2.6(0.37-11.01) | 36.11 | 2.01(1.7-2.32) |
| Turkey | 0.48(0.4-0.56) | 834.74 | 12.55(10.6-14.52) | 0.54(0.39-0.76) | 11.3 | −0.03(−0.38-0.32) |
| Turkmenistan | 7.3(5.86-8.52) | −14.87 | 0.37(−1.53-2.31) | 4.8(3.79-5.92) | −3.25 | 0.97(−1.34-3.33) |
| Tuvalu | 6.53(0.5-30.39) | 417.88 | 13.04(12.2-13.89) | 26.45(1.09-110.82) | 347.05 | 9.39(9.18-9.6) |
| Uganda | 446.31(351.16-564.86) | −15.38 | −4.04(−4.16-−3.92) | 185.42(103.9-305.14) | −42.49 | −6.23(−6.98-−5.47) |
| Ukraine | 31.22(28.17-35.21) | 260.25 | 13.08(10.35-15.87) | 70.11(51.81-94.24) | 157 | 12.18(8.94-15.51) |
| United Arab Emirates | 1.46(0.1-6.79) | 501.86 | 9.02(7.6-10.46) | 3.76(0.09-23.27) | 182.95 | 1.33(−7.32-10.79) |
| United Kingdom | 9.03(5.98-12.1) | 160.88 | 9.79(7.64-11.98) | 8.33(4.83-12.14) | 3.59 | 0.03(−0.34-0.39) |
| United Republic of Tanzania | 379.79(289.06-492.97) | −37.61 | −6.98(−7.27-−6.69) | 110.29(56.94-183.57) | −57.98 | −8.07(−8.5-−7.64) |
| United States of America | 15.07(8.95-21.03) | −25.17 | −1.17(−3.02-0.71) | 20.87(9.09-32.04) | 56.1 | 2.95(2.53-3.36) |
| United States Virgin Islands | 11.99(9.93-14.24) | −45.48 | −4(−4.38-−3.62) | 19.37(14.65-24.85) | 52.93 | 4.01(3.23-4.79) |
| Uruguay | 16.42(12.11-22.74) | 45.01 | 2.8(1.81-3.81) | 26.03(18.28-34.23) | 68.06 | 6.02(4.01-8.07) |
| Uzbekistan | 4.72(3.63-5.61) | 345.3 | 7.81(4.81-10.89) | 6.85(2.26-11.23) | 103.24 | 7.99(4.55-11.53) |
| Vanuatu | 9.41(0.12-62.41) | 89.96 | 2.83(0.28-5.44) | 17.17(0.23-119.55) | 194.99 | 2.63(−0.94-6.34) |
| Venezuela  (Bolivarian Republic of) | 17.97(15.83-20.68) | 166.22 | 3.12(1.48-4.78) | 21.99(16.93-27.29) | 32.8 | 2.34(1.62-3.06) |
| Viet Nam | 14.95(13.07-17.33) | 274.3 | 9.4(7.06-11.8) | 14.59(11.91-18.94) | 3.52 | −0.3(−0.99-0.4) |
| Yemen | 1.91(0.14-8.1) | 111.85 | 1.96(1.59-2.33) | 2.94(0.12-13.11) | 126.21 | 3.95(3.15-4.75) |
| Zambia | 779.83(638.89-981.17) | −19.32 | −5.33(−5.8-−4.86) | 274.57(155.95-437.92) | −39.58 | −6.74(−6.91-−6.57) |
| Zimbabwe | 915.95(744.84-1159.55) | −18.74 | −6.35(−8.54-−4.1) | 213.63(132.11-326.91) | −70.31 | −9.6(−10.18-−9.02) |

EAPC: estimated annual percentage change; ASR, age-standardized rate; CI, confidence interval; UI: uncertainty interval.

**Supplementary table 7**. Age-standardized rate of HIV/AIDS prevalence at the national level and for both sexes in 2019, and the percentage changes and EAPCs in HIV/AIDS prevalence from 1990 to 2019.

|  | **1990-2004** | | | **2005-2019** | | |
| --- | --- | --- | --- | --- | --- | --- |
| **Characteristics** | ASR/100,000  (95% UI) | Change in  number (%) | EAPC  (95%CI) | ASR/100,000  (95% UI) | Change in  number (%) | EAPC  (95%CI) |
| Afghanistan | 8.25(0.84-32.15) | 243.82 | 3.23(2.94-3.52) | 16.63(1.56-64.16) | 233.92 | 5.02(4.19-5.86) |
| Albania | 1.03(0.78-1.46) | 29.87 | 1.47(1.05-1.9) | 1.12(0.68-1.81) | 0.98 | 0.36(0.05-0.67) |
| Algeria | 10.62(1.79-48.95) | 408.39 | 9.16(8.93-9.4) | 22.07(3.61-75.47) | 153.76 | 4.77(4.24-5.31) |
| American Samoa | 13.45(7.37-22.9) | 177.51 | 5.02(4.69-5.35) | 26.69(12.76-52.43) | 74.61 | 4.22(3.93-4.51) |
| Andorra | 84.42(7.02-332.7) | 68.75 | 0.56(0.21-0.91) | 107.37(6.45-439.16) | 28.08 | 1.76(1.53-1.98) |
| Angola | 949.32(601.18-1392.74) | 1740.25 | 18.56(15.88-21.3) | 1699.88(1162.27-2347.38) | 167.54 | 4.05(3.97-4.13) |
| Antigua and Barbuda | 179.19(136.12-229.22) | 101.91 | 1.81(0.83-2.81) | 279.35(240.53-320.52) | 79.09 | 4.07(3.4-4.74) |
| Argentina | 247.84(149.83-360.81) | 169.6 | 5.56(4.95-6.18) | 395.33(229.43-598.12) | 92.54 | 3.02(2.88-3.16) |
| Armenia | 5.46(3.56-8.88) | 338.71 | 10.38(7.39-13.45) | 39.74(31.61-53.04) | 559.66 | 15.49(14.33-16.67) |
| Australia | 49.79(33.3-65.78) | 25.38 | −0.11(−0.49-0.26) | 56.49(35.5-79.28) | 44.25 | 0.79(0.7-0.87) |
| Austria | 91.78(53.91-135.93) | 200.27 | 6.08(5.41-6.75) | 158.1(89.74-231.08) | 82.58 | 3.64(3.43-3.84) |
| Azerbaijan | 9.64(7.82-11.92) | 131.92 | 4.66(3.78-5.55) | 27.35(20.45-37.71) | 268.91 | 9.1(8.08-10.14) |
| Bahamas | 703(545.4-944.18) | 63.14 | 0.4(−0.91-1.73) | 951.81(817.28-1160.65) | 60.9 | 2.35(2.13-2.56) |
| Bahrain | 14.68(11.84-17.88) | 83.78 | 0.12(−0.85-1.11) | 12.64(10.75-15.39) | 68.01 | −0.92(−1.26-−0.57) |
| Bangladesh | 3.33(2.12-7.33) | 1532.56 | 31.82(27.43-36.36) | 5.05(2.25-18.68) | 67.03 | 1.86(0.69-3.04) |
| Barbados | 274.82(222.86-341.18) | 75.7 | 2.85(2.06-3.64) | 432.02(371.05-519.98) | 74.3 | 2.83(2.76-2.9) |
| Belarus | 42.04(26.54-62.39) | 352.96 | 11.21(10.51-11.92) | 158.73(116.12-212.69) | 257.09 | 9.25(8.52-9.98) |
| Belgium | 111.62(61.42-164.27) | 350.27 | 9.67(8.15-11.21) | 162.27(89.97-243.02) | 62.59 | 2.45(2.37-2.53) |
| Belize | 519(372.03-694.13) | 374.41 | 7.66(7.45-7.87) | 651.02(548.23-789.99) | 91.71 | 1.65(1.29-2.01) |
| Benin | 1023.31(829.04-1239.36) | 2633.54 | 17.88(10.78-25.44) | 795.49(656.96-951.86) | 16.77 | −1.02(−1.33-−0.71) |
| Bermuda | 186.97(138.33-247.7) | −3.78 | −1.21(−2.08-−0.32) | 259.38(225.74-308.96) | 32.39 | 3.07(2.5-3.65) |
| Bhutan | 46.21(4.27-195.27) | 145.7 | 4.07(3.81-4.33) | 88.61(6.24-412.76) | 134.22 | 4.49(4.34-4.64) |
| Bolivia  (Plurinational State of) | 88.46(24.18-261.48) | 78.49 | 0.43(−0.35-1.21) | 140.27(28.11-486.21) | 119.13 | 3.7(3.53-3.88) |
| Bosnia and Herzegovina | 1.07(0.87-1.29) | −22.86 | −1.91(−2.35-−1.47) | 3.02(2.37-3.75) | 177.52 | 8.47(8.01-8.94) |
| Botswana | 16671.79(15763.02-17675.76) | 550.39 | 9.79(6.68-12.99) | 15687.15(13849.42-17675.35) | 27.56 | −0.37(−0.52-−0.22) |
| Brazil | 248.41(206.52-280.09) | 178.7 | 5.85(5.56-6.14) | 366.46(313.83-430.32) | 77.84 | 2.43(2.34-2.53) |
| Brunei Darussalam | 46.11(26.12-72.35) | 418.37 | 8.9(8.48-9.32) | 105.59(57.41-169.95) | 193.8 | 5.68(5.61-5.76) |
| Bulgaria | 13.68(10.04-18.88) | 186.19 | 7.67(6.52-8.82) | 23.96(20.21-27.7) | 47.37 | 3.61(2.91-4.33) |
| Burkina Faso | 1367.13(1128.93-1674.94) | −35.51 | −6.21(−6.93-−5.5) | 585.2(503.97-696.56) | −30.92 | −5.1(−5.35-−4.86) |
| Burundi | 2886.38(2447.51-3419.93) | −1.74 | −2.4(−4.21-−0.55) | 1030.5(900.84-1183.58) | −41.46 | −6.44(−6.96-−5.92) |
| Cabo Verde | 605.34(312.1-1003.72) | 198.73 | 4.18(2.35-6.05) | 504.86(289.85-862.11) | 16.13 | −1.05(−1.12-−0.99) |
| Cambodia | 834.76(577.86-1143.22) | 7390.02 | 26.44(14.79-39.27) | 463.3(336.86-581.89) | −22.03 | −3.37(−3.53-−3.2) |
| Cameroon | 3648.47(3277.53-4033.61) | 1343.67 | 15.8(12.03-19.69) | 2560.94(2318.52-2809.59) | 11.09 | −2.53(−2.82-−2.25) |
| Canada | 154.82(104.79-210.55) | 56.02 | 1.74(1.6-1.88) | 203.08(130.82-291.3) | 53.24 | 1.88(1.69-2.08) |
| Central African Republic | 4707.57(3919.99-5649.99) | 122.9 | 1.29(−0.82-3.45) | 2764.82(2148.87-3583.35) | −23.4 | −3.46(−3.56-−3.35) |
| Chad | 1369.81(926.85-1892.61) | 356.96 | 7.36(5.13-9.65) | 1288.2(1013.31-1633.63) | 40.11 | 0.09(−0.15-0.33) |
| Chile | 117.09(72.15-173.58) | 203.4 | 6.43(6.1-6.75) | 271.94(158.17-424.8) | 154.43 | 5.52(5.33-5.71) |
| China | 21.95(12.93-35.99) | 398.42 | 9.82(9.21-10.43) | 31.72(16.48-59.31) | 47.29 | 1.46(0.54-2.38) |
| Colombia | 92.69(67.13-128.67) | 405.52 | 9.92(7.82-12.06) | 200.47(157.58-256.4) | 154.38 | 5.59(5.3-5.87) |
| Comoros | 15.34(11.22-30.17) | 385.5 | 5.73(4.83-6.64) | 17.74(10.16-57.95) | 45.87 | 0.51(−0.62-1.66) |
| Congo | 2474.35(1908.87-3159.77) | 90.59 | 0.43(−0.93-1.8) | 2004.76(1380.65-2977.2) | 24.41 | −1.11(−1.24-−0.97) |
| Cook Islands | 34.54(4.61-159.51) | 483.89 | 12.87(12.72-13.03) | 157.4(10.21-635.44) | 277.7 | 10.65(10.5-10.81) |
| Costa Rica | 71.14(55.78-92.28) | 155.03 | 3.61(3.27-3.94) | 129.7(105.72-163.45) | 114.89 | 4.05(3.8-4.3) |
| Croatia | 8.41(6.53-10.25) | 181.98 | 7.42(6.32-8.54) | 16.57(13.12-21.23) | 95.65 | 4.47(3.88-5.05) |
| Cuba | 32.28(19.23-47.04) | 289.47 | 8.45(7.55-9.36) | 181.29(111.27-268.48) | 394.41 | 12.02(11.04-13.02) |
| Cyprus | 7.93(5.2-11.19) | 245.86 | 6.88(6.22-7.54) | 12.44(7.19-19.67) | 106.47 | 3.24(3.06-3.42) |
| Czechia | 5.23(3.77-6.96) | 438.9 | 12.34(12.14-12.54) | 12.94(9.25-18.34) | 139.07 | 6.04(5.62-6.46) |
| Cote d'Ivoire | 4404.59(3752.84-5171.05) | 166.56 | 2.78(0.22-5.4) | 2071.43(1843.92-2319.03) | −33.92 | −4.92(−5.07-−4.78) |
| Democratic People's Republic of Korea | 29.91(6.19-164.95) | 716.97 | 13.5(12.83-14.18) | 45.38(7.23-265.81) | 60.68 | 2.4(1.95-2.84) |
| Democratic Republic of  the Congo | 1228.5(981.66-1482.82) | 51.97 | −0.19(−1.44-1.07) | 576.06(470.16-707.67) | −28.66 | −5.04(−5.37-−4.7) |
| Denmark | 68.58(49.17-94.2) | 77.86 | 2.85(2.4-3.31) | 91.37(61.66-127.01) | 44.32 | 2.09(1.99-2.2) |
| Djibouti | 1924.22(1325.26-2630.46) | 20418.78 | 37.83(27.28-49.25) | 1137.32(622.81-2015.58) | 2.11 | −3.64(−3.77-−3.51) |
| Dominica | 178.43(134.76-225.23) | 61.34 | 2.86(1.91-3.82) | 295.95(252.76-353.47) | 73.05 | 4.17(3.84-4.51) |
| Dominican Republic | 594.58(513.27-692.15) | 232.46 | 4.46(1.67-7.32) | 541.14(417.72-697.25) | 15.18 | −0.35(−0.42-−0.28) |
| Ecuador | 100.04(74.01-134.06) | 443.7 | 10.56(10.16-10.97) | 221.06(167.12-301.23) | 170.05 | 5.46(5.34-5.58) |
| Egypt | 1.95(1.62-2.42) | 32.86 | −1.24(−1.52-−0.95) | 4.76(3.95-5.54) | 213.63 | 6.97(6.05-7.89) |
| El Salvador | 240.4(182.07-287.94) | 589.26 | 13.67(12.18-15.19) | 339.65(279.56-401) | 53.41 | 2.11(1.42-2.81) |
| Equatorial Guinea | 2967.79(2014.07-4235.26) | 2300.83 | 20.04(17.41-22.72) | 6408.12(4177.45-9505.96) | 265.06 | 5.17(5.02-5.32) |
| Eritrea | 1178.73(795.59-1613.97) | 238.24 | 4.19(1.21-7.25) | 472.09(353.15-632.43) | −42.03 | −5.7(−5.87-−5.52) |
| Estonia | 60.56(42.45-79.8) | 2946.65 | 31.45(28.57-34.39) | 241.08(206.51-277.54) | 237.52 | 8.82(7.82-9.83) |
| Eswatini | 18866.96(17109.47-20707.61) | 7871.84 | 30.97(23.37-39.04) | 22031.68(20432.73-23878.5) | 23.91 | 0.95(0.66-1.25) |
| Ethiopia | 2120.42(1828.53-2440.35) | 213.69 | 4.57(1.33-7.91) | 1031.62(929.41-1135.86) | −29.44 | −4.27(−4.5-−4.04) |
| Fiji | 23.32(18.46-29.43) | 202.29 | 6.8(6.08-7.53) | 50.86(42.56-60.78) | 122.39 | 5.6(5.42-5.79) |
| Finland | 10.56(7.28-14.69) | 108.28 | 4.7(4.53-4.86) | 20.19(12.96-28.27) | 101.68 | 4.33(4.09-4.58) |
| France | 110.79(77.97-146.22) | 12.1 | −0.51(−0.77-−0.24) | 122.73(78.22-172.31) | 28.89 | 0.66(0.64-0.67) |
| Gabon | 2826.65(1864.17-4057.17) | 671.19 | 12.03(9.15-14.98) | 3142(2454.92-4087.02) | 45.3 | 0.72(0.61-0.83) |
| Gambia | 1382.37(858.82-1963.72) | 1734.27 | 19.19(15.84-22.64) | 1470.19(981.57-2088.82) | 47.05 | 0.39(0.3-0.48) |
| Georgia | 8.94(4.18-16.71) | 314.03 | 12.24(9.74-14.79) | 104.47(68.61-167.83) | 791.25 | 17.14(14.11-20.25) |
| Germany | 60.55(42.47-79.87) | 39.21 | 1.34(1.23-1.45) | 71.67(49.31-98.89) | 26.46 | 1.07(1.02-1.13) |
| Ghana | 1650.91(1415.35-1901.68) | 363.85 | 7.96(5.75-10.21) | 1115.68(947.34-1329.92) | 0.18 | −2.52(−2.59-−2.45) |
| Greece | 17.8(14.26-22.95) | 73.43 | 2.61(2.26-2.96) | 38.4(28.79-56.64) | 84.67 | 5.35(4.83-5.88) |
| Greenland | 217.65(116.67-349.11) | 34.22 | 1.74(1.61-1.87) | 295.39(149.96-479.05) | 31.7 | 1.92(1.68-2.16) |
| Grenada | 144.8(110.18-180.5) | 126.11 | 2.51(1.65-3.37) | 226.37(188.67-275.36) | 56.76 | 3.79(3.47-4.11) |
| Guam | 39.5(22.54-67.03) | 67.47 | 1.68(1.26-2.1) | 69.44(30.7-145.24) | 67.79 | 3.59(3.45-3.73) |
| Guatemala | 143.11(90.14-206.77) | 343.41 | 7.86(7.27-8.45) | 184.73(130.8-283.72) | 89.71 | 1.25(1.08-1.42) |
| Guinea | 1056.11(810.03-1317.21) | 797.9 | 14.12(11.74-16.55) | 1350.17(991.98-1805.7) | 69.23 | 1.79(1.63-1.94) |
| Guinea-Bissau | 2233.81(1357.49-3314.92) | 1017.5 | 15.89(12.91-18.95) | 2139.69(1264.96-3261.74) | 26.32 | −0.28(−0.42-−0.15) |
| Guyana | 786.32(546.58-1006.51) | 287.87 | 10.16(9.08-11.25) | 1232.09(1051.24-1437.96) | 53.68 | 2.95(2.58-3.31) |
| Haiti | 2138.44(1773.48-2563.52) | 140.75 | 2.43(0.46-4.44) | 1475.86(1210.82-1812.76) | 0.25 | −2.28(−2.34-−2.22) |
| Honduras | 59.26(45.45-71.86) | 94.47 | 1.23(0.55-1.91) | 109.51(91.08-133.13) | 135.66 | 3.41(2.85-3.97) |
| Hungary | 10.94(9.58-12.4) | −45.69 | −4.89(−5.47-−4.3) | 15.53(12.73-17.82) | 52.93 | 2.49(1.63-3.36) |
| Iceland | 31.55(26.61-37.08) | 89.56 | 2.94(2.38-3.5) | 50.83(41.17-68.12) | 82.52 | 2.77(2.57-2.96) |
| India | 197.97(181.06-215.74) | 3997.8 | 25.98(19.52-32.79) | 127.95(114.43-143.25) | −15.24 | −2.71(−3.32-−2.09) |
| Indonesia | 94.75(83.05-107.27) | 17395.9 | 20.5(8.5-33.81) | 104.27(90.42-121.95) | 18.52 | 0.25(0.03-0.46) |
| Iran  (Islamic Republic of) | 9.46(7.23-12.21) | 319.75 | 7.15(6.33-7.97) | 28.54(21.84-35.22) | 263.11 | 7.91(7.17-8.66) |
| Iraq | 2.67(1.44-5.83) | 704.55 | 11.35(11.07-11.63) | 5.76(2.91-12.41) | 211.56 | 5.13(5.08-5.19) |
| Ireland | 22.35(14.8-32.3) | 204.29 | 4.58(3.24-5.93) | 41.5(25.23-61.61) | 103.2 | 4.26(3.97-4.55) |
| Israel | 70.3(38.25-103.82) | 345.39 | 7.74(7.26-8.21) | 139.18(77.21-200.06) | 151.95 | 4.35(3.95-4.75) |
| Italy | 92.63(62.69-127.29) | 29.26 | −0.03(−0.56-0.5) | 124.08(83.72-173.67) | 50.18 | 2.13(1.96-2.31) |
| Jamaica | 295.32(204.59-421.29) | 173.19 | 4.99(3.46-6.55) | 524.65(422.94-650.27) | 99.85 | 4.45(4.31-4.58) |
| Japan | 10.01(4.74-16.24) | 302.86 | 9.34(8.85-9.83) | 24.56(13.74-36.83) | 158.85 | 6.48(6.25-6.71) |
| Jordan | 3.41(2.49-4.27) | 516.55 | 10.52(10.09-10.95) | 5.73(4.76-7.14) | 220.22 | 3.06(2.33-3.8) |
| Kazakhstan | 26.34(19.02-37.42) | 85.5 | 4.18(3.11-5.26) | 81.97(64.94-104.87) | 282.05 | 8.55(7.14-9.99) |
| Kenya | 6714.59(6017.79-7488.03) | 196.11 | 3.44(0.93-6.01) | 4235.34(3733.04-4761.28) | −4.43 | −2.76(−2.89-−2.63) |
| Kiribati | 22.11(17.79-27.32) | 42.85 | 0.34(0.16-0.53) | 34.06(25.62-45.19) | 88.8 | 3.39(3.16-3.63) |
| Kuwait | 5.24(4.56-5.84) | 104.08 | 3.79(3.47-4.11) | 5.77(4.91-6.64) | 124.35 | 0.53(0.49-0.57) |
| Kyrgyzstan | 32.62(23.14-48.21) | 392.61 | 10.05(9.19-10.92) | 96.81(69.75-137.79) | 239.79 | 6.81(6.38-7.25) |
| Lao People's Democratic Republic | 128.54(54.77-492.62) | 172663.27 | 44.12(30.42-59.26) | 124.96(44.79-521.02) | 21.19 | −1.43(−2.18-−0.68) |
| Latvia | 59.22(39.34-89.23) | 126.39 | 5.24(3.77-6.73) | 188.92(150.7-236.83) | 153.07 | 7.69(7.44-7.93) |
| Lebanon | 19.6(1.89-78.76) | 21.88 | −0.93(−1.39-−0.47) | 22.46(2.55-76.66) | 60.56 | 0.88(0.44-1.32) |
| Lesotho | 16371.65(15121.88-17687.98) | 1167.41 | 18.09(14.36-21.93) | 19532.09(18410.2-20844.04) | 31.95 | 1.28(1.11-1.44) |
| Liberia | 1253.52(892.82-1648.94) | 956.62 | 13.52(10.22-16.92) | 930.02(675.98-1314.81) | 17.44 | −2.02(−2.09-−1.94) |
| Libya | 13.13(3.81-52.88) | 303.05 | 6.84(6.59-7.09) | 19.37(2.94-89.38) | 85.61 | 2.44(2.04-2.83) |
| Lithuania | 31.1(20.94-43.13) | 238.3 | 9.34(8.87-9.81) | 63.26(42.59-82.55) | 59.86 | 4.5(4.16-4.85) |
| Luxembourg | 56.55(36.1-87.3) | 157.39 | 4.7(3.48-5.92) | 72.33(43.91-110.16) | 75.8 | 1.72(1.49-1.95) |
| Madagascar | 117.42(53.72-209.21) | 22921.41 | 43.37(35.79-51.38) | 186.79(92.33-325.37) | 128.22 | 2.84(2.77-2.91) |
| Malawi | 9445.07(8640.06-10355.38) | 141.45 | 4.15(2.31-6.02) | 7818.28(7299.41-8379.99) | 14.39 | −0.79(−0.99-−0.59) |
| Malaysia | 127.88(97.8-162.22) | 709.43 | 8.67(5.62-11.81) | 165.65(140.59-191.45) | 57.32 | 1.19(0.94-1.45) |
| Maldives | 3.59(3.05-4.03) | 208.16 | 5.09(4.93-5.25) | 3.12(2.47-4.18) | 58.64 | −1.2(−1.46-−0.95) |
| Mali | 997.1(801.78-1227.63) | 514.55 | 10.37(7.54-13.28) | 743.43(558.47-1001.32) | 16.13 | −1.87(−2.14-−1.59) |
| Malta | 32.37(20.66-43.62) | 140.48 | 5.27(5.04-5.51) | 80.75(47.93-117.99) | 162.37 | 6.2(5.99-6.43) |
| Marshall Islands | 46.34(3.37-304.16) | 133.02 | 2.34(1.72-2.98) | 126.86(4.19-830.68) | 205.03 | 7.3(7.13-7.46) |
| Mauritania | 74.03(50.24-177.09) | −15.99 | −4.96(−5.37-−4.54) | 40.27(17.37-161.19) | −21.96 | −3.49(−4.27-−2.71) |
| Mauritius | 49.41(26.41-91.98) | 960.14 | 19.77(16.06-23.61) | 230.4(193.13-291.8) | 320.81 | 11.11(9.76-12.48) |
| Mexico | 91.68(67.82-122.73) | 177.7 | 2.84(1.3-4.4) | 167.37(135.39-206.36) | 114.39 | 4.2(4.13-4.28) |
| Micronesia  (Federated States of) | 129.45(9.94-816.27) | 244.63 | 5.3(3.62-7) | 949.69(26.83-6909.46) | 481.19 | 13.49(11.77-15.24) |
| Monaco | 44.85(12.86-115.82) | 31.91 | 0.12(−0.22-0.47) | 64.13(12.74-199.49) | 47.15 | 2.49(2.41-2.57) |
| Mongolia | 2.98(1.68-5.14) | 18620.13 | 24.14(11.71-37.95) | 12.68(4.88-25) | 468.69 | 11.88(10.82-12.95) |
| Montenegro | 7.17(5.48-8.68) | 258.89 | 9.2(7.95-10.46) | 12.75(9.9-16.64) | 73.73 | 3.89(3.82-3.97) |
| Morocco | 34.61(7.56-139.22) | 390.62 | 9.65(9.05-10.25) | 35.65(3.92-115.46) | 19.82 | −0.36(−0.57-−0.15) |
| Mozambique | 6174.41(5528.93-6902.98) | 1402.43 | 17.24(14.52-20.02) | 10533.23(9209.7-12263.13) | 118.64 | 3.77(3.61-3.94) |
| Myanmar | 475.82(426.67-526.84) | 2926.58 | 20.33(14.88-26.05) | 381.27(343.16-421.14) | −7.18 | −1.82(−2.17-−1.47) |
| Namibia | 9849.81(8747.48-11091.67) | 1537.3 | 18.27(14.03-22.66) | 10105.38(9494.9-10787.74) | 24.81 | 0.42(0.12-0.71) |
| Nauru | 36.58(7.53-162.34) | 479.64 | 12.75(12.5-13.01) | 151.87(10.5-625.14) | 270.97 | 9.81(9.72-9.89) |
| Nepal | 122.15(14.16-499.96) | 350340.68 | 64.97(52.41-78.57) | 96.29(8.19-371.56) | −10.65 | −2.7(−3.22-−2.18) |
| Netherlands | 75.43(49.25-100.79) | 48.99 | 1.92(1.62-2.23) | 94.22(63.37-123.39) | 37.32 | 1.67(1.43-1.9) |
| New Zealand | 25.4(17.56-35.29) | 34.64 | 0.85(0.5-1.21) | 53.37(31.9-74.78) | 121.47 | 5.39(5.29-5.5) |
| Nicaragua | 55.5(35.69-75.22) | 892.62 | 15.42(14.79-16.05) | 222.4(178.56-268.44) | 354.34 | 9.95(9.01-10.91) |
| Niger | 509.95(395.44-635.86) | 413.13 | 7.65(4.73-10.66) | 282.9(204.66-386.68) | −19.04 | −3.63(−3.8-−3.45) |
| Nigeria | 1348.26(1089.85-1636.4) | 598.11 | 10.55(7.61-13.56) | 1269.29(1135.99-1417.91) | 29.5 | −0.17(−0.33-−0.01) |
| Niue | 36.17(6.06-162.91) | 321.4 | 12.92(12.72-13.13) | 148.68(9.81-591.63) | 294.62 | 9.83(9.74-9.92) |
| North Macedonia | 1.86(1.4-2.28) | 238.15 | 8.36(8.09-8.64) | 4.74(3.7-6.21) | 148.67 | 6.22(5.95-6.48) |
| Northern Mariana Islands | 17.84(9.62-30.45) | 288.15 | 7.37(7.05-7.69) | 33.1(14.51-65.59) | 4.15 | 3.59(3.36-3.82) |
| Norway | 30.2(19.65-43.26) | 155.65 | 5.29(4.26-6.34) | 60.88(39.29-89.23) | 123.98 | 4.98(4.9-5.05) |
| Oman | 42.23(33.1-53.89) | 907.77 | 17.42(16.69-18.14) | 51.48(38.32-67.57) | 197.22 | 0.76(0.53-0.99) |
| Pakistan | 7.86(2.53-26.09) | 58.36 | −0.91(−1.49-−0.33) | 37.05(3.24-182.9) | 542.4 | 11.7(10.92-12.5) |
| Palau | 32.41(4.89-150.84) | 642.25 | 12.3(12.18-12.42) | 141.87(9.04-577.96) | 246.33 | 10.12(9.98-10.27) |
| Palestine | 2.87(2.08-4.06) | 438.43 | 7.99(7.5-8.47) | 3.72(2.15-6.6) | 98.71 | 1.48(1.36-1.61) |
| Panama | 266.65(212.21-337.18) | 236.52 | 4.86(3-6.75) | 497.33(397.08-649.65) | 138.46 | 4.37(4.09-4.65) |
| Papua New Guinea | 864.33(51.11-2334.17) | 39267.81 | 49.43(40.43-59.01) | 1018.17(28.05-2987.56) | 74.79 | 0.98(0.94-1.02) |
| Paraguay | 100.83(72.61-123.3) | 669.02 | 13.63(13.03-14.24) | 228.87(185.01-275.62) | 170.34 | 5.17(4.35-6) |
| Peru | 125.47(92.08-158.33) | 234.65 | 7.47(6.78-8.15) | 236.71(187.75-319.2) | 132.76 | 4.68(4.24-5.11) |
| Philippines | 61.65(40.74-91.83) | 935.59 | 17.49(14.09-21) | 252.48(131.32-439.24) | 409.43 | 9.9(9.57-10.22) |
| Poland | 20.28(12.44-31.96) | 957.91 | 16.76(14.05-19.54) | 34.18(20.65-60.85) | 76.33 | 3.26(3.11-3.4) |
| Portugal | 256.66(202.76-330.84) | 247.32 | 7.66(5.6-9.76) | 320.74(250.07-416.17) | 28.68 | 1.6(1.04-2.15) |
| Puerto Rico | 191.92(139.7-266.5) | −40.98 | −5.24(−6.16-−4.32) | 209.96(172.2-248.86) | 10.48 | 1.5(0.36-2.65) |
| Qatar | 6.73(5.93-7.44) | 79.77 | −0.01(−0.45-0.44) | 4.67(4.16-5.24) | 122.09 | −2.42(−2.7-−2.13) |
| Republic of Korea | 32.48(17.58-54.58) | 579.59 | 13.8(11.83-15.81) | 40.71(24.3-61.75) | 49.67 | 0.89(0.43-1.34) |
| Republic of Moldova | 66.17(43.42-97.13) | 286.93 | 11.21(10.02-12.41) | 184.11(127.39-261.47) | 164.89 | 6.78(6.5-7.06) |
| Romania | 37.38(31.48-42.31) | 683.26 | 16.11(14.88-17.36) | 78.07(67.53-91.61) | 62.88 | 4.67(4.32-5.02) |
| Russian Federation | 107.54(66.96-172.3) | 255.14 | 11.1(9.84-12.37) | 700.01(571.01-877.21) | 538.29 | 14.88(14.29-15.47) |
| Rwanda | 2851.77(2521.04-3235.97) | 212.88 | 5.82(3.07-8.64) | 2169.44(1955.04-2394.77) | 7.82 | −1.47(−1.58-−1.35) |
| Saint Kitts and Nevis | 385.33(120.86-712.69) | 393.15 | 9.04(7.7-10.41) | 740.82(225.5-1655.87) | 136.68 | 4.7(4.49-4.91) |
| Saint Lucia | 125.58(97.79-151.68) | 125.75 | 3.1(2.27-3.95) | 183.17(153.92-235.09) | 73.67 | 3.1(2.83-3.37) |
| Saint Vincent and the Grenadines | 346.47(247.26-476.72) | 100.66 | 3.46(1.75-5.2) | 367.52(305.23-458.05) | 16.93 | 1.06(0.57-1.55) |
| Samoa | 47.3(5.54-301.78) | 105.31 | 2.73(2.13-3.33) | 131.86(7.17-865.29) | 213.05 | 7.35(7.21-7.49) |
| San Marino | 45(12.93-114.91) | 36.59 | −0.26(−0.54-0.03) | 61.65(12.18-188.13) | 51.84 | 2.25(2.17-2.33) |
| Sao Tome and Principe | 55.63(47.63-64.49) | 81.1 | 1.21(0.04-2.4) | 16.71(13.19-26.35) | −52.38 | −6.98(−8.23-−5.71) |
| Saudi Arabia | 22.69(14.35-45.18) | 284.9 | 6.69(6.39-6.99) | 23.98(9.88-59.74) | 96.05 | 0.31(0.07-0.55) |
| Senegal | 559.99(433.94-700.7) | 527.78 | 10.05(7.3-12.88) | 410.18(334.17-508.44) | −3.12 | −1.88(−2.16-−1.6) |
| Serbia | 16.7(11.58-28.87) | 167.96 | 6.98(5.13-8.85) | 23.11(17.56-33.56) | 32.4 | 1.11(0.45-1.78) |
| Seychelles | 76.78(58.63-100.55) | 413.12 | 6.76(4.09-9.48) | 90.82(73.66-119.43) | 41.61 | 1.1(0.75-1.44) |
| Sierra Leone | 919.79(737.44-1107.59) | 969.12 | 15.05(12.44-17.73) | 1199.64(892.86-1627.06) | 90.43 | 1.86(1.78-1.95) |
| Singapore | 62.51(37.9-98.52) | 400.17 | 7.78(4.92-10.72) | 58.88(38.24-87.62) | 47.43 | −0.5(−0.57-−0.42) |
| Slovakia | 1.92(1.38-2.45) | 304.86 | 9.43(8.31-10.55) | 5.4(4.05-7.24) | 180.83 | 6.81(6.06-7.57) |
| Slovenia | 5.16(2.71-8.54) | 274.22 | 9.08(7.44-10.74) | 10.55(5.99-17.9) | 114.65 | 5.56(5.25-5.86) |
| Solomon Islands | 49.5(5.08-326.12) | 177.65 | 2.59(2.01-3.18) | 123.43(5.89-795.96) | 218.67 | 6.57(6.37-6.78) |
| Somalia | 592.25(349.09-946.92) | 3808.01 | 23.45(16.69-30.61) | 253.07(140.91-425.14) | −28.18 | −5.73(−5.84-−5.62) |
| South Africa | 10333.28(9704.89-10923.35) | 3687.01 | 26.49(21.27-31.94) | 13577.41(12784.2-14340.97) | 54.89 | 1.98(1.88-2.09) |
| South Sudan | 1318.19(513.47-2720.81) | 912.18 | 14.89(12.05-17.8) | 1619.93(602.91-3484.01) | 29.65 | 1.33(1.12-1.55) |
| Spain | 139.95(121.82-161.58) | 22 | −1.17(−1.87-−0.46) | 169.59(142.46-200.72) | 39.2 | 1.46(0.99-1.93) |
| Sri Lanka | 4.49(3.38-6.33) | 82.59 | 2.38(1.06-3.71) | 8.66(7.36-10.07) | 116.62 | 4.87(4.07-5.67) |
| Sudan | 227.82(87.56-464.33) | 763.92 | 13.08(10.68-15.52) | 303.74(94.37-807.24) | 92.2 | 1.9(1.76-2.05) |
| Suriname | 477.73(352.88-626.73) | 339.58 | 7.98(6.9-9.06) | 588.8(499.06-724.56) | 39.89 | 1.79(1.52-2.06) |
| Sweden | 26.18(13.66-42.43) | 45.33 | 0.99(0.42-1.57) | 44.78(23.64-67.33) | 87.04 | 3.99(3.79-4.19) |
| Switzerland | 168.04(113.8-221.62) | 87.8 | 2.62(1.84-3.4) | 185.46(118.31-255.11) | 38.94 | 0.71(0.69-0.74) |
| Syrian Arab Republic | 1.5(1.18-1.96) | 165.17 | 3.57(3.01-4.13) | 2.67(1.91-4.09) | 34 | 4.67(4.14-5.21) |
| Taiwan  (Province of China) | 9.89(6.17-15.99) | 404.61 | 9.13(8.02-10.25) | 18.14(8.63-31.69) | 76.71 | 3.33(2.67-3.98) |
| Tajikistan | 26.84(18.9-38.03) | 382.25 | 9.64(7.9-11.4) | 51.49(38.48-63.24) | 182.49 | 5.12(4.04-6.21) |
| Thailand | 521.09(424.47-678.95) | 331.08 | 5.8(1.98-9.76) | 623.33(536.88-741.37) | 35.29 | 1.28(1.03-1.53) |
| TimorLeste | 391.73(16.25-2366.25) | 333.29 | 4.45(0.85-8.18) | 311.33(17.18-1799.89) | 12.93 | −1.71(−1.97-−1.45) |
| Togo | 2967.32(2247.16-3730.96) | 1171.57 | 15.1(10.66-19.72) | 1697.38(1385.91-2055.05) | −16.69 | −3.69(−3.78-−3.61) |
| Tokelau | 37.16(6.71-166.52) | 331.73 | 12.37(12.21-12.53) | 153.11(10.56-618.15) | 333.58 | 9.77(9.66-9.88) |
| Tonga | 20.76(11.67-35.62) | 152.24 | 5.2(4.82-5.58) | 45.71(19.6-97.25) | 102 | 5.34(5.08-5.6) |
| Trinidad and Tobago | 429.38(344.78-551.83) | 183.62 | 6.27(4.78-7.78) | 592.51(520.91-708.12) | 49.07 | 2.43(2.34-2.53) |
| Tunisia | 10.67(1.59-46.44) | 502.83 | 11.04(10.97-11.1) | 24.48(4.69-91.29) | 140.91 | 5.13(4.75-5.51) |
| Turkey | 2.36(1.73-3.23) | 504.83 | 10.03(9.55-10.51) | 5.81(4.26-8.29) | 168.37 | 5.48(4.65-6.32) |
| Turkmenistan | 79.33(54.46-102.55) | 160.06 | 4.55(3.83-5.27) | 70.02(55.6-91.97) | 2.14 | −1.46(−1.72-−1.19) |
| Tuvalu | 36.1(7.27-158.13) | 413.72 | 12.29(12.07-12.5) | 152.23(10.83-628.81) | 365.26 | 9.98(9.9-10.05) |
| Uganda | 5634.53(5083.81-6280.49) | 3.05 | −2.94(−3.2-−2.69) | 5029.57(4542.77-5551.89) | 24.13 | −0.3(−0.52-−0.07) |
| Ukraine | 259.77(167.99-385.48) | 372.08 | 13.68(12.95-14.4) | 515.98(383.87-664.21) | 97.66 | 4.44(2.78-6.12) |
| United Arab Emirates | 10.1(1.61-41.58) | 766.79 | 10.04(9.73-10.36) | 46.69(3.14-256.26) | 303.01 | 13.53(10.06-17.11) |
| United Kingdom | 97.76(54.72-139.91) | 253.94 | 9.08(8.47-9.69) | 168.23(94.76-243.37) | 90.79 | 3.42(3.22-3.62) |
| United Republic of Tanzania | 5037.15(4588.39-5550.27) | 81.73 | 0.84(−0.53-2.23) | 3607.68(3275.54-3954.77) | 1.18 | −1.85(−2.13-−1.58) |
| United States of America | 352.39(180.29-545.25) | 29.61 | 0.39(0.21-0.57) | 432.45(228.9-648.02) | 42.74 | 1.38(1.25-1.52) |
| United States Virgin Islands | 142.6(110.84-185.16) | 31.33 | 0.95(0.11-1.8) | 215.89(190.25-247.28) | 45.9 | 3.34(2.95-3.73) |
| Uruguay | 195.5(131.26-266.19) | 234.61 | 8.61(7.99-9.23) | 330.96(231.63-443.96) | 71.07 | 3.38(2.96-3.81) |
| Uzbekistan | 31.43(20.35-44.67) | 557.96 | 9.67(8.79-10.57) | 62.79(36.61-87.04) | 142.05 | 4.33(2.9-5.78) |
| Vanuatu | 54.77(7.24-358.42) | 166.62 | 3.05(2.55-3.55) | 130.19(8.98-855.05) | 227.43 | 5.29(4.18-6.41) |
| Venezuela  (Bolivarian Republic of) | 165.32(120.2-214.99) | 545.4 | 10.41(8.96-11.87) | 306.01(238.57-371.35) | 105.25 | 3.81(3.66-3.96) |
| Viet Nam | 131.92(103.08-169.25) | 567.91 | 12.26(11.57-12.96) | 220.36(180-292.82) | 89.51 | 3.7(3.55-3.84) |
| Yemen | 16.28(4.29-57.18) | 132.45 | 2.04(1.85-2.22) | 24.27(2.62-93.23) | 135.14 | 2.68(1.97-3.4) |
| Zambia | 9873.32(9093.87-10699.01) | 153.69 | 3.72(2.01-5.45) | 9223.97(8523.07-9949.47) | 43.63 | −0.1(−0.24-0.03) |
| Zimbabwe | 14323.33(13049.92-15538.57) | 337.14 | 8.36(5.18-11.63) | 10490.93(9862.95-11204.35) | −11.29 | −1.67(−1.9-−1.44) |

EAPC: estimated annual percentage change; ASR, age-standardized rate; CI, confidence interval; UI: uncertainty interval.

**Supplementary table 8**. Age-standardized rates of deaths caused by HIV/AIDS at the national level and for both sexes in 2019, and percentage changes and the EAPCs in the number of deaths from 1990 to 2019.

|  | **1990-2004** | | | **2005-2019** | | |
| --- | --- | --- | --- | --- | --- | --- |
| **Characteristics** | ASR/100,000  (95% UI) | Change in  number (%) | EAPC  (95%CI) | ASR/100,000  (95% UI) | Change in  number (%) | EAPC  (95%CI) |
| Afghanistan | 0.71(0.06-2.73) | 264.51 | 4.59(4.02-5.16) | 0.96(0.02-4.9) | 140.58 | 1.65(1.29-2.01) |
| Albania | 0.06(0.06-0.06) | 52.22 | 1.8(0.95-2.66) | 0.06(0.05-0.07) | −13.91 | −1.18(−1.57-−0.79) |
| Algeria | 0.65(0.09-4.02) | 340.38 | 8.12(7.59-8.66) | 0.62(0.04-4.59) | 21.49 | −1.33(−2.88-0.25) |
| American Samoa | 0.97(0.62-1.52) | 528.4 | 10.1(7.86-12.39) | 0.93(0.17-2.51) | −11.97 | −0.01(−0.68-0.68) |
| Andorra | 3.16(0.27-12.68) | 135.97 | 2.04(1.01-3.08) | 2.59(0.18-11.61) | −5.13 | −1.51(−1.86-−1.16) |
| Angola | 62.79(41.86-90.6) | 3473.35 | 25.33(22.29-28.45) | 77.73(55.01-110.16) | 78.78 | 0.99(0.51-1.47) |
| Antigua and Barbuda | 12.03(11.79-12.25) | 51.51 | 1.49(−0.28-3.29) | 7.09(6.97-7.23) | −19.52 | −1.72(−2.73-−0.69) |
| Argentina | 4.71(4.62-4.79) | 212.44 | 6.66(3.48-9.95) | 3.68(3.6-3.76) | 9.68 | −1.14(−1.44-−0.84) |
| Armenia | 0.07(0.07-0.07) | 107.6 | 1.31(−0.98-3.65) | 0.56(0.53-0.59) | 382.74 | 10.61(6.24-15.15) |
| Australia | 0.62(0.61-0.64) | −61.64 | −12.39(−15.31-−9.36) | 0.24(0.23-0.25) | −39.38 | −5.95(−7.37-−4.52) |
| Austria | 0.58(0.56-0.61) | −18.64 | −7.94(−11.69-−4.04) | 0.34(0.32-0.35) | −45.81 | −3.38(−4.78-−1.96) |
| Azerbaijan | 0.66(0.56-0.74) | 317.22 | 8.49(7.48-9.51) | 0.31(0.25-0.36) | −40.73 | −6.24(−8.23-−4.21) |
| Bahamas | 57.45(56.43-58.47) | 220.87 | 4.6(1.48-7.81) | 26.56(26.05-27.06) | −31.37 | −3.83(−4.71-−2.94) |
| Bahrain | 1.08(1.01-1.15) | 140.53 | 2.43(0.17-4.74) | 0.38(0.3-0.51) | −23.64 | −6.6(−7.43-−5.77) |
| Bangladesh | 0.06(0-0.26) | 30607.68 | 28.42(22.23-34.92) | 0.23(0-1.46) | 267.57 | 7.9(5.52-10.34) |
| Barbados | 12.84(12.6-13.09) | 3.28 | 0.31(−2.15-2.84) | 7.1(6.97-7.23) | −35.35 | −3.7(−4.29-−3.11) |
| Belarus | 2.17(2.08-2.28) | 149.96 | 6.28(4.91-7.67) | 2.69(2.54-2.86) | 12.12 | 0.15(−1.43-1.75) |
| Belgium | 0.81(0.78-0.84) | −23.76 | −6.12(−9.43-−2.68) | 0.41(0.39-0.42) | −31.29 | −3.09(−4.06-−2.12) |
| Belize | 35.55(34.86-36.19) | 183.57 | 6(4.28-7.75) | 20.18(19.79-20.61) | 16.32 | −1.53(−2.5-−0.55) |
| Benin | 83.41(63.22-105.37) | 9236.65 | 32.16(22.8-42.23) | 22.78(17.15-29.76) | −57.76 | −8.6(−11.02-−6.1) |
| Bermuda | 13.69(13.43-13.95) | −24.66 | −1.7(−3.52-0.16) | 7.53(7.38-7.69) | −29.15 | −2.02(−3-−1.04) |
| Bhutan | 3.56(0.23-14.37) | 321.54 | 7.99(6.16-9.86) | 4(0.07-25.68) | 41.55 | 1.06(0.51-1.61) |
| Bolivia  (Plurinational State of) | 8.1(1.59-27.12) | 208.71 | 4.56(3-6.14) | 6.1(0.35-38.28) | 13.15 | −1.36(−2.1-−0.62) |
| Bosnia and Herzegovina | 0.08(0.08-0.09) | 9.3 | 0.55(−0.02-1.12) | 0.04(0.03-0.07) | −49.67 | −1.45(−3.23-0.37) |
| Botswana | 991.93(824.15-1207.17) | 1123.45 | 17.29(12.65-22.13) | 258.09(216.64-320.45) | −54.79 | −7.58(−8.63-−6.52) |
| Brazil | 7.91(7.84-8) | 82.86 | 0.52(−1.65-2.74) | 6.39(6.28-6.5) | 4.78 | −1.29(−1.41-−1.16) |
| Brunei Darussalam | 0.98(0.9-1.09) | 328.92 | 5.85(4.7-7.01) | 1.14(1-1.3) | 52.09 | −0.15(−1.17-0.88) |
| Bulgaria | 0.71(0.68-0.74) | 116.23 | 7.63(4.12-11.26) | 0.57(0.54-0.6) | −51.99 | −4.54(−5.42-−3.65) |
| Burkina Faso | 129.78(110.84-153.87) | 1.42 | −2.72(−4.46-−0.94) | 18.11(14.46-22.82) | −74.06 | −11.67(−12.21-−11.12) |
| Burundi | 278.48(223.76-335.16) | 123.53 | 4.34(1.3-7.48) | 23.9(19.11-31.41) | −84.04 | −15.28(−16.08-−14.47) |
| Cabo Verde | 42.2(23.97-68.35) | 465.1 | 9.83(7.15-12.57) | 10.11(5.17-22.36) | −63 | −8.66(−9.08-−8.24) |
| Cambodia | 70.62(51.29-95.62) | 92231.16 | 56.37(38.56-76.46) | 9.07(5.99-12.93) | −80.57 | −13.21(−14.26-−12.14) |
| Cameroon | 263.02(195.4-336.38) | 2893.38 | 23.62(19.35-28.05) | 109.67(96.96-127.76) | −35.15 | −5.07(−6.14-−3.99) |
| Canada | 1.17(1.13-1.2) | −43 | −8.91(−12.1-−5.61) | 0.58(0.55-0.61) | −49.23 | −5.64(−7.04-−4.21) |
| Central African Republic | 417.52(330.62-510.24) | 522.46 | 10.12(5.74-14.67) | 141.84(115.86-185.49) | −54.67 | −6.66(−7.33-−6) |
| Chad | 103.38(69.9-150.09) | 839.98 | 14.16(11.05-17.36) | 46.21(32.63-64.76) | −30.94 | −4.34(−6.07-−2.59) |
| Chile | 2.5(2.45-2.55) | 305.32 | 9.71(5.98-13.57) | 2.35(2.29-2.4) | 18.36 | 0.44(−0.36-1.24) |
| China | 0.9(0.85-0.98) | 397.65 | 9.04(7.87-10.22) | 1.74(1.43-2.05) | 110.37 | 5.01(3.75-6.3) |
| Colombia | 6.56(6.39-6.71) | 452.34 | 10.44(8.97-11.94) | 4.78(4.56-5.01) | −8.01 | −2.02(−2.29-−1.76) |
| Comoros | 0.17(0.01-0.97) | 1648.57 | 15.96(14.36-17.57) | 0.28(0-2.09) | 91.29 | 0.43(−0.51-1.38) |
| Congo | 280.38(224.53-348.47) | 145.01 | 3.38(1.53-5.27) | 91.42(76.9-111.24) | −45.92 | −6.9(−7.42-−6.37) |
| Cook Islands | 2.23(0.2-11.74) | 759.53 | 14.95(14.18-15.72) | 8.88(0.13-42.96) | 258.2 | 9.6(9.51-9.7) |
| Costa Rica | 3.69(3.57-3.82) | 152.68 | 1.83(−0.86-4.59) | 2.82(2.69-2.95) | −3.02 | −1.35(−2.44-−0.24) |
| Croatia | 0.12(0.11-0.12) | −6.26 | −1.42(−5.68-3.02) | 0.15(0.14-0.15) | 58.78 | 0.96(−3.61-5.75) |
| Cuba | 0.89(0.87-0.91) | 92.56 | 1.76(−0.77-4.36) | 2.57(2.51-2.64) | 157.99 | 8.96(6.54-11.44) |
| Cyprus | 0.23(0.22-0.24) | 346.39 | 8.16(7.06-9.27) | 0.27(0.25-0.3) | 61.18 | 0.76(0.17-1.35) |
| Czechia | 0.04(0.04-0.05) | 4.1 | −1.2(−5.19-2.97) | 0.15(0.14-0.15) | 88.51 | 5.79(3.19-8.46) |
| Cote d'Ivoire | 368.36(264.08-475.49) | 429.45 | 9.09(5.56-12.73) | 67.64(56.7-80.01) | −72.17 | −10.15(−11.2-−9.1) |
| Democratic People's Republic of Korea | 1.27(0.04-8.44) | 976.8 | 16.4(14.35-18.5) | 2.56(0.06-13.58) | 110.1 | 5.71(4.26-7.17) |
| Democratic Republic of the Congo | 108.46(83.76-137.09) | 149.88 | 3.93(2.1-5.78) | 15.46(11.76-20.97) | −76.66 | −12.05(−13.58-−10.5) |
| Denmark | 0.71(0.68-0.75) | 1965.1 | 20.03(1.32-42.19) | 0.34(0.33-0.36) | −38.29 | −3.42(−4.85-−1.97) |
| Djibouti | 135.54(96.67-182.35) | 97883.94 | 57.26(43.36-72.52) | 81.48(58.4-116.4) | −3.95 | −4.05(−4.65-−3.44) |
| Dominica | 11(10.76-11.25) | 29.89 | 2.73(1.09-4.41) | 6.89(6.74-7.03) | −19.5 | −0.85(−1.91-0.22) |
| Dominican Republic | 43.67(32.62-56.41) | 934.63 | 13.75(8.93-18.79) | 12.65(9.17-18.46) | −60.28 | −7.42(−7.97-−6.86) |
| Ecuador | 5.69(5.51-5.89) | 1006.19 | 14.79(12.46-17.16) | 6.91(6.46-7.39) | 40.04 | 0.34(−0.21-0.89) |
| Egypt | 0.17(0.16-0.18) | 104.56 | 2.42(1.2-3.66) | 0.06(0.05-0.08) | −51.82 | −6.95(−8.38-−5.49) |
| El Salvador | 15.07(11.88-17.32) | 773.01 | 16.38(15.24-17.53) | 11.36(5.97-16.39) | −8.1 | −1.84(−2.41-−1.27) |
| Equatorial Guinea | 181.98(121.67-267.53) | 4380.33 | 26.97(23.76-30.27) | 204.35(130.73-323.02) | 83.51 | 0.43(−0.3-1.17) |
| Eritrea | 107.16(74.49-145.53) | 703.28 | 12.37(8.61-16.27) | 27.04(19.76-36.03) | −62.14 | −9.04(−9.5-−8.58) |
| Estonia | 1.41(1.34-1.48) | 3174.84 | 27.07(20.37-34.15) | 2.56(2.4-2.72) | 5.05 | −0.31(−2.13-1.56) |
| Eswatini | 1218.02(843.71-1623.24) | 40302.08 | 48.51(37.32-60.62) | 353.13(307.81-417.71) | −66.91 | −9.11(−9.56-−8.66) |
| Ethiopia | 182.1(132.7-233.23) | 546.03 | 12.26(8.22-16.45) | 33.56(28.68-39.58) | −69.58 | −10.7(−11.6-−9.8) |
| Fiji | 1.71(1.5-1.94) | 511.12 | 11.91(9.88-13.97) | 0.95(0.64-1.48) | −35.69 | −3.56(−4.14-−2.98) |
| Finland | 0.18(0.17-0.19) | −22.89 | −6.82(−10.74-−2.72) | 0.08(0.08-0.09) | −41.48 | −6.37(−9.12-−3.53) |
| France | 1.58(1.54-1.61) | −62.83 | −12.57(−16.07-−8.92) | 0.57(0.55-0.6) | −54.01 | −6.11(−7.7-−4.49) |
| Gabon | 198.71(132.64-293.53) | 1380.19 | 18.74(15-22.59) | 72.44(52.05-103.04) | −45.86 | −6.58(−7.33-−5.83) |
| Gambia | 91.32(58.87-135.15) | 4219.88 | 27.88(24.05-31.84) | 67.57(45.48-97.17) | −1.39 | −2.6(−3.19-−2) |
| Georgia | 0.04(0.04-0.05) | 7.52 | 2.4(0.73-4.11) | 0.58(0.56-0.61) | 1577.04 | 28.87(18.33-40.35) |
| Germany | 0.6(0.58-0.61) | −63.63 | −11.69(−14.47-−8.82) | 0.39(0.38-0.41) | −29.24 | −2.66(−3.01-−2.31) |
| Ghana | 134.65(106.87-166.25) | 773.57 | 13.82(11.11-16.6) | 55.64(46.93-67.75) | −39.21 | −6.13(−6.45-−5.81) |
| Greece | 0.13(0.12-0.13) | −57.74 | −9.16(−12.53-−5.66) | 0.2(0.19-0.21) | 87.35 | 8.76(3.61-14.17) |
| Greenland | 4(3.64-4.41) | 15.48 | −2.17(−4.27-−0.03) | 2.52(2.02-3) | −37.33 | −3.78(−4.66-−2.89) |
| Grenada | 8.08(7.93-8.25) | 26.53 | 0.22(−1.07-1.52) | 4.83(4.73-4.93) | −25.14 | −1.52(−2.51-−0.52) |
| Guam | 3.14(2.22-4.46) | 308.5 | 6.78(4.43-9.19) | 4.07(1.71-8.68) | 30.79 | 1.7(1.37-2.02) |
| Guatemala | 7.43(7.25-7.6) | 193.51 | 3.16(2.25-4.08) | 4.16(3.99-4.36) | −22.94 | −5.57(−6.5-−4.63) |
| Guinea | 70.61(50.28-97.54) | 1733.75 | 21.13(18.17-24.16) | 45.81(32.64-64.22) | −15.8 | −2.72(−3.46-−1.99) |
| Guinea-Bissau | 143.2(94.44-212.2) | 2537.79 | 24.58(20.93-28.34) | 73.58(41.72-121.54) | −35.29 | −5.07(−5.33-−4.81) |
| Guyana | 45.87(45.09-46.67) | 286.4 | 12.77(9.78-15.85) | 23.51(23.08-23.93) | −42.59 | −3.58(−4.34-−2.81) |
| Haiti | 187.14(140.7-240.22) | 382.23 | 8.39(5.13-11.75) | 54.64(44.05-69.6) | −56.6 | −7.85(−8.35-−7.35) |
| Honduras | 2.75(2.33-3.38) | 92.59 | 3.58(1.2-6.01) | 1.01(0.77-1.31) | −4.79 | −2.69(−3.42-−1.94) |
| Hungary | 0.86(0.82-0.89) | −32.8 | −5.31(−7.41-−3.16) | 0.28(0.27-0.3) | −60.33 | −7.31(−9.12-−5.47) |
| Iceland | 0.44(0.42-0.46) | −30.38 | −6.95(−9.75-−4.06) | 0.24(0.23-0.25) | −32.01 | −3.54(−4.15-−2.93) |
| India | 14.86(11.66-18.34) | 13833.7 | 42.94(35.58-50.7) | 3.26(2.89-3.72) | −71.94 | −11.84(−12.74-−10.94) |
| Indonesia | 0.86(0.76-0.95) | 16859.51 | 31.24(21.46-41.8) | 2.11(1.67-2.81) | 137.89 | 6.07(5.1-7.06) |
| Iran  (Islamic Republic of) | 0.48(0.42-0.54) | 712.8 | 12.38(11.76-13) | 1.26(0.91-1.82) | 229.25 | 6.41(6.07-6.75) |
| Iraq | 0.17(0.09-0.4) | 649.26 | 11.1(10.71-11.5) | 0.19(0.04-0.76) | 56.55 | 0.89(0.07-1.72) |
| Ireland | 0.28(0.27-0.3) | 12.02 | −8.09(−13.98-−1.8) | 0.16(0.16-0.17) | 4.59 | −1.76(−5.77-2.42) |
| Israel | 0.44(0.42-0.46) | −21.76 | −6.29(−8.36-−4.18) | 0.38(0.37-0.4) | 11.93 | −1.56(−3.18-0.08) |
| Italy | 1.63(1.6-1.65) | −37.85 | −10.43(−14.94-−5.67) | 0.77(0.75-0.78) | −43.79 | −4.85(−5.49-−4.2) |
| Jamaica | 24.6(24.22-24.99) | 248.04 | 8.4(6.75-10.08) | 14.08(13.84-14.34) | 15.52 | −1.5(−2.85-−0.12) |
| Japan | 0.11(0.11-0.11) | 174.68 | 6.83(3.96-9.77) | 0.09(0.09-0.09) | −20.66 | −2(−2.51-−1.5) |
| Jordan | 0.27(0.23-0.3) | 494 | 11.37(10.76-11.97) | 0.25(0.18-0.34) | 121.39 | 3.09(1.47-4.75) |
| Kazakhstan | 1.53(1.47-1.58) | 206.89 | 7.12(2.81-11.61) | 1.28(1.21-1.35) | −12.28 | −2.41(−2.98-−1.84) |
| Kenya | 539.45(438.98-634.17) | 506.69 | 10.66(7.09-14.35) | 133.48(118.75-151.17) | −58.97 | −9.07(−9.54-−8.59) |
| Kiribati | 1.73(1.58-1.88) | 146.73 | 4.14(2.46-5.85) | 1.29(0.98-1.72) | 2.49 | −1.82(−2.28-−1.36) |
| Kuwait | 0.1(0.09-0.11) | 6.64 | −3.9(−8.47-0.91) | 0.06(0.05-0.06) | −48.91 | −8.24(−11.43-−4.94) |
| Kyrgyzstan | 2.33(2.24-2.42) | 305.88 | 10.11(7.35-12.95) | 2.65(2.52-2.77) | 24.84 | 1.44(−0.11-3) |
| Lao People's Democratic Republic | 3.63(0.05-22.55) | 647951.89 | 72.88(57.16-90.19) | 5.65(0.04-41.22) | 72.58 | 1.78(−0.28-3.89) |
| Latvia | 3.16(3.03-3.3) | 93.45 | 4.98(2.52-7.5) | 5.55(5.28-5.83) | 40.2 | 3.63(2.07-5.21) |
| Lebanon | 1.72(0.05-8.27) | 33.31 | 0.6(−0.59-1.79) | 1.15(0.03-5.34) | −0.95 | −2.53(−2.85-−2.21) |
| Lesotho | 1137.68(830.79-1482.74) | 3433 | 28.12(23.34-33.1) | 581.38(500.13-710.7) | −40.58 | −4.02(−5.05-−2.97) |
| Liberia | 99.27(65.99-144.34) | 1807.71 | 19.76(16.29-23.32) | 43.18(33.2-56.39) | −32.54 | −5.02(−5.66-−4.38) |
| Libya | 0.86(0.04-3.75) | 461.53 | 10.44(10.19-10.7) | 1.15(0.02-6.41) | 76.18 | 1.35(0.9-1.8) |
| Lithuania | 2.12(2.02-2.22) | 201.69 | 8.18(4.98-11.48) | 1.88(1.79-1.96) | −1.26 | 0.68(−0.61-1.99) |
| Luxembourg | 0.65(0.62-0.69) | −34.52 | −8.17(−11.22-−5.01) | 0.34(0.32-0.35) | −23.91 | −3.96(−4.66-−3.25) |
| Madagascar | 7.85(5.02-12.86) | 77771.24 | 61.91(52.66-71.71) | 12.03(7.93-17.36) | 101.73 | 2.19(1.54-2.85) |
| Malawi | 804.28(640.93-981.77) | 411.4 | 10.7(7.88-13.59) | 111.2(97.02-131.68) | −79.46 | −13.51(−14.15-−12.87) |
| Malaysia | 7.9(7.29-8.45) | 4589.27 | 23.58(16.08-31.56) | 4.9(3.13-6.82) | −23.81 | −1.75(−3.65-0.18) |
| Maldives | 0.21(0.19-0.23) | 527.01 | 11.03(10.72-11.35) | 0.17(0.15-0.18) | 56.67 | −0.61(−1.62-0.41) |
| Mali | 72.39(53.15-95.53) | 1137.23 | 17.28(13.73-20.95) | 31.45(23.48-40.62) | −31.9 | −4.17(−5.37-−2.95) |
| Malta | 0.46(0.44-0.48) | −19.74 | −5.98(−9.15-−2.7) | 0.28(0.27-0.3) | −28.89 | −3(−3.32-−2.68) |
| Marshall Islands | 3.72(0.08-26.22) | 305.35 | 7.41(4.96-9.93) | 7.31(0.04-59.33) | 131.72 | 4.96(4.55-5.36) |
| Mauritania | 2.36(0.05-16.28) | 4.79 | −3.43(−4.93-−1.9) | 0.69(0.02-5.23) | −49.17 | −5.95(−7.53-−4.35) |
| Mauritius | 1.32(1.24-1.41) | 74.65 | 10.8(1.46-20.99) | 6.05(5.64-6.5) | 290.22 | 9.49(6.1-12.99) |
| Mexico | 5.31(5.27-5.36) | 161.37 | 3.01(1.26-4.79) | 3.85(3.8-3.89) | −6.53 | −2.61(−3-−2.22) |
| Micronesia  (Federated States of) | 8.13(1.61-43.18) | 20.93 | 2.74(1.89-3.59) | 49.69(1.18-257.1) | 420.12 | 12.75(12.1-13.39) |
| Monaco | 1.62(0.51-4.15) | 82.23 | 0.99(−0.14-2.12) | 1.58(0.33-5.12) | 9.66 | −0.29(−0.71-0.13) |
| Mongolia | 0.26(0.17-0.35) | 49806.17 | 56.42(43.72-70.25) | 0.46(0.05-1.18) | 183.46 | 5.03(4.55-5.51) |
| Montenegro | 0.43(0.34-0.5) | 234.95 | 9.39(7.64-11.17) | 0.23(0.2-0.28) | −50.54 | −3.1(−3.9-−2.3) |
| Morocco | 2.53(0.13-11.31) | 655.56 | 13.75(12.71-14.79) | 1.36(0.03-8.25) | −36.71 | −4.31(−5.81-−2.8) |
| Mozambique | 430.14(324.7-563.33) | 3012.07 | 24.99(21.54-28.53) | 292.95(244.05-377.11) | −11.6 | −2.88(−3.58-−2.17) |
| Myanmar | 30.45(23.98-38.68) | 32489.08 | 41.85(30.1-54.66) | 8.38(6.66-10.67) | −68.6 | −9.56(−11.59-−7.47) |
| Namibia | 695.82(545.4-874.4) | 3913.44 | 28.03(23.09-33.16) | 187.83(162.72-221.6) | −62.15 | −7.06(−8.71-−5.38) |
| Nauru | 2.27(0.2-11.93) | 748.05 | 15.44(14.69-16.21) | 8.82(0.13-41.96) | 228.97 | 9.41(9.29-9.53) |
| Nepal | 6.01(0.35-28.82) | 2526239.94 | 95.37(76.31-116.49) | 5.13(0.09-31.22) | −11.99 | −3.17(−4.87-−1.44) |
| Netherlands | 0.56(0.54-0.58) | −63.62 | −11.43(−14.36-−8.4) | 0.24(0.23-0.25) | −50.52 | −3.84(−5.28-−2.38) |
| New Zealand | 0.33(0.33-0.34) | −71.19 | −12.99(−14.98-−10.96) | 0.18(0.18-0.19) | −40 | −3.44(−6.13-−0.68) |
| Nicaragua | 3.25(2.72-3.67) | 693.21 | 13.77(12.83-14.71) | 8.82(5.15-12.88) | 220.31 | 8.09(6.57-9.64) |
| Niger | 42.39(31.25-55.98) | 1144.82 | 16.13(12.42-19.96) | 10.83(7.65-14.47) | −62.1 | −8.72(−9.5-−7.93) |
| Nigeria | 104.53(77.07-143.28) | 1321.09 | 17.45(13.68-21.35) | 54.11(44.15-66.62) | −24.54 | −4.57(−5.37-−3.77) |
| Niue | 2.28(0.2-11.87) | 530.99 | 15.32(14.66-15.99) | 8.58(0.13-41.13) | 270.49 | 9.14(9.03-9.24) |
| North Macedonia | 0.1(0.09-0.12) | 250.97 | 9.01(8.29-9.72) | 0.1(0.08-0.12) | −21.16 | −1.82(−2.93-−0.69) |
| Northern Mariana Islands | 1.26(0.78-2) | 831.79 | 12.73(10.63-14.87) | 1.32(0.21-3.25) | −39.29 | −0.21(−0.82-0.4) |
| Norway | 0.49(0.48-0.5) | −21.33 | −8.47(−12.23-−4.54) | 0.23(0.22-0.23) | −46.47 | −4.18(−6.24-−2.08) |
| Oman | 1.28(0.84-1.79) | 533.46 | 11.82(9.44-14.26) | 2.24(1.22-3.58) | 224.61 | 1.16(−0.79-3.15) |
| Pakistan | 0.48(0.03-2.21) | 104.79 | 1.28(−0.02-2.59) | 2.02(0.02-12.39) | 520.2 | 11.74(11.02-12.47) |
| Palau | 2.08(0.18-11.1) | 1012.66 | 14.64(13.8-15.49) | 8.22(0.12-38.9) | 257.03 | 9.54(9.37-9.71) |
| Palestine | 0.23(0.22-0.24) | 867.34 | 11.58(10.81-12.36) | 0.29(0.25-0.34) | 58.67 | 0.74(0.38-1.1) |
| Panama | 15.84(15.44-16.25) | 205.6 | 6.29(4.45-8.17) | 12.81(12.22-13.42) | 11.23 | −1.24(−1.8-−0.69) |
| Papua New Guinea | 50.65(18.22-110.93) | 149970.74 | 67.62(56.35-79.71) | 45.23(15.3-126.67) | 28.84 | −1.41(−1.69-−1.13) |
| Paraguay | 6.01(4.51-7.07) | 775.68 | 14.16(13.65-14.68) | 8.47(5.06-12.23) | 57.33 | 3.16(1.37-4.98) |
| Peru | 8.69(7.38-9.57) | 351.42 | 9.84(9.25-10.44) | 7.03(4.08-11.48) | 0.58 | −0.24(−1.72-1.26) |
| Philippines | 4.92(4.86-5) | 431.41 | 9.86(9.84-9.88) | 4.62(4.54-4.7) | 13.29 | −2.05(−2.66-−1.44) |
| Poland | 0.38(0.37-0.39) | 662.28 | 23.01(17.48-28.8) | 0.3(0.3-0.31) | −12.81 | −1.29(−2.55-−0.02) |
| Portugal | 8.1(7.86-8.35) | 373.29 | 8.77(3.85-13.91) | 3.66(3.48-3.86) | −52.76 | −6.11(−7.46-−4.73) |
| Puerto Rico | 13.71(13.47-13.94) | −38.52 | −7.36(−10.1-−4.54) | 5.45(5.35-5.57) | −60.03 | −6.73(−8.12-−5.33) |
| Qatar | 0.24(0.19-0.28) | −5.71 | −5.76(−9.53-−1.83) | 0.14(0.12-0.16) | 98.01 | −3.98(−4.79-−3.17) |
| Republic of Korea | 0.15(0.14-0.15) | 56.24 | 0(−3.71-3.85) | 0.18(0.17-0.19) | 49.92 | 0.02(−1.41-1.47) |
| Republic of Moldova | 4.82(4.64-5.02) | 299.63 | 9.12(7.22-11.05) | 3.66(3.43-3.88) | −26.03 | −3.23(−4-−2.46) |
| Romania | 1.13(1.08-1.18) | 8.56 | 3.57(−1.99-9.45) | 0.77(0.73-0.81) | −21.94 | 1.36(−0.61-3.36) |
| Russian Federation | 6.44(6.4-6.48) | 201.77 | 7.92(6.71-9.14) | 11.39(11.27-11.5) | 68.17 | 4.62(3.56-5.68) |
| Rwanda | 218.46(162.09-280.42) | 601.85 | 13.5(9.21-17.96) | 32.72(27.71-38.85) | −74.11 | −11(−11.94-−10.05) |
| Saint Kitts and Nevis | 29(7.67-56.67) | 917.63 | 14.41(12.15-16.71) | 44.59(7.55-116.05) | 100.25 | 3.07(2.94-3.2) |
| Saint Lucia | 6.53(6.4-6.66) | 30.61 | 1.07(−0.39-2.56) | 3.53(3.46-3.59) | −21.54 | −2.64(−3.46-−1.81) |
| Saint Vincent and the Grenadines | 27.46(26.91-28.05) | 96.89 | 6.6(4.28-8.98) | 16.17(15.83-16.48) | −22.08 | −1.66(−2.82-−0.48) |
| Samoa | 3.53(0.08-24.78) | 305.28 | 7.63(5.28-10.04) | 7.45(0.03-59.35) | 145.17 | 5.57(5.27-5.88) |
| San Marino | 1.69(0.54-4.36) | 97.74 | 1.5(0.18-2.84) | 1.54(0.33-4.91) | 11.72 | −0.75(−1.14-−0.36) |
| Sao Tome and Principe | 1.08(0.84-1.43) | 318.81 | 7.54(5.35-9.77) | 0.16(0.09-0.27) | −62.2 | −6.76(−9.49-−3.94) |
| Saudi Arabia | 1.75(1.21-3.38) | 425.02 | 9.07(8.78-9.36) | 1.68(0.65-4.84) | 74.4 | −0.71(−0.99-−0.43) |
| Senegal | 43.47(31.56-57.71) | 1224.86 | 17.34(14.02-20.75) | 14.22(10.87-18.77) | −56.36 | −5.75(−7.52-−3.95) |
| Serbia | 0.61(0.49-0.93) | 96.72 | 4.42(3.53-5.32) | 0.51(0.32-1.06) | −2.14 | 1.27(−1.78-4.42) |
| Seychelles | 5.85(5.53-6.12) | 1754.7 | 17.83(12.6-23.31) | 3.33(2.55-4.27) | −21.18 | −1.84(−2.84-−0.83) |
| Sierra Leone | 65.09(46.18-86.66) | 2007.61 | 21.49(18.31-24.75) | 44.5(35.58-58.11) | −4.23 | −2.84(−3.37-−2.3) |
| Singapore | 0.82(0.77-0.86) | 405.93 | 10.41(5.38-15.69) | 0.45(0.42-0.48) | −5.42 | −4.15(−6.51-−1.73) |
| Slovakia | 0.1(0.08-0.12) | 499.86 | 11.02(7.61-14.54) | 0.07(0.06-0.08) | −8.57 | 0.35(−1.47-2.21) |
| Slovenia | 0.1(0.09-0.11) | −43.65 | −0.77(−6.21-4.99) | 0.09(0.09-0.1) | −29.96 | 1.48(−4.43-7.75) |
| Solomon Islands | 4.02(0.09-27.71) | 360.78 | 7.56(5.26-9.9) | 7.25(0.04-59.74) | 127.47 | 4.24(3.75-4.72) |
| Somalia | 46.33(31.81-68.18) | 15421.91 | 36.97(28.4-46.1) | 22.01(16.35-29.96) | −24 | −5.43(−5.84-−5.03) |
| South Africa | 639.03(479.73-827.1) | 9956.86 | 36.88(31.13-42.89) | 250.91(219.7-302.51) | −52.31 | −7.28(−8.01-−6.55) |
| South Sudan | 84.75(40.94-164.29) | 1872.97 | 21.47(18.18-24.86) | 65(27.83-132.83) | −19.64 | −2.25(−2.84-−1.66) |
| Spain | 3.15(3.06-3.24) | −21.85 | −8.18(−12.29-−3.88) | 1.09(1.03-1.15) | −55.76 | −7.23(−8.43-−6.02) |
| Sri Lanka | 0.41(0.38-0.44) | 185.62 | 5.93(4.4-7.5) | 0.17(0.12-0.26) | −54.22 | −5.08(−6.15-−4.01) |
| Sudan | 14.92(7.83-28.59) | 1602.07 | 19.04(15.91-22.26) | 16.08(8.3-31.48) | 45.56 | 0.4(0.09-0.72) |
| Suriname | 33.83(33.26-34.4) | 179.88 | 8.16(6.39-9.97) | 16.55(16.26-16.85) | −36.4 | −3.75(−4.52-−2.98) |
| Sweden | 0.27(0.26-0.28) | −58.41 | −10.11(−13.88-−6.17) | 0.17(0.17-0.18) | −34.58 | −3.43(−5.27-−1.55) |
| Switzerland | 0.94(0.9-0.98) | 2725.8 | 33.07(11.34-59.05) | 0.34(0.32-0.35) | −48.4 | −6.24(−7.62-−4.85) |
| Syrian Arab Republic | 0.14(0.12-0.15) | 191.85 | 5.42(4.89-5.95) | 0.11(0.08-0.14) | −40.51 | −1.17(−1.98-−0.36) |
| Taiwan  (Province of China) | 0.32(0.3-0.34) | 918.26 | 13.29(7.16-19.76) | 0.57(0.52-0.62) | 45.46 | 2.32(0.44-4.24) |
| Tajikistan | 2.17(1.93-2.38) | 1389.75 | 17.63(14.74-20.59) | 0.67(0.51-0.98) | −50.04 | −9.04(−10.26-−7.79) |
| Thailand | 43.92(40.21-47.52) | 3126.35 | 21.37(14.09-29.13) | 19.33(14.61-29.79) | −41.25 | −2.13(−3.98-−0.24) |
| Timor-Leste | 31.62(0.85-182.89) | 1246.88 | 16.41(9.26-24.02) | 21.15(0.2-131) | −4.29 | −2.06(−2.51-−1.62) |
| Togo | 236.87(172.97-306.19) | 2698.93 | 23.83(18.97-28.9) | 54.75(41.79-71.44) | −65.27 | −9.64(−10.42-−8.86) |
| Tokelau | 2.37(0.2-12.35) | 573.97 | 15.45(14.74-16.16) | 8.92(0.14-43.21) | 283.42 | 9.18(9.04-9.31) |
| Tonga | 1.48(0.96-2.25) | 452.32 | 10.67(8.38-13) | 2.04(0.38-5.54) | 36.11 | 2.63(2.2-3.06) |
| Trinidad and Tobago | 23(22.6-23.42) | 224.49 | 9.12(5.86-12.47) | 12.9(12.64-13.14) | −35.36 | −3.1(−3.85-−2.34) |
| Tunisia | 0.63(0.06-3.7) | 561.1 | 12.05(11.46-12.65) | 1.16(0.06-6.33) | 110.31 | 4.16(3.1-5.23) |
| Turkey | 0.15(0.13-0.17) | 1317.61 | 17.12(15.33-18.94) | 0.26(0.21-0.31) | 87.07 | 6.28(4.12-8.49) |
| Turkmenistan | 3.37(3.23-3.5) | 154.2 | 4.43(1.41-7.55) | 1.96(1.84-2.09) | −35.17 | −4.17(−4.83-−3.5) |
| Tuvalu | 2.22(0.2-11.55) | 701.62 | 15.33(14.7-15.95) | 8.66(0.13-42.03) | 320.02 | 9.4(9.26-9.54) |
| Uganda | 482.14(425.16-550.06) | 16.02 | −1.93(−2.58-−1.26) | 72.63(60.48-90.53) | −75.66 | −12.73(−13.51-−11.94) |
| Ukraine | 10.77(10.48-11.08) | 288.31 | 11.14(9.35-12.96) | 12.63(12.08-13.2) | −25.05 | −3.25(−4.5-−1.98) |
| United Arab Emirates | 0.72(0.04-3.18) | 925.52 | 11.12(10.75-11.48) | 4.32(0.03-29.87) | 329.05 | 16.6(14.66-18.57) |
| United Kingdom | 0.42(0.42-0.43) | −37.08 | −7.78(−11.03-−4.41) | 0.3(0.3-0.31) | −26.01 | −3.76(−4.73-−2.79) |
| United Republic of Tanzania | 445.3(348.29-543) | 254.12 | 6.19(3.85-8.58) | 65.29(55.07-78.53) | −77.99 | −12.98(−13.59-−12.37) |
| United States of America | 4.16(4.14-4.19) | −48.12 | −9.22(−11.97-−6.39) | 1.77(1.75-1.78) | −49.04 | −5.95(−7.08-−4.81) |
| United States Virgin Islands | 10.42(10.22-10.61) | 74.04 | 2.53(0.92-4.17) | 6.46(6.33-6.58) | −32.57 | −1.43(−2.41-−0.44) |
| Uruguay | 4.59(4.5-4.69) | 250.69 | 9.1(7.56-10.66) | 4.51(4.41-4.62) | −1.48 | −0.56(−1.38-0.27) |
| Uzbekistan | 1.49(1.44-1.54) | 187.17 | 7.32(5.69-8.98) | 1.67(1.58-1.76) | 48.83 | 0.15(−0.62-0.93) |
| Vanuatu | 3.93(0.08-24.95) | 328.67 | 7.33(5.11-9.59) | 7.77(0.04-62.5) | 161.04 | 4.8(4.43-5.16) |
| Venezuela  (Bolivarian Republic of) | 6.32(6.18-6.46) | 135.51 | 3.45(2.12-4.81) | 6.08(5.8-6.39) | 16.7 | −0.17(−0.99-0.66) |
| Viet Nam | 7.43(5.9-8.54) | 2226.78 | 21.1(18.28-23.99) | 5.6(4.31-7.5) | −13.31 | −2.57(−3.14-−1.99) |
| Yemen | 1.31(0.12-5.1) | 196.13 | 4.48(3.84-5.13) | 1.24(0.03-6.65) | 52.68 | −1.07(−1.5-−0.65) |
| Zambia | 759.08(617.19-930.04) | 383.1 | 9.69(6.83-12.62) | 164.92(144.02-190.91) | −61.89 | −9.57(−10.89-−8.23) |
| Zimbabwe | 1200.88(870.14-1510.59) | 800.9 | 15.74(11.87-19.74) | 180.62(163.86-200.22) | −80.13 | −13.34(−14.31-−12.35) |

EAPC: estimated annual percentage change; ASR, age-standardized rate; CI, confidence interval; UI: uncertainty interval

**Supplementary table 9**. Age-standardized rates of DALYs caused by HIV/AIDS at the national level for both sexes in 2019, and percentage changes in the number of cases and the EAPCs from 1990 to 2019.

|  | **1990−2004** | | | **2005−2019** | | |
| --- | --- | --- | --- | --- | --- | --- |
| **Characteristics** | ASR/100,000  (95% UI) | Change in  number (%) | EAPC  (95%CI) | ASR/100,000  (95% UI) | Change in  number (%) | EAPC  (95%CI) |
| Afghanistan | 36.44(3.12-149.83) | 285.25 | 4.68(4.15-5.21) | 53.15(1.06-251.49) | 153.83 | 2.23(1.86-2.61) |
| Albania | 3.25(3.05-3.49) | 38.16 | 1.79(0.96-2.63) | 3.13(2.7-3.69) | −17.51 | −1.08(−1.37-−0.8) |
| Algeria | 37.64(6.39-239.82) | 317.25 | 8.43(8.05-8.82) | 30.64(2.66-209.41) | 1.89 | −2.47(−4-−0.93) |
| American Samoa | 53.4(30.49-92.76) | 510.33 | 10.49(8.59-12.42) | 50.32(10.11-134.06) | −19.76 | −0.55(−1.11-0.02) |
| Andorra | 139.75(12.48-585.29) | 104.43 | 1.05(0.12-2) | 119.25(7.6-509.6) | −8.91 | −1.17(−1.47-−0.88) |
| Angola | 3256.25(2134.15-4766.37) | 3092.95 | 24.31(21.38-27.31) | 3880.77(2701.61-5566.83) | 66.26 | 0.77(0.38-1.17) |
| Antigua and Barbuda | 585.16(565.19-615.33) | 53.58 | 1.64(−0.08-3.39) | 363.67(350.83-377.41) | −20.49 | −1.47(−2.44-−0.49) |
| Argentina | 263.04(249.76-281.79) | 209.02 | 6.57(3.3-9.95) | 214.5(189.95-253.66) | 12.99 | −0.84(−1.12-−0.55) |
| Armenia | 4.46(4.05-4.96) | 102.57 | 2.27(0.18-4.4) | 31.44(29.47-33.86) | 360.64 | 10.35(6.39-14.45) |
| Australia | 31.71(29.67-34.05) | −63.4 | −12.32(−15.17-−9.38) | 15.16(13.04-18.34) | −26.92 | −4.43(−5.62-−3.24) |
| Austria | 35.75(32.17-40.79) | −10.43 | −6.81(−10.36-−3.13) | 28.32(22.34-36.33) | −23 | −1.35(−2.38-−0.32) |
| Azerbaijan | 35.5(30.43-39.85) | 264.53 | 7.8(6.92-8.7) | 19.14(15.63-21.66) | −33.41 | −5.11(−6.82-−3.37) |
| Bahamas | 2857.93(2769.18-2983.98) | 202.56 | 4.34(1.24-7.53) | 1328.96(1283.96-1389.41) | −33.52 | −3.77(−4.69-−2.83) |
| Bahrain | 52.03(48.46-55.45) | 125.34 | 2.2(0.09-4.34) | 18.73(15.02-24.54) | −27.26 | −6.45(−7.23-−5.67) |
| Bangladesh | 4.18(1-15.39) | 5069.42 | 43.98(40.01-48.08) | 13.25(1.03-78.55) | 192.55 | 6.74(4.6-8.93) |
| Barbados | 639.28(618.96-670.73) | 0.97 | 0.42(−2.05-2.96) | 371.68(357.78-388.47) | −35.95 | −3.41(−4.02-−2.8) |
| Belarus | 115.94(109.94-123.15) | 155.97 | 6.54(5.22-7.87) | 150.51(140.48-162.32) | 17.06 | 0.35(−1.17-1.9) |
| Belgium | 46.01(41.53-52.02) | −15.07 | −5.05(−8.07-−1.94) | 30.92(24.68-38.72) | −11.51 | −1.59(−2.19-−0.98) |
| Belize | 1803.59(1751.94-1875.31) | 167.19 | 5.98(4.29-7.69) | 1052.94(1017.63-1098.95) | 15.05 | −1.53(−2.4-−0.64) |
| Benin | 4269.1(3266.94-5374.14) | 6859.64 | 29.46(20.8-38.74) | 1141.54(863.24-1495.88) | −58.71 | −8.69(−10.57-−6.77) |
| Bermuda | 652.77(629.24-687.86) | −26.58 | −1.7(−3.46-0.1) | 372.59(359.79-388.04) | −32.69 | −1.93(−2.83-−1.02) |
| Bhutan | 184.21(12.2-780.1) | 283.55 | 8.03(6.48-9.6) | 217.43(4.48-1525.42) | 41.73 | 1.34(0.82-1.86) |
| Bolivia  (Plurinational State of) | 419.03(84.02-1403.34) | 188.94 | 4.7(3.31-6.1) | 349.5(19.74-2182.56) | 22.31 | −0.73(−1.53-0.07) |
| Bosnia and Herzegovina | 3.92(3.6-4.21) | 6.05 | 0.6(0.01-1.19) | 2.15(1.58-3.23) | −49.42 | −1.29(−2.79-0.23) |
| Botswana | 50756.2(42928.42-61480.53) | 931.31 | 16.12(11.76-20.66) | 12487.43(10432.61-15741.47) | −58.21 | −8.06(−8.99-−7.13) |
| Brazil | 427.5(416.56-440.66) | 67.33 | 0.15(−1.98-2.32) | 337.12(323.13-354.69) | −0.1 | −1.49(−1.62-−1.37) |
| Brunei Darussalam | 47.04(43.58-51.16) | 308.01 | 5.92(4.92-6.93) | 62.55(51.97-76.08) | 62.26 | 0.9(0.12-1.69) |
| Bulgaria | 36.77(34.83-39.19) | 117.3 | 7.48(4.17-10.9) | 29.66(27.93-31.5) | −49.79 | −4.12(−4.91-−3.32) |
| Burkina Faso | 6426.54(5484.18-7693.48) | −4.68 | −3.11(−4.78-−1.42) | 905.55(734.43-1141.52) | −73.47 | −11.7(−12.18-−11.22) |
| Burundi | 13736.06(11043.59-16915.93) | 94.99 | 3.55(0.64-6.54) | 1212.63(1004.74-1504.31) | −83.07 | −15.07(−15.79-−14.33) |
| Cabo Verde | 2176.75(1224.45-3511.93) | 402.11 | 9.2(6.64-11.82) | 526.55(272.12-1159.63) | −64.55 | −8.77(−9.15-−8.38) |
| Cambodia | 3568.81(2581.21-4853.45) | 53209.92 | 50.14(34.51-67.58) | 466.99(310.95-675.46) | −80.81 | −13.09(−13.91-−12.26) |
| Cameroon | 13599.57(10312.61-17335.94) | 2562.71 | 22.57(18.43-26.86) | 5271.58(4624.36-6257.43) | −41.4 | −5.75(−6.77-−4.73) |
| Canada | 67.23(60.13-78.5) | −41.21 | −8.36(−11.4-−5.21) | 42.03(34.99-52.72) | −35.54 | −3.86(−5.07-−2.64) |
| Central African Republic | 20735.8(16502.1-25444.41) | 413.02 | 8.81(4.83-12.95) | 6810.68(5458.63-9043.27) | −57.36 | −6.93(−7.59-−6.26) |
| Chad | 5223.88(3498.65-7535.68) | 735.19 | 13.23(10.28-16.26) | 2276.38(1580.15-3258.27) | −32.6 | −4.59(−5.97-−3.19) |
| Chile | 133.03(126.78-142.3) | 267.09 | 9.2(5.7-12.81) | 133.45(120.63-158.29) | 22.06 | 0.82(0.13-1.53) |
| China | 46.21(42.65-51.34) | 335.06 | 8.56(7.51-9.61) | 82.28(66.65-98.84) | 85.14 | 4.19(3.2-5.18) |
| Colombia | 338.18(326.68-353.43) | 446.72 | 10.71(9.17-12.27) | 246.46(232.54-261.43) | −10.52 | −1.95(−2.18-−1.72) |
| Comoros | 12.78(4.35-48.22) | 728.88 | 10.09(9.04-11.16) | 18.72(3.75-108.02) | 74.13 | 0.71(−0.08-1.5) |
| Congo | 14207.44(11342.87-17827.38) | 120.81 | 2.87(1.12-4.65) | 4649.59(3857.99-5689.52) | −47.8 | −6.9(−7.37-−6.42) |
| Cook Islands | 120.52(11.45-638.44) | 714.75 | 15.14(14.46-15.81) | 440.76(8.57-2120.65) | 207.45 | 8.74(8.52-8.96) |
| Costa Rica | 187.02(179.71-196.82) | 129.01 | 1.6(−1.03-4.29) | 147.89(140.23-156.61) | −4.14 | −1.21(−2.24-−0.18) |
| Croatia | 6.58(6.16-7.08) | −4.79 | −1.16(−5.2-3.05) | 8.35(7.69-9.13) | 54.51 | 1.05(−2.95-5.21) |
| Cuba | 48.94(46.88-51.93) | 90.74 | 2.12(−0.34-4.65) | 141.22(132.09-153.88) | 152.47 | 8.88(6.62-11.18) |
| Cyprus | 10.45(9.86-11.03) | 311.22 | 7.72(6.67-8.77) | 12.86(11.26-14.96) | 63.34 | 1.17(0.72-1.63) |
| Czechia | 2.76(2.53-3.06) | 14.43 | −0.38(−4-3.37) | 8.33(7.74-9.09) | 81.03 | 5.68(3.36-8.04) |
| Cote d'Ivoire | 18481.21(13677.65-23916.16) | 367.78 | 8.27(4.89-11.76) | 3309.02(2810.28-3982.69) | −73.27 | −10.38(−11.44-−9.32) |
| Democratic People's Republic of Korea | 71.4(4.54-497.99) | 881.86 | 16.8(15.39-18.23) | 133.55(5.61-678.84) | 87.72 | 4.85(3.87-5.84) |
| Democratic Republic of the Congo | 5403.14(4091.96-6972.34) | 130.26 | 3.45(1.69-5.24) | 796.38(611.33-1060.22) | −75.88 | −11.92(−13.38-−10.43) |
| Denmark | 38.98(35.46-43.94) | 556.11 | 9.91(−3.11-24.69) | 23.21(19.27-28.84) | −27.33 | −2.26(−3.29-−1.21) |
| Djibouti | 7002.39(5048.97-9393.08) | 66949.11 | 53.05(40.35-66.91) | 3863.82(2731.16-5520.77) | −15.12 | −4.65(−5.28-−4.01) |
| Dominica | 561.95(543.17-589.76) | 29.46 | 2.89(1.31-4.51) | 373.24(359.52-389.39) | −18.28 | −0.53(−1.5-0.45) |
| Dominican Republic | 2176.23(1645-2804.65) | 719.46 | 12.4(7.94-17.05) | 614.24(435.32-920.26) | −62.02 | −7.63(−8.13-−7.14) |
| Ecuador | 315.92(303.53-328.88) | 899.7 | 14.81(12.7-16.96) | 369.93(343.79-401.72) | 33.46 | 0.1(−0.39-0.59) |
| Egypt | 9.05(8.37-9.69) | 88.52 | 2.19(1.09-3.32) | 3.52(2.84-4.47) | −47.13 | −6.15(−7.33-−4.95) |
| El Salvador | 752.7(610.36-856.27) | 691.58 | 16.06(14.84-17.3) | 591.18(313.26-852.91) | −7.56 | −1.39(−2.01-−0.76) |
| Equatorial Guinea | 9481.99(6363.95-14007.93) | 3944.67 | 25.85(22.79-28.99) | 10386.15(6554.59-16650.6) | 73.88 | 0.29(−0.49-1.07) |
| Eritrea | 5328.97(3660.49-7245.2) | 593.03 | 11.23(7.62-14.97) | 1301.64(938.12-1771.64) | −63.81 | −9.21(−9.59-−8.83) |
| Estonia | 84.89(80.08-89.85) | 2990.43 | 26.95(21.23-32.93) | 157.91(146.69-169.16) | 9.31 | −0.11(−1.77-1.58) |
| Eswatini | 64062.85(45515.75-84379.23) | 25734.94 | 44.08(34.25-54.62) | 18325.88(16076.55-21603.97) | −67.89 | −9.2(−9.67-−8.73) |
| Ethiopia | 9209.83(6815.72-11907.29) | 446.01 | 10.99(7.13-14.99) | 1699.06(1423.54-2050.66) | −68.68 | −10.62(−11.39-−9.83) |
| Fiji | 96.91(84.55-110.81) | 438.3 | 11.42(9.68-13.19) | 60.12(39.13-92.6) | −31.49 | −3.15(−3.69-−2.61) |
| Finland | 9.83(9.26-10.54) | −23.08 | −6.35(−9.95-−2.61) | 5.6(4.86-6.62) | −28.6 | −4.69(−6.99-−2.33) |
| France | 80.93(76.61-87.3) | −64.56 | −12.6(−15.93-−9.14) | 34.51(30.12-40.51) | −46.78 | −4.95(−6.35-−3.52) |
| Gabon | 10299.96(6732.98-15295.16) | 1220.9 | 17.88(14.3-21.58) | 3580.88(2583.34-5128.86) | −50.7 | −7.1(−7.84-−6.35) |
| Gambia | 4671.68(2993.56-6955.14) | 3555.25 | 26.46(22.81-30.23) | 3286.17(2227.12-4724.27) | −9.45 | −2.93(−3.49-−2.37) |
| Georgia | 3.32(2.79-4.3) | 18.06 | 3.28(1.97-4.61) | 38.36(34.07-47.04) | 1064.86 | 24.3(15.87-33.34) |
| Germany | 32.96(30.87-36.31) | −62.85 | −11.24(−13.88-−8.52) | 23.48(20.84-28.03) | −23.87 | −2.15(−2.4-−1.9) |
| Ghana | 6913.13(5488.74-8573.14) | 669.35 | 13.05(10.44-15.72) | 2701.11(2238.54-3356.92) | −43.84 | −6.49(−6.74-−6.25) |
| Greece | 7.93(7.29-8.69) | −51.83 | −8.39(−11.5-−5.17) | 13.01(11.68-14.79) | 79.64 | 7.86(3.69-12.2) |
| Greenland | 194.63(177.8-213.36) | 7.3 | −2(−3.85-−0.12) | 149.58(117.4-186.1) | −30.01 | −2.45(−3.01-−1.89) |
| Grenada | 404.5(390.06-424.68) | 27.82 | 0.43(−0.84-1.71) | 248.68(238.13-261.78) | −26.47 | −1.45(−2.35-−0.55) |
| Guam | 166.73(107.6-252.13) | 291 | 7.13(5.07-9.22) | 213.52(84.63-450.75) | 23.51 | 1.34(0.99-1.69) |
| Guatemala | 393.56(379.11-411.17) | 206.09 | 4.15(3.36-4.94) | 225.08(211.27-245.27) | −23.53 | −5.52(−6.52-−4.52) |
| Guinea | 3628.28(2579.28-4982.66) | 1498.64 | 20.05(17.25-22.91) | 2290.98(1612.15-3266.8) | −18.72 | −3.07(−3.61-−2.53) |
| Guinea-Bissau | 7442.94(4914.66-10942.48) | 2142.64 | 23.1(19.69-26.61) | 3637.35(2136.52-5971.48) | −40.13 | −5.45(−5.76-−5.14) |
| Guyana | 2401.13(2328.09-2502.99) | 275.99 | 12.83(10.02-15.72) | 1266.15(1218.88-1320.51) | −42.61 | −3.45(−4.2-−2.7) |
| Haiti | 9611.53(7192.81-12454.85) | 324.28 | 7.63(4.56-10.8) | 2671.5(2103.49-3449.95) | −59.02 | −8.23(−8.78-−7.67) |
| Honduras | 145.93(124.21-176.67) | 80.8 | 3.38(1.18-5.64) | 61.55(47.06-80.88) | 0.1 | −2.23(−2.87-−1.59) |
| Hungary | 38.02(36.22-40.03) | −33.49 | −5.38(−7.47-−3.23) | 13.03(12.22-13.92) | −59.51 | −6.94(−8.62-−5.23) |
| Iceland | 24.87(23.5-26.5) | −27.82 | −6.27(−8.9-−3.57) | 16(14.29-18.14) | −20.78 | −2.54(−3.04-−2.04) |
| India | 812.59(649.17-1001.23) | 8884.12 | 39.25(32.87-45.95) | 181.17(158.88-211.12) | −71.84 | −11.65(−12.48-−10.81) |
| Indonesia | 81.64(69.61-94.68) | 17131.68 | 24.92(13.38-37.64) | 160.38(126.69-203.15) | 91.01 | 4.63(4-5.28) |
| Iran  (Islamic Republic of) | 26.3(23.13-29.25) | 600.76 | 11.82(11.07-12.57) | 69.04(51.03-94.6) | 211.85 | 6.41(6.06-6.77) |
| Iraq | 10.04(4.85-26.85) | 642.74 | 11.32(11.02-11.63) | 12.28(2.36-48.66) | 54.19 | 0.99(0.26-1.72) |
| Ireland | 16.77(15.6-18.3) | 17.96 | −7.46(−13.02-−1.54) | 11.84(10.19-14.13) | 14.79 | −1.02(−4.2-2.26) |
| Israel | 26.95(23.96-30.96) | −8.78 | −5.11(−7.02-−3.16) | 28.58(23.1-35.65) | 34 | −0.14(−1.34-1.08) |
| Italy | 86.49(81.19-95.06) | −44.94 | −10.79(−15.08-−6.28) | 43.52(38.92-50.2) | −41.9 | −4.33(−5.06-−3.6) |
| Jamaica | 1251.99(1216.23-1310.32) | 240.1 | 8.48(6.79-10.2) | 706.25(684.95-732.13) | 11.35 | −1.49(−2.79-−0.16) |
| Japan | 6.17(5.61-6.95) | 152.65 | 6.58(3.94-9.29) | 5.98(4.96-7.27) | −2.26 | −0.7(−1.11-−0.28) |
| Jordan | 15.22(13.88-16.5) | 411.77 | 10.56(9.87-11.25) | 13.88(10.52-18.43) | 102.43 | 2.22(0.98-3.49) |
| Kazakhstan | 83.42(79.76-88.61) | 199.79 | 7.17(3.02-11.49) | 73.88(69.06-79.31) | −6.37 | −2.09(−2.63-−1.56) |
| Kenya | 26885.65(22175.12-31762.57) | 414.58 | 9.62(6.23-13.12) | 6464.76(5725.93-7385.1) | −61.13 | −9.25(−9.67-−8.82) |
| Kiribati | 100.98(91.02-110.71) | 115.17 | 3.49(2.21-4.78) | 84.2(63.93-109.02) | 8.88 | −1.23(−1.8-−0.66) |
| Kuwait | 6.07(5.54-6.7) | 8.97 | −2.82(−7.32-1.9) | 3.76(3.3-4.27) | −46.15 | −7.42(−10.4-−4.34) |
| Kyrgyzstan | 131.41(125.69-137.86) | 296.42 | 10.44(7.63-13.32) | 144.93(136.62-155.17) | 21.88 | 1.08(−0.35-2.54) |
| Lao People's Democratic Republic | 213.33(24.6-1107.48) | 430070.08 | 59.08(43.66-76.17) | 298.97(18.88-2032.78) | 53.59 | 1.13(−0.71-3) |
| Latvia | 165.84(157.58-176.58) | 93.3 | 5.03(2.64-7.46) | 289.36(273.2-308.69) | 37 | 3.53(2.05-5.04) |
| Lebanon | 93.7(3.39-453.65) | 26.55 | 0.69(−0.38-1.77) | 67.22(1.7-310.93) | 3.52 | −2.18(−2.61-−1.75) |
| Lesotho | 57561.72(43072.03-74663.97) | 2719.29 | 26.26(21.81-30.87) | 28922.04(24609.45-35669.26) | −42.09 | −4.19(−5.14-−3.23) |
| Liberia | 5035.03(3346.55-7387.49) | 1675.21 | 18.95(15.57-22.43) | 2072.9(1596.51-2763.27) | −37.94 | −5.44(−6.06-−4.82) |
| Libya | 48.61(3.7-228.67) | 399.5 | 10.06(9.87-10.25) | 62.09(2.12-350.83) | 55.45 | 0.97(0.49-1.45) |
| Lithuania | 99.3(94.33-105.49) | 196.57 | 8.07(5.13-11.08) | 93.76(88.37-99.83) | −1.93 | 0.88(−0.36-2.14) |
| Luxembourg | 36.84(34.06-40.7) | −29.54 | −7.51(−10.5-−4.43) | 22.23(19.27-26.55) | −11.1 | −2.91(−3.56-−2.26) |
| Madagascar | 407.68(251.74-665.84) | 57863.96 | 58.05(49.41-67.18) | 600.41(386.58-876.69) | 89.16 | 1.94(1.3-2.59) |
| Malawi | 40636.54(32959.62-49311.8) | 368.65 | 10.01(7.26-12.82) | 5835.24(5060.2-6934.59) | −79.04 | −13.18(−13.72-−12.65) |
| Malaysia | 420.78(386.13-453.17) | 3148.99 | 21.56(15.07-28.42) | 305.07(184.91-426.33) | −14.62 | −0.88(−2.5-0.77) |
| Maldives | 10.47(9.44-11.54) | 423.55 | 9.96(9.7-10.22) | 8.51(7.69-9.4) | 54.18 | −0.95(−1.71-−0.2) |
| Mali | 3639.56(2686.06-4826.42) | 1005.76 | 16.33(12.92-19.85) | 1536.37(1142.03-2005.13) | −33.45 | −4.54(−5.58-−3.48) |
| Malta | 26.01(24.1-28.27) | −14.36 | −5.2(−8.27-−2.04) | 21.26(17.63-26.73) | −6.96 | −1.14(−1.39-−0.89) |
| Marshall Islands | 193.26(4.86-1376.65) | 253.05 | 7.44(5.49-9.42) | 366.82(3.15-2903) | 108.73 | 4.43(3.88-4.97) |
| Mauritania | 118.17(20.74-693.29) | 1.16 | −3.38(−4.5-−2.25) | 40(6.76-253.52) | −44.72 | −5.59(−6.81-−4.35) |
| Mauritius | 69.39(63.94-77.25) | 81.7 | 11.01(2.21-20.56) | 310.35(288.65-336.07) | 270.07 | 9.3(6.02-12.69) |
| Mexico | 278.16(271.21-290.61) | 157.69 | 3.15(1.36-4.96) | 201.82(195.29-210.5) | −9.66 | −2.58(−2.93-−2.23) |
| Micronesia  (Federated States of) | 456.44(135.42-2169.03) | −5.14 | 0.67(0-1.35) | 2359.74(100.35-11824.04) | 289.78 | 11.21(10.66-11.76) |
| Monaco | 74.56(20.71-195.81) | 60.35 | −0.35(−1.28-0.6) | 73.02(13.53-241.97) | 4.45 | −0.22(−0.54-0.11) |
| Mongolia | 11.93(7.7-17.57) | 38029.46 | 44.3(33.1-56.43) | 19.93(2.92-49.69) | 154.23 | 4.72(4.02-5.43) |
| Montenegro | 24.95(20-28.85) | 227.27 | 9.48(7.75-11.23) | 15.53(12.71-18.63) | −45.35 | −2.49(−3.19-−1.79) |
| Morocco | 149.28(9.43-683.04) | 594.56 | 13.45(12.45-14.46) | 79.73(2.97-464.65) | −41.81 | −4.42(−5.76-−3.05) |
| Mozambique | 23130.19(17592.57-30041.85) | 2626.12 | 23.57(20.34-26.88) | 15888.85(13127.87-20542.99) | −11.92 | −2.69(−3.37-−2) |
| Myanmar | 1601.89(1252.59-2037.33) | 18084.15 | 36.94(27.34-47.26) | 463.13(364.01-604.52) | −68.21 | −9.19(−11.1-−7.25) |
| Namibia | 35451.94(28400.27-44554.61) | 3169.32 | 26.15(21.45-31.04) | 9404.07(8253.3-10986.48) | −63.65 | −7.4(−8.81-−5.96) |
| Nauru | 124.4(12.71-665.42) | 721.52 | 15.51(14.88-16.14) | 443.49(10.44-2145.36) | 190.72 | 8.54(8.29-8.79) |
| Nepal | 323.24(21.57-1490.77) | 1475396.39 | 86.23(69.87-104.17) | 260.96(6.03-1511.83) | −19.27 | −3.4(−5.08-−1.7) |
| Netherlands | 32.5(29.47-37.15) | −60.65 | −10.61(−13.38-−7.75) | 18.83(15.17-23.49) | −32.29 | −2.25(−3.19-−1.3) |
| New Zealand | 19.06(17.64-21.11) | −67.53 | −11.93(−13.84-−9.99) | 13.07(10.9-16.08) | −25.05 | −1.81(−3.89-0.31) |
| Nicaragua | 184.49(155.64-208.1) | 622.2 | 13.61(12.66-14.57) | 468.91(296.47-646.09) | 191.83 | 7.39(5.7-9.12) |
| Niger | 2086.21(1535.49-2780.44) | 970.46 | 14.99(11.45-18.65) | 517.77(364.36-702.7) | −63.38 | −8.9(−9.6-−8.2) |
| Nigeria | 5176.36(3764.57-7154.26) | 1174.74 | 16.52(12.9-20.25) | 2618.12(2105.56-3276.18) | −26.85 | −4.73(−5.46-−4) |
| Niue | 124.25(11.73-655.09) | 485.01 | 15.41(14.87-15.96) | 428.12(9.47-2081.96) | 233.3 | 8.34(8.15-8.54) |
| North Macedonia | 6.7(5.48-7.95) | 256.3 | 9.64(9.04-10.25) | 7.41(5.89-9.23) | −13.18 | −0.9(−1.97-0.18) |
| Northern Mariana Islands | 72.99(39.59-130.55) | 782.83 | 12.95(11.21-14.72) | 73.18(12.43-182.51) | −49.18 | −0.94(−1.55-−0.34) |
| Norway | 26.89(25.41-29.09) | −16.6 | −7.42(−10.89-−3.81) | 16.22(13.81-19.53) | −30.27 | −2.36(−4.08-−0.62) |
| Oman | 64.89(43.25-88.32) | 530.88 | 12.43(10.33-14.57) | 98.93(57.88-151.15) | 198.09 | 0.4(−1.4-2.23) |
| Pakistan | 24.65(2.27-105.67) | 99.92 | 1.29(0.24-2.36) | 109.69(2.36-637.74) | 528.44 | 12.04(11.28-12.82) |
| Palau | 114.55(10.74-611.14) | 942.74 | 14.78(14.08-15.47) | 411.48(8.5-1989.4) | 209.54 | 8.56(8.3-8.82) |
| Palestine | 13.66(12.48-14.94) | 877.26 | 11.77(11.13-12.41) | 16.67(13.88-20.26) | 44.19 | 0.37(0.02-0.73) |
| Panama | 803.85(776.77-838.66) | 201.74 | 6.28(4.37-8.23) | 687.4(648.9-732.34) | 14.75 | −0.78(−1.32-−0.24) |
| Papua New Guinea | 2618.11(859.95-5844.09) | 109371.25 | 63.52(53.1-74.65) | 2227.84(637.39-6340.32) | 20.83 | −1.75(−2.08-−1.41) |
| Paraguay | 336.63(258.75-392.35) | 683.53 | 13.81(13.28-14.34) | 477.07(300.62-650.6) | 53.05 | 2.9(1.15-4.68) |
| Peru | 494.54(425.27-547.2) | 332.52 | 9.96(9.36-10.57) | 442.05(258.58-751.16) | 6.16 | 0.31(−0.84-1.47) |
| Philippines | 274.45(267.61-283.39) | 431.76 | 10.08(10.03-10.14) | 290.35(271.65-327.62) | 24.12 | −1.14(−1.72-−0.56) |
| Poland | 22.04(21.14-23.41) | 627.92 | 22.05(17.34-26.95) | 18.2(16.83-20.74) | −10.97 | −1.14(−2.21-−0.06) |
| Portugal | 430.98(412.91-452.88) | 349.45 | 8.53(3.45-13.85) | 193.97(180.94-210.97) | −54.84 | −6.06(−7.49-−4.61) |
| Puerto Rico | 666.11(639.54-711.99) | −44.92 | −7.83(−10.51-−5.07) | 261.55(252.25-271.16) | −62.37 | −6.77(−8.2-−5.32) |
| Qatar | 13.06(11.16-15) | −9.5 | −5.56(−8.91-−2.08) | 7.3(6.03-8.5) | 92.64 | −4.43(−5.01-−3.84) |
| Republic of Korea | 9.75(8.11-12.54) | 87.35 | 2.04(−1.26-5.45) | 12.56(9.66-17.03) | 52.35 | 0.73(−0.39-1.86) |
| Republic of Moldova | 256.98(245.59-270.26) | 293.22 | 9.25(7.44-11.08) | 200.43(187.09-215.64) | −23.68 | −3.02(−3.76-−2.28) |
| Romania | 78.74(75.27-82.33) | −6.23 | 2.54(−3.14-8.56) | 50.2(46.78-54.02) | −28.2 | 0.66(−1.05-2.41) |
| Russian Federation | 366.56(358.67-381.49) | 194.56 | 8.01(6.96-9.06) | 672.64(647.09-707.04) | 76.62 | 4.62(3.68-5.58) |
| Rwanda | 10792(8016.43-13876.24) | 493.12 | 12.42(8.26-16.75) | 1602.7(1377.23-1926.41) | −74.94 | −11.2(−12.03-−10.36) |
| Saint Kitts and Nevis | 1455.4(383.55-2933.1) | 802.52 | 13.76(11.67-15.89) | 2130.65(383.71-5367.92) | 81.19 | 2.66(2.53-2.79) |
| Saint Lucia | 330.79(319.33-347.95) | 30.97 | 1.38(−0.04-2.82) | 186.14(178.41-196.3) | −23.63 | −2.44(−3.21-−1.67) |
| Saint Vincent and the Grenadines | 1384.67(1342.56-1449.31) | 93.51 | 6.7(4.42-9.02) | 822.93(796.78-855.81) | −26.03 | −1.72(−2.8-−0.63) |
| Samoa | 182.14(5.55-1299.6) | 259.64 | 7.54(5.56-9.55) | 371.4(4.52-2959.53) | 133.13 | 5.12(4.69-5.55) |
| San Marino | 77.09(21.65-198.85) | 71.13 | 0.33(−0.84-1.52) | 70.86(12.73-235.69) | 7.26 | −0.64(−0.95-−0.34) |
| Sao Tome and Principe | 66.65(52.61-84.29) | 201.55 | 5.14(3.51-6.8) | 13.31(8.78-19.52) | −58.51 | −7.1(−8.92-−5.24) |
| Saudi Arabia | 90.03(56.21-201.18) | 374.48 | 8.92(8.69-9.14) | 82.84(30.17-257.38) | 62.09 | −0.93(−1.18-−0.68) |
| Senegal | 2190.18(1574.18-2942.78) | 1035.2 | 16.32(13.13-19.6) | 695.95(538.16-925.75) | −58.2 | −6.19(−7.75-−4.62) |
| Serbia | 36.11(29.68-51.09) | 85.65 | 4.31(3.5-5.12) | 28.42(18.59-56.15) | −11.66 | 0.45(−2.19-3.17) |
| Seychelles | 285.13(268.94-300.07) | 1473.62 | 16.79(11.93-21.85) | 163.65(128.72-212.71) | −24.82 | −1.96(−2.9-−1.01) |
| Sierra Leone | 3369.05(2439.37-4524.68) | 1826.3 | 20.64(17.54-23.83) | 2220.57(1759.13-2943.39) | −9.88 | −3.14(−3.68-−2.59) |
| Singapore | 40.28(36.14-46.9) | 390.95 | 9.95(5.11-15.02) | 24.17(20.97-30.11) | −3.75 | −3.42(−5.25-−1.54) |
| Slovakia | 5.58(4.28-6.88) | 416.35 | 10.27(7.1-13.54) | 4.34(3.25-5.41) | −5.37 | 0.31(−1.11-1.76) |
| Slovenia | 5.86(5.42-6.37) | −42.58 | −0.76(−5.94-4.71) | 5.74(5.2-6.47) | −26.09 | 1.32(−3.93-6.85) |
| Solomon Islands | 214.68(5.9-1456.69) | 311.43 | 7.41(5.56-9.29) | 370.66(4.22-2961.77) | 105.45 | 3.65(2.97-4.33) |
| Somalia | 2353.45(1617.04-3471.41) | 11334.74 | 34.27(26.41-42.61) | 1087.26(801.81-1488.01) | −25.86 | −5.58(−5.96-−5.19) |
| South Africa | 33611.74(25840.99-43063.42) | 7511.71 | 34.5(29.09-40.14) | 13060.16(11237.23-15907.93) | −53.43 | −7.29(−7.92-−6.65) |
| South Sudan | 4370.81(2057.15-8538.33) | 1672.25 | 20.49(17.29-23.77) | 3251.35(1368.31-6625.54) | −26.59 | −2.41(−3.01-−1.8) |
| Spain | 166.03(158.77-175.12) | −30.53 | −8.75(−12.76-−4.56) | 61.73(56.3-67.73) | −54.17 | −6.67(−8.03-−5.29) |
| Sri Lanka | 21.51(19.92-23.2) | 154.22 | 5.36(3.89-6.84) | 10.44(7.18-16.13) | −48.6 | −4.17(−5.04-−3.3) |
| Sudan | 760.84(396.73-1463.5) | 1404.78 | 18.24(15.25-21.31) | 786.51(387.66-1592.26) | 34.98 | 0.16(−0.21-0.53) |
| Suriname | 1745.56(1696.73-1815.57) | 175.52 | 8.26(6.51-10.04) | 869.87(842.6-907.1) | −38.15 | −3.74(−4.48-−2.99) |
| Sweden | 14.88(13.41-16.91) | −54.67 | −9.48(−13.13-−5.67) | 11.67(9.82-14.25) | −21.37 | −2.19(−3.67-−0.69) |
| Switzerland | 56.23(50.7-63.94) | 443.16 | 13.86(1.46-27.78) | 29.4(22.94-37.94) | −25.35 | −3.77(−4.71-−2.82) |
| Syrian Arab Republic | 8.22(7.29-9.16) | 169.94 | 5.11(4.62-5.6) | 6.52(5.1-8.39) | −45.77 | −0.85(−1.86-0.18) |
| Taiwan  (Province of China) | 17.02(15.89-18.39) | 765.31 | 12.34(6.73-18.23) | 29.35(26.71-32.21) | 36.53 | 2.2(0.44-4) |
| Tajikistan | 105.16(95.15-114.09) | 1193.15 | 16.98(14.32-19.7) | 37.05(28.18-52.5) | −44.16 | −8.15(−9.17-−7.11) |
| Thailand | 2237.24(2037.01-2428.38) | 2080.76 | 19.05(12.62-25.84) | 951.85(709.23-1498.01) | −46.65 | −2.59(−4.33-−0.81) |
| Timor-Leste | 1598.18(47.81-8693.96) | 887.41 | 14.09(7.8-20.75) | 1086.16(12.92-6354.92) | −5.76 | −1.98(−2.36-−1.59) |
| Togo | 12029.46(8798.04-15703.82) | 2264.64 | 22.58(17.84-27.51) | 2655.81(2011.38-3514) | −67.85 | −10.01(−10.77-−9.24) |
| Tokelau | 130.74(12.46-689.07) | 537.58 | 15.46(14.92-16) | 447.23(10.06-2163.87) | 229.35 | 8.27(8.05-8.49) |
| Tonga | 78.78(47.14-130.3) | 407.03 | 10.47(8.5-12.47) | 103.21(22.23-280.57) | 23.66 | 2.03(1.68-2.39) |
| Trinidad and Tobago | 1199.48(1162.04-1255.09) | 208.78 | 9.13(5.9-12.46) | 691.12(667.55-720.67) | −35.43 | −2.88(−3.63-−2.12) |
| Tunisia | 34.63(4.38-210.47) | 522.93 | 12.1(11.56-12.65) | 60.73(3.77-329.59) | 89.87 | 3.9(2.84-4.97) |
| Turkey | 7.99(7.12-9.28) | 1058.66 | 16.21(14.83-17.61) | 14.38(11.46-17.34) | 79.59 | 5.58(4.02-7.16) |
| Turkmenistan | 185.99(175.69-197.98) | 148.56 | 4.59(1.67-7.6) | 110.66(102.98-119.28) | −33.22 | −3.87(−4.5-−3.23) |
| Tuvalu | 120.76(12.52-637.78) | 636.47 | 15.11(14.64-15.59) | 436.53(10.24-2126.55) | 283.24 | 8.66(8.43-8.88) |
| Uganda | 24440.25(21529.72-28236.12) | 13.96 | −2.11(−2.73-−1.48) | 3925.56(3242.21-4909.4) | −75.44 | −12.43(−13.19-−11.66) |
| Ukraine | 577.06(551.59-614.11) | 293.27 | 11.33(9.57-13.12) | 675.61(637.63-716.14) | −26.27 | −3.46(−4.73-−2.16) |
| United Arab Emirates | 38.05(2.46-176.13) | 870.21 | 11.13(10.83-11.43) | 114.14(1.99-673.92) | 243.04 | 9.68(8.39-10.97) |
| United Kingdom | 29.19(25.36-34.48) | −23.14 | −6.01(−8.96-−2.98) | 27.34(21-36.17) | −0.24 | −1.58(−2.23-−0.92) |
| United Republic of Tanzania | 22324.94(17961.38-27184.35) | 218.78 | 5.59(3.36-7.87) | 3355.68(2834.72-4096.83) | −77.87 | −12.8(−13.36-−12.23) |
| United States of America | 217.09(201.43-237.81) | −50.76 | −9.31(−11.94-−6.59) | 108.87(91.54-131.79) | −40.9 | −4.73(−5.76-−3.68) |
| United States Virgin Islands | 496.72(480.85-520.84) | 62.05 | 2.43(0.86-4.04) | 315.18(305.17-326.83) | −34.41 | −1.38(−2.29-−0.45) |
| Uruguay | 229.98(219.29-243.26) | 280.87 | 9.86(8.19-11.55) | 228.51(213.2-250.36) | −5.18 | −0.72(−1.47-0.03) |
| Uzbekistan | 80.92(77.15-86.05) | 180.39 | 7.49(5.88-9.13) | 88.89(83.39-95.02) | 45.12 | −0.02(−0.75-0.71) |
| Vanuatu | 197.82(6.18-1304.52) | 265.34 | 6.71(4.96-8.5) | 377.6(5.42-3002.79) | 142.11 | 4.26(3.91-4.62) |
| Venezuela  (Bolivarian Republic of) | 333.85(322.71-348.58) | 112.2 | 3.42(1.99-4.87) | 316.41(298.22-337.21) | 9.89 | −0.26(−1.11-0.59) |
| Viet Nam | 414.25(335.07-476.83) | 1553.94 | 19.14(17.12-21.19) | 322.12(253.15-422.83) | −12.01 | −2.34(−2.87-−1.81) |
| Yemen | 68.32(7.28-282.11) | 185.76 | 4.32(3.74-4.91) | 69.27(2.08-347.73) | 53.94 | −0.51(−0.98-−0.04) |
| Zambia | 39202.11(32366.92-47796.83) | 342.39 | 8.99(6.27-11.79) | 8697.07(7565.02-10233.22) | −61.91 | −9.52(−10.75-−8.26) |
| Zimbabwe | 60387.82(44313.56-75211.83) | 659.02 | 14.49(10.84-18.26) | 9176.82(8369.9-10219.61) | −80.02 | −13.23(−14.11-−12.33) |

EAPC: estimated annual percentage change; ASR, age-standardized rate; CI, confidence interval; UI: uncertainty interval; DALYs: disability-adjusted life years.
